# Supplementary material for: Understanding and mitigating the impact of ambient mRNA contamination in single-cell RNA-sequencing analysis
Source: PLoS One. 2025 Sep 24;20(9):e0332440. doi: 10.1371/journal.pone.0332440 (PMC12459771; doi:10.1371/journal.pone.0332440)
Supplement: S1 File — (PDF) [file pone.0332440.s020.pdf]

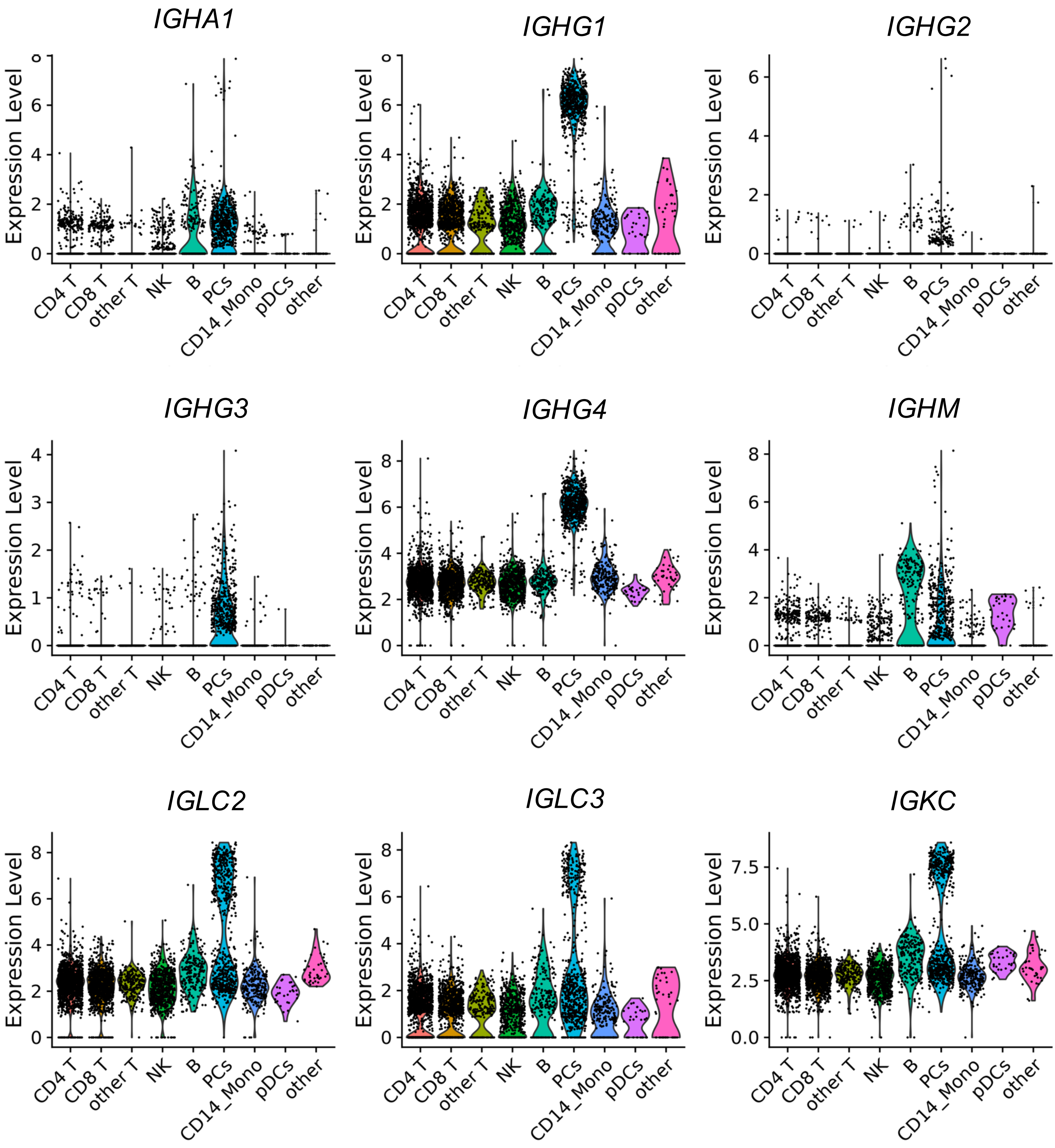

S1 Fig. Violin plots demonstrating the normalised expression levels of immunoglobulin (Ig) genes, canonical marker genes of B and plasma cells, across cell types annotated using Azimuth and known canonical marker genes (S1 Table) where relevant. Related to Fig 1.
PCs = plasma cells; pDCs = plasmacytoid dendritic cells.

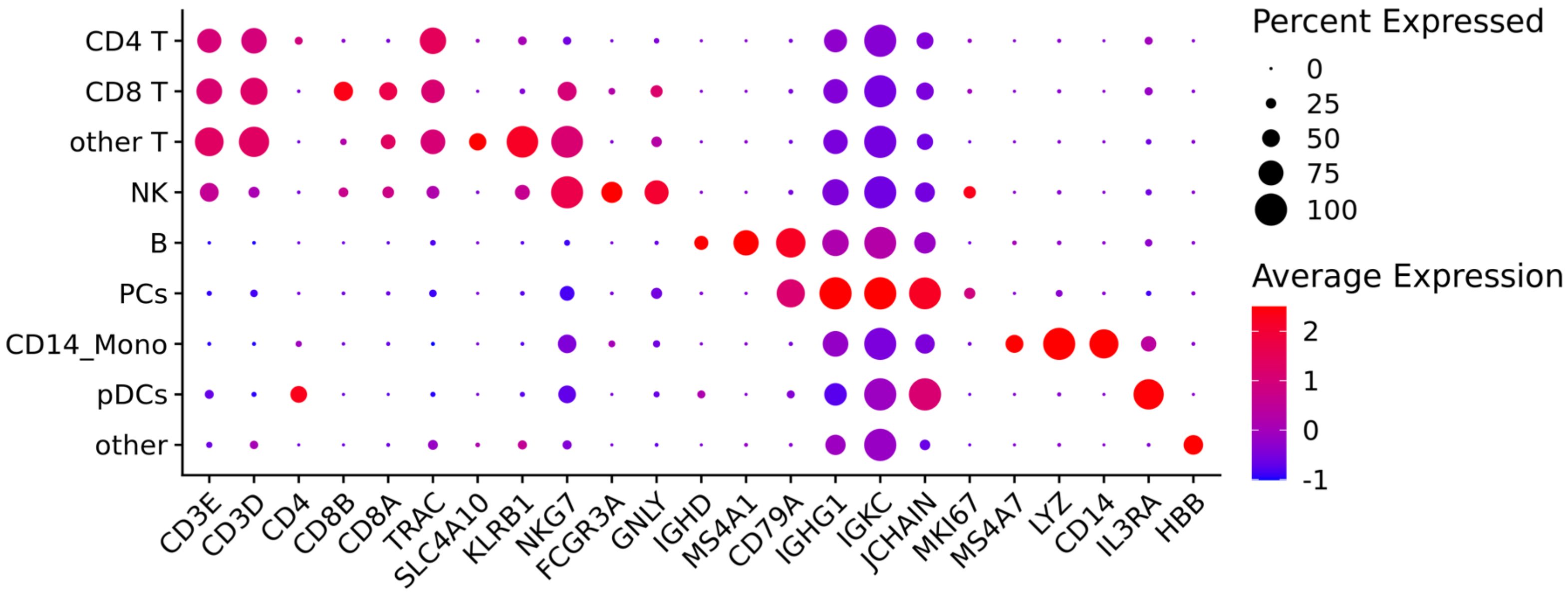

S2 Fig. Dotplot demonstrating the average expression levels of canonical marker genes in peripheral blood mononuclear cells (PBMCs) before ambient mRNA correction. Dot size represents the percentage of cells expressing the genes. Cell types are annotated using Azimuth and known canonical marker genes (S1 Table) where relevant. Related to Fig 1.
PCs = plasma cells; pDCs = plasmacytoid dendritic cells.

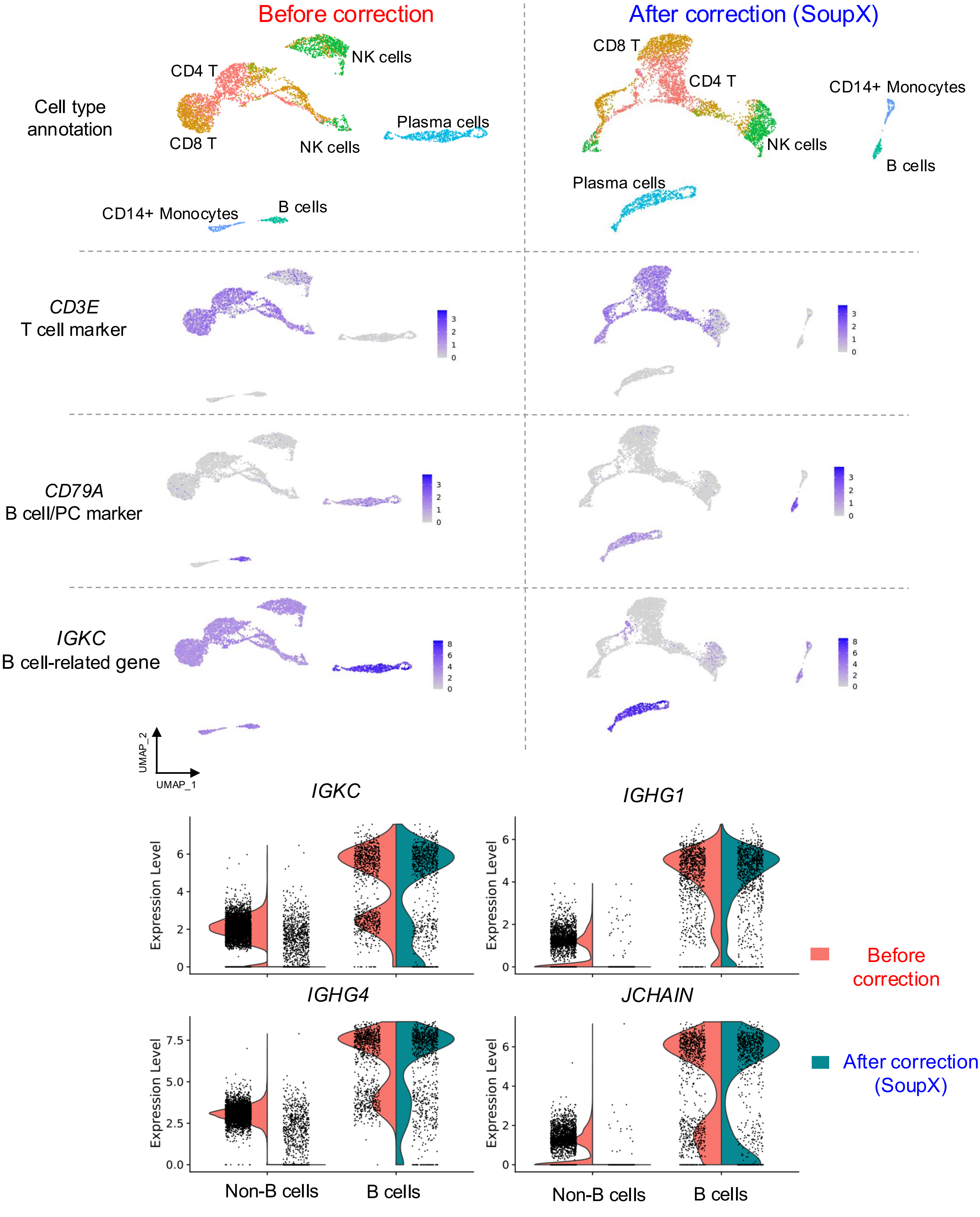

S3 Fig. UMAP plot showing the single-cell transcriptome profiles of PBMCs before (left panel) and after (right panel) ambient mRNA correction using SoupX. Violin plots showing the normalised transcription levels of the B cell-related genes, comparing between before and after ambient mRNA correction using SoupX. Related to Fig. 2.

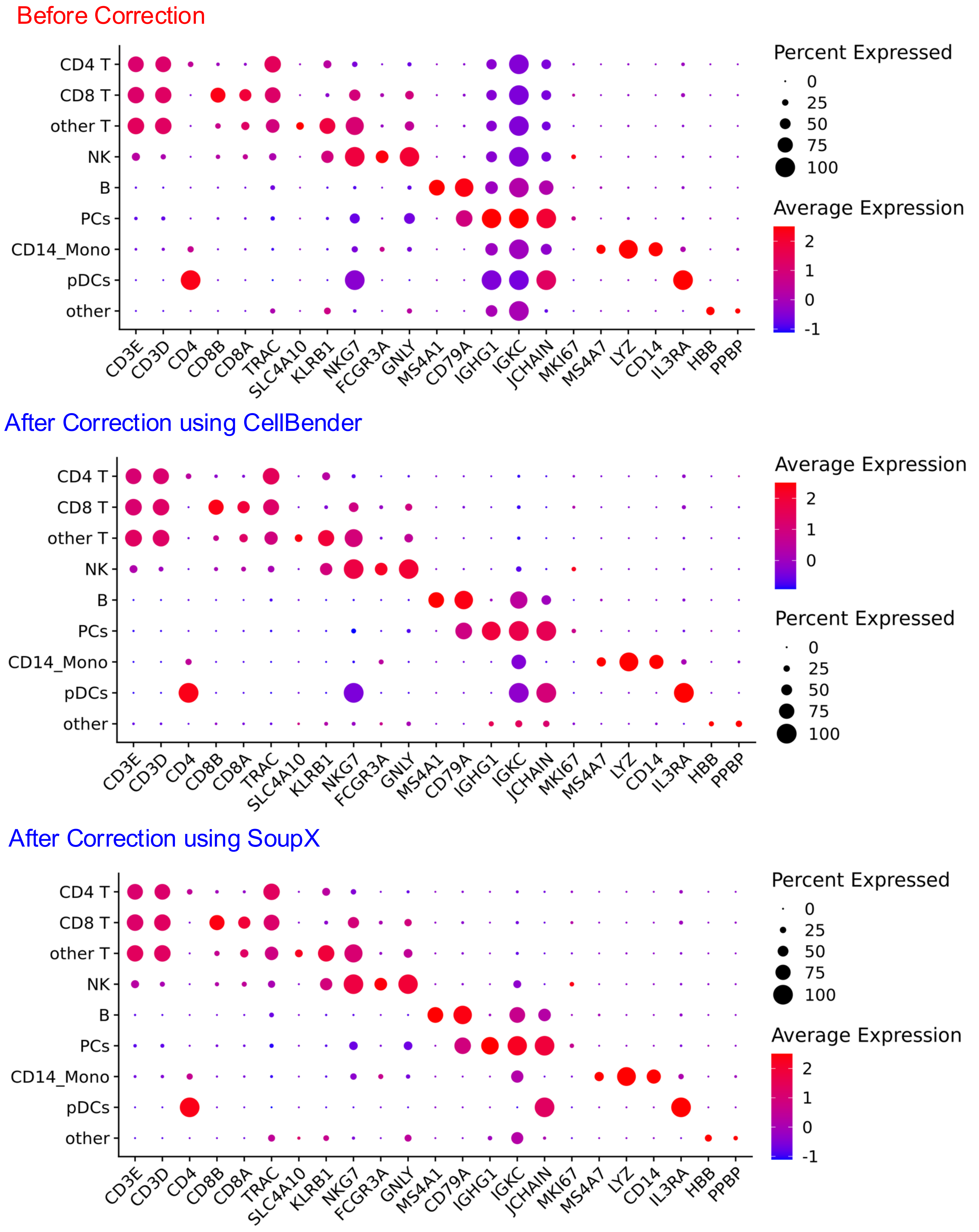

S4 Fig. Dotplot representing the average expression levels of canonical marker genes before (upper panel) and after ambient mRNA correction using CellBender (middle panel) and SoupX (lower panel) across the PBMCs. The dot sizes represent the percentages of cells expressing the genes. Cell types are annotated using Azimuth and known canonical marker genes (S1 Table) where relevant. Related to Fig 2.

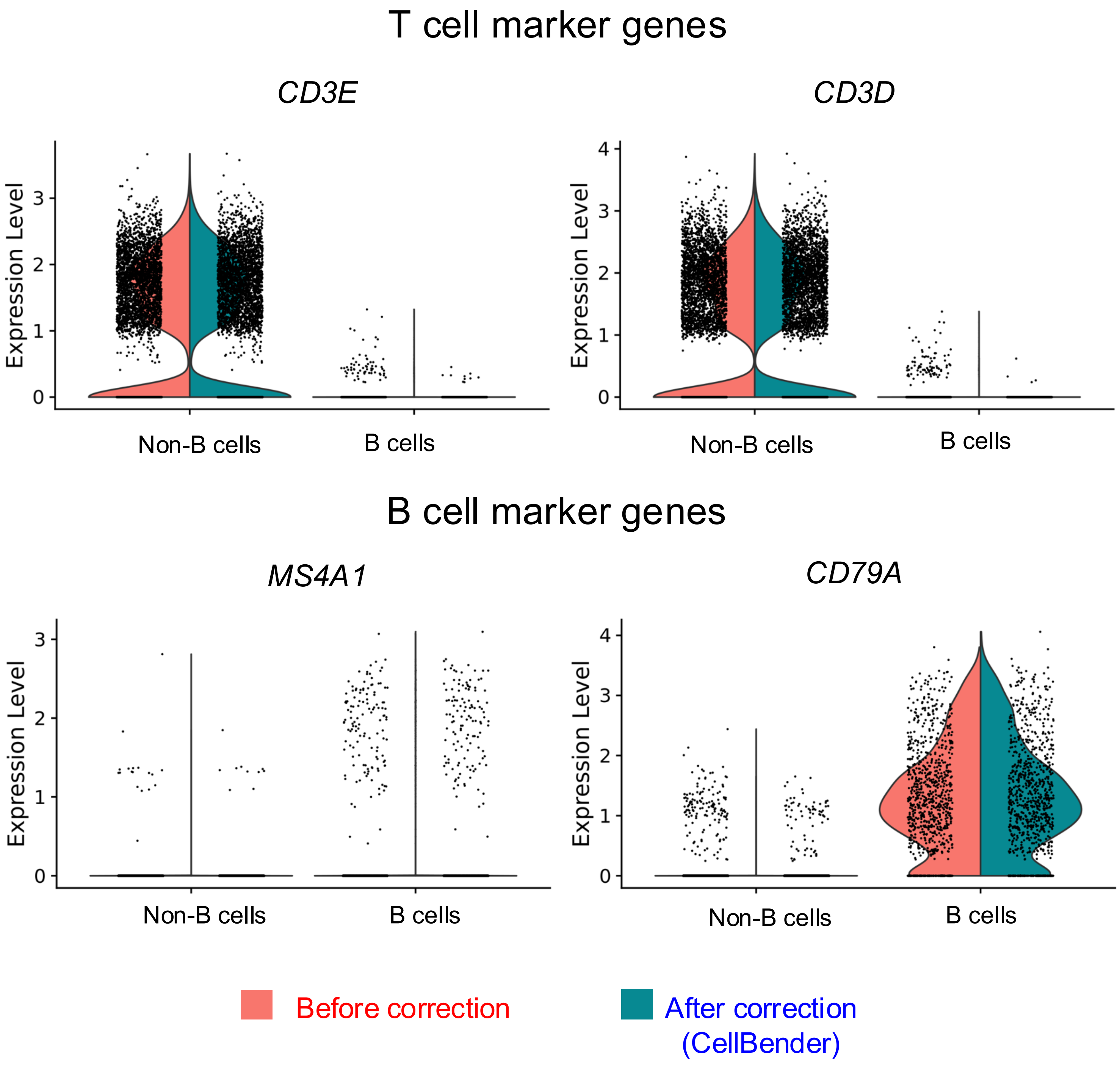

S5 Fig. Violin plots representing the normalised expression levels of the T cell (upper panel) and B cell (lower panel) marker genes in each non-B cell and B cell population, comparing before and after ambient mRNA correction using CellBender. Related to Fig 2.

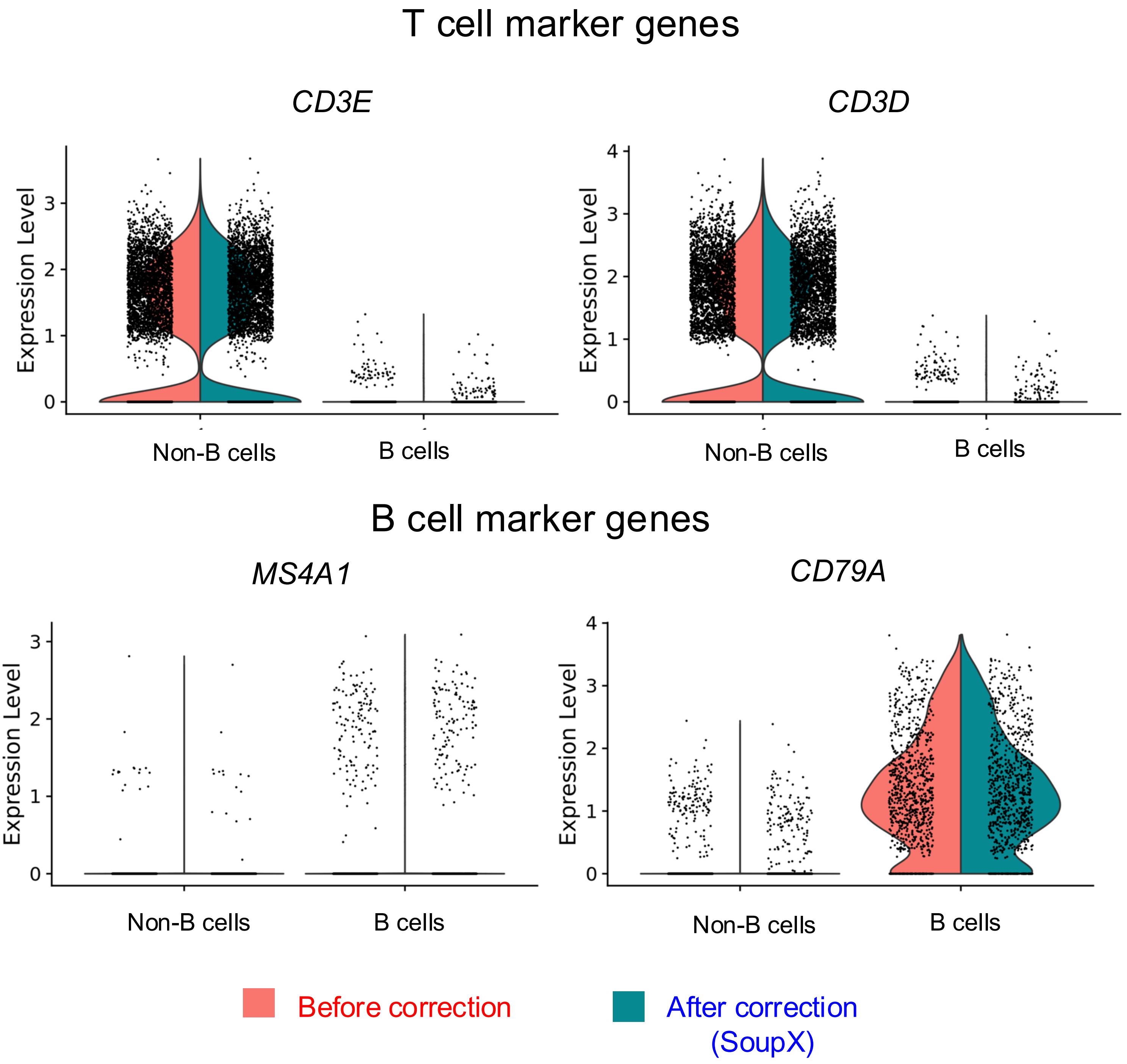

S6 Fig. Violin plots representing the normalised expression levels of the T cell (upper panel) and B cell (lower panel) marker genes in each non-B cell and B cell population, comparing before and after ambient mRNA correction using SoupX. Related to Fig 2.

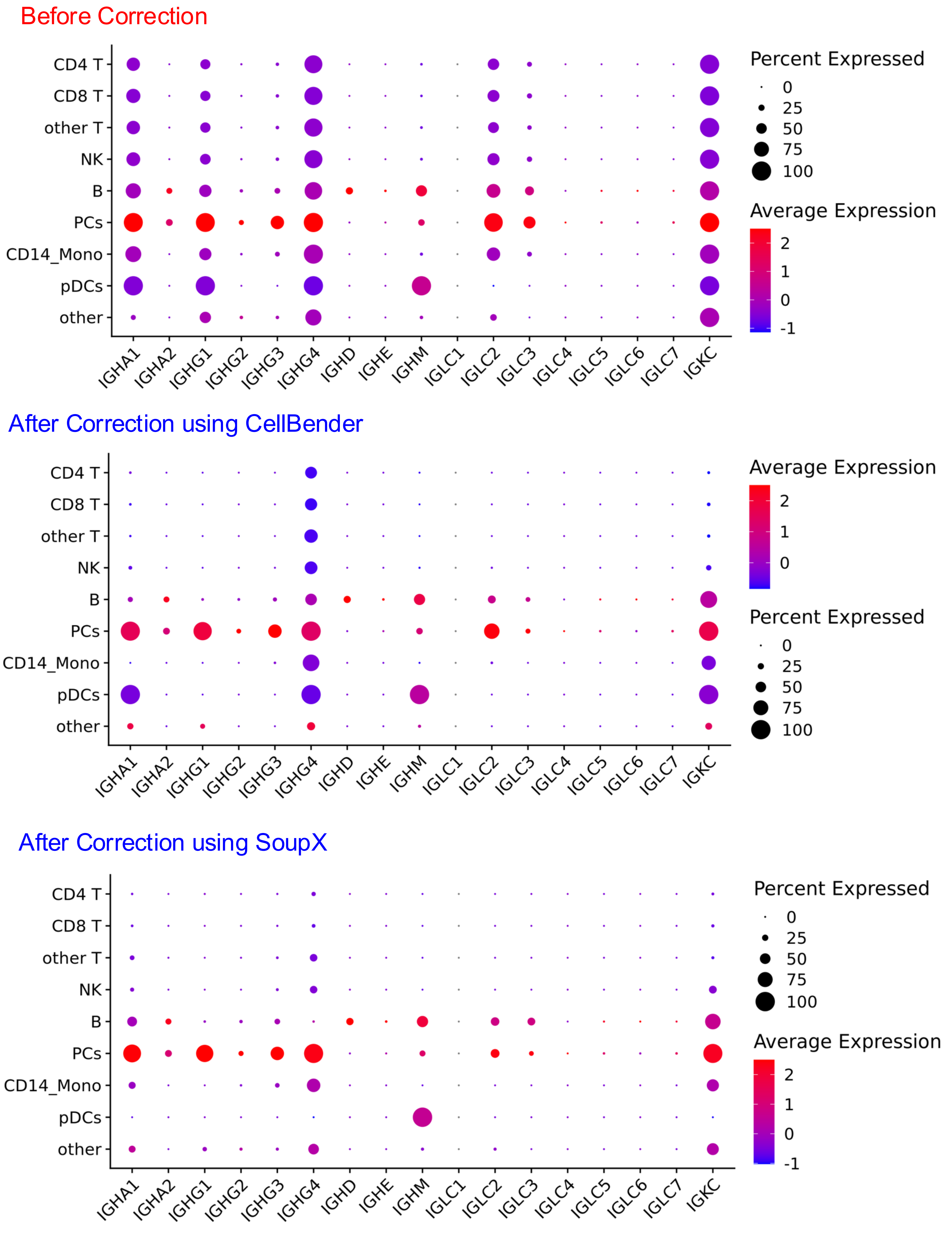

S7 Fig. Dotplot showing the average expression levels of immunoglobulin (Ig) genes before (upper panel) and after ambient mRNA correction using CellBender (middle panel) and SoupX (lower panel) across the PBMCs. The dot sizes represent the percentages of cells expressing the genes. Cell types are annotated using Azimuth and known canonical marker genes (S1 Table) where relevant. Related to Fig 2.

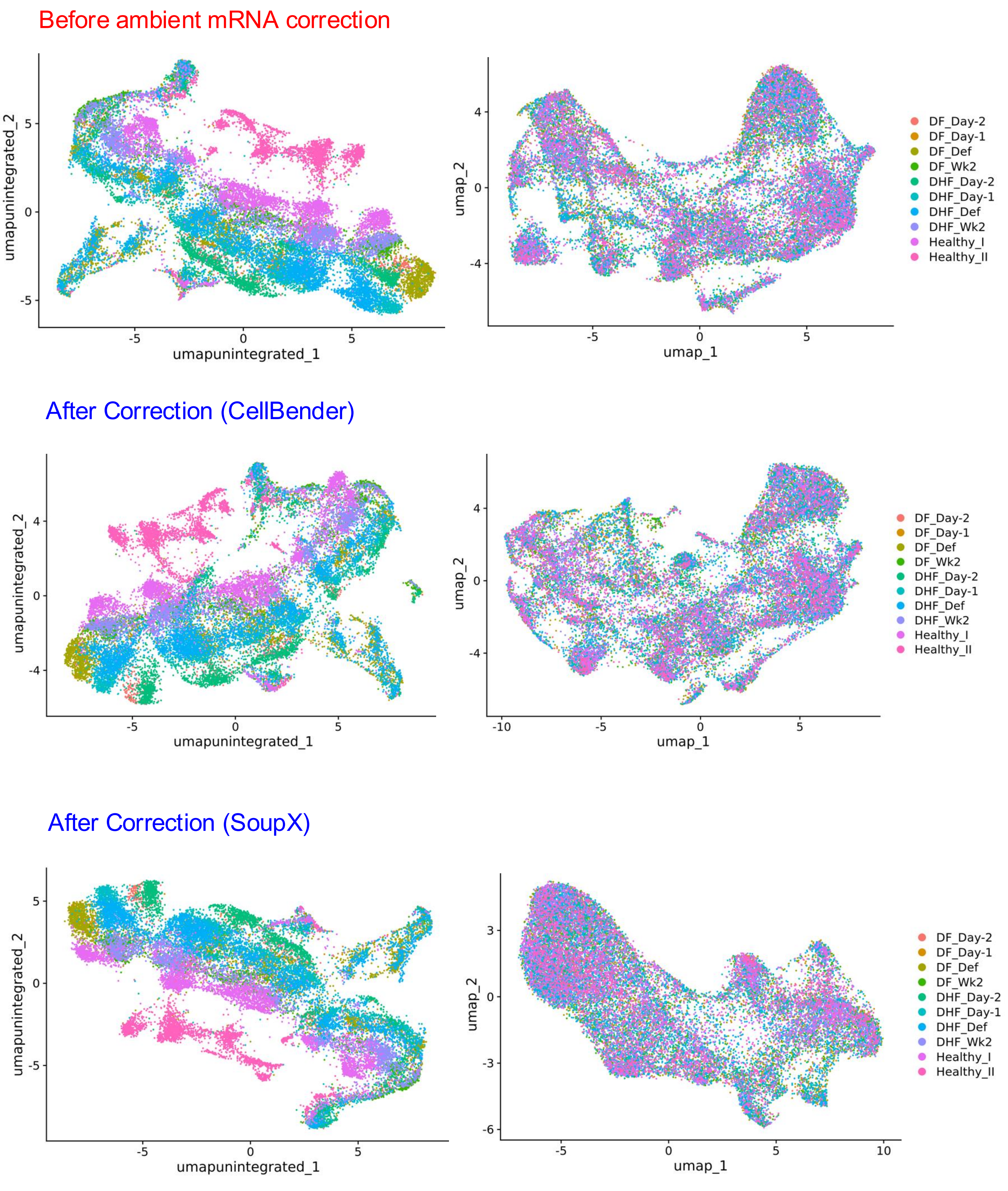

S8 Fig. UMAP plots showing T cell subsets before (left panel) and after (right panel) batch effect correction using *SCTransform v2* from Seurat V.5.2.1. Colors represent individual single-cell RNA-seq libraries. Related to Fig. 3.

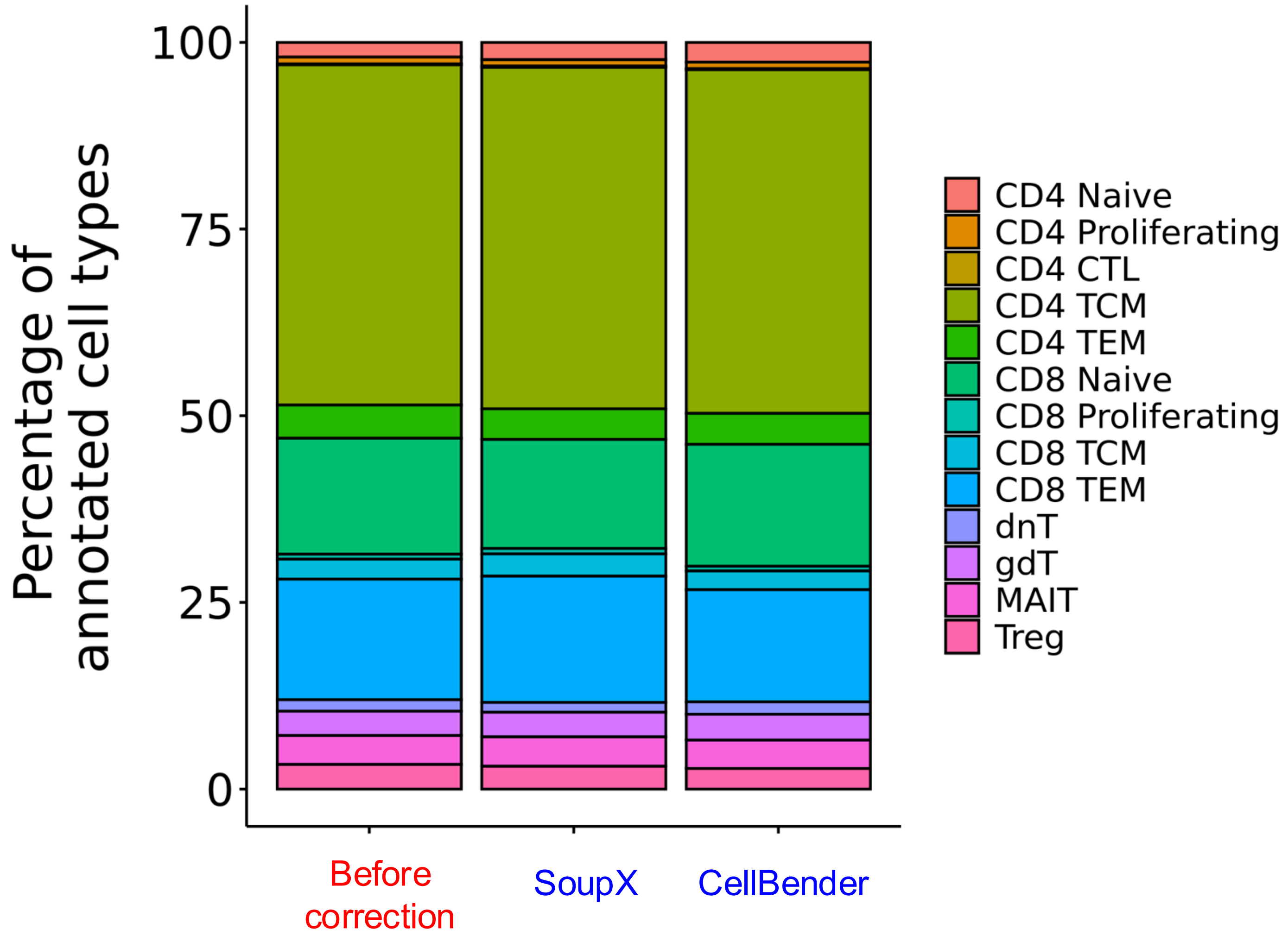

S9 Fig. Bar plot showing the percentages of annotated T cell subpopulations using Azimuth, supplemented by well-characterised marker genes before and after correction. Colors represent different cell types. Related to Fig 3.

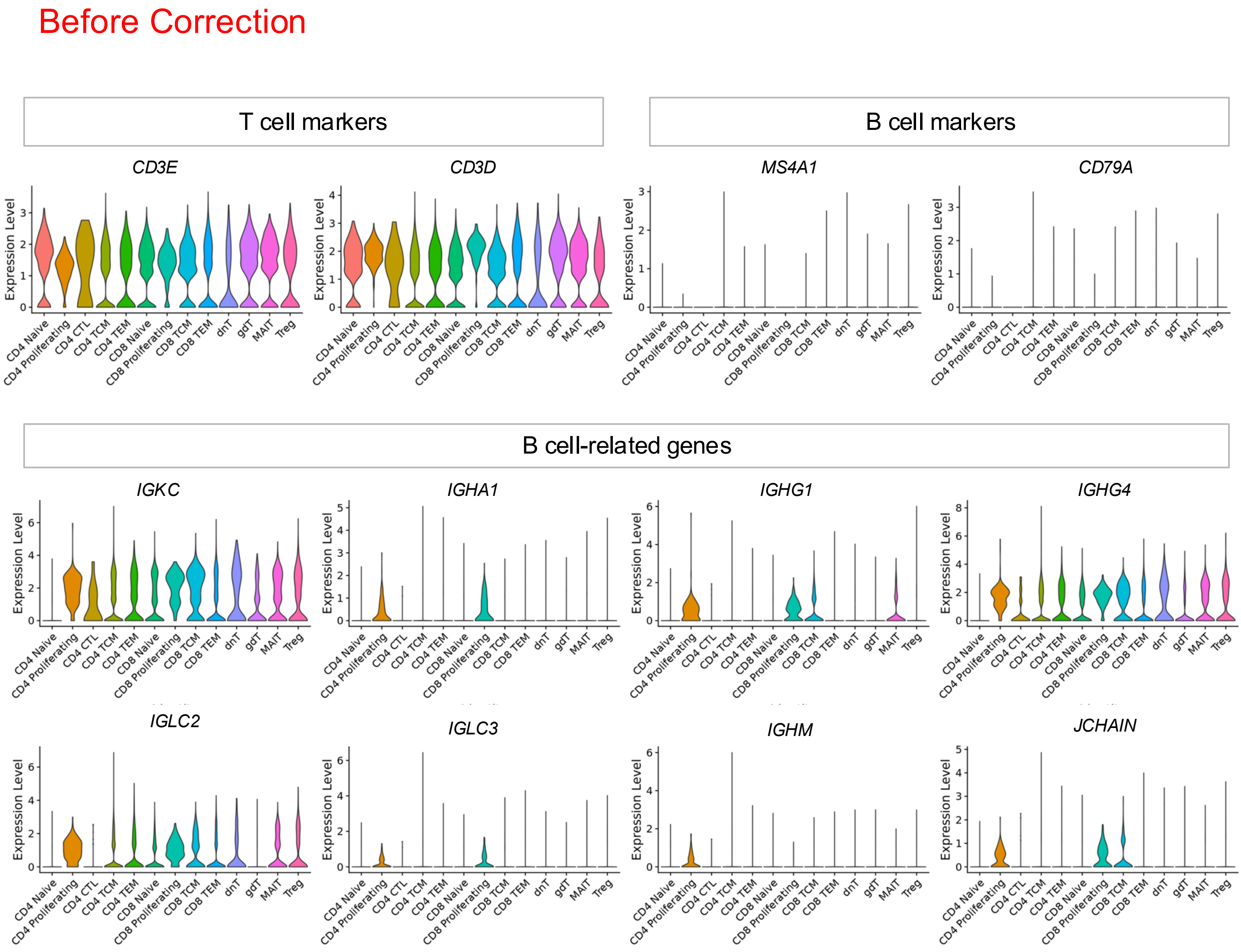

S10 Fig. Violin plots showing the normalised expression levels of T cell, B cell, and B cell-related genes in integrated T cell subsets (annotated using Azimuth) from 10 samples before ambient mRNA correction. Related to Fig 3.

After Correction (CellBender)

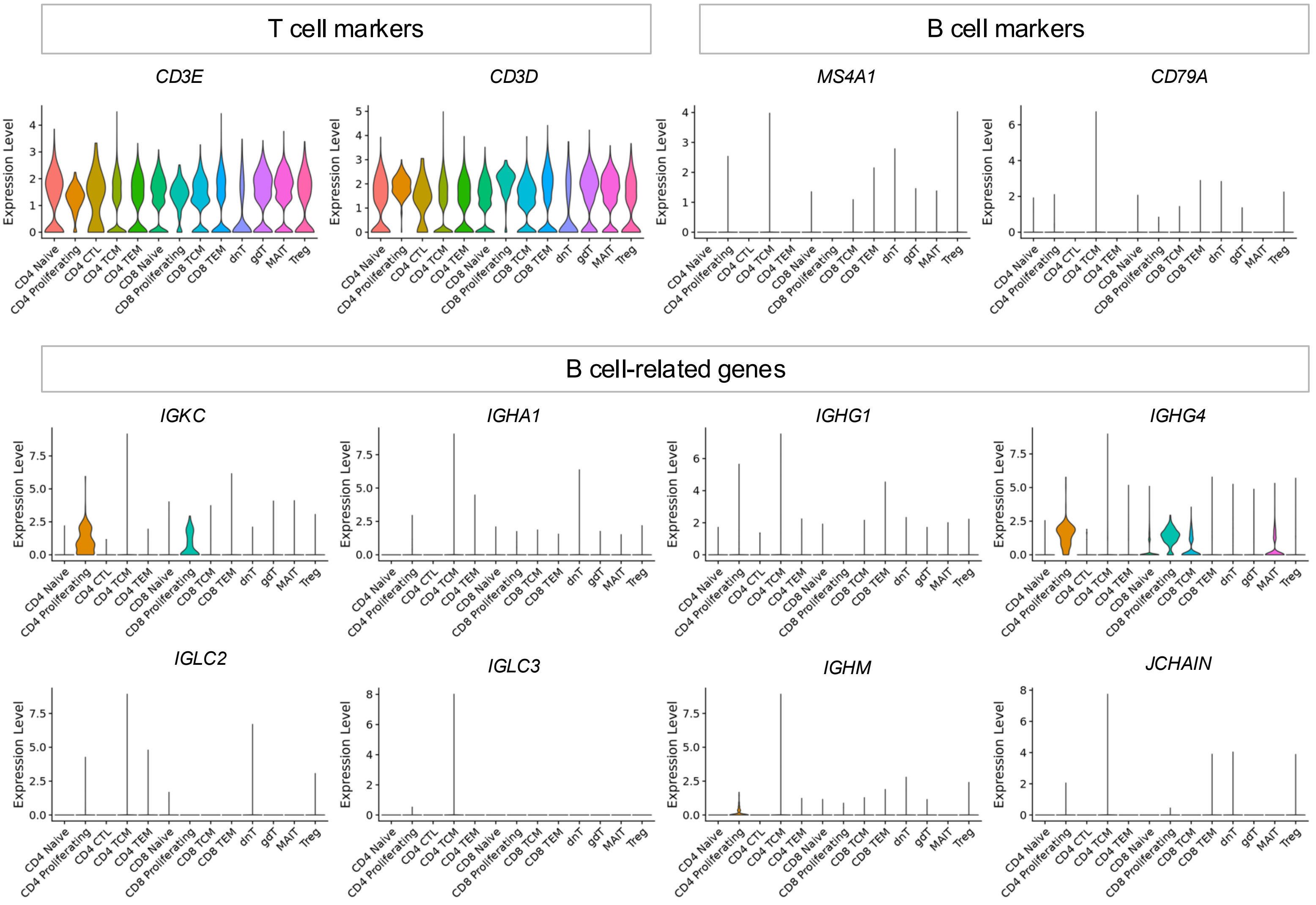

S11 Fig. Violin plots showing the normalised expression levels of T cell, B cell, and B cell-related genes in integrated T cell subsets (annotated using Azimuth) from 10 samples after ambient mRNA contaminations with CellBender. Related to Fig 3.

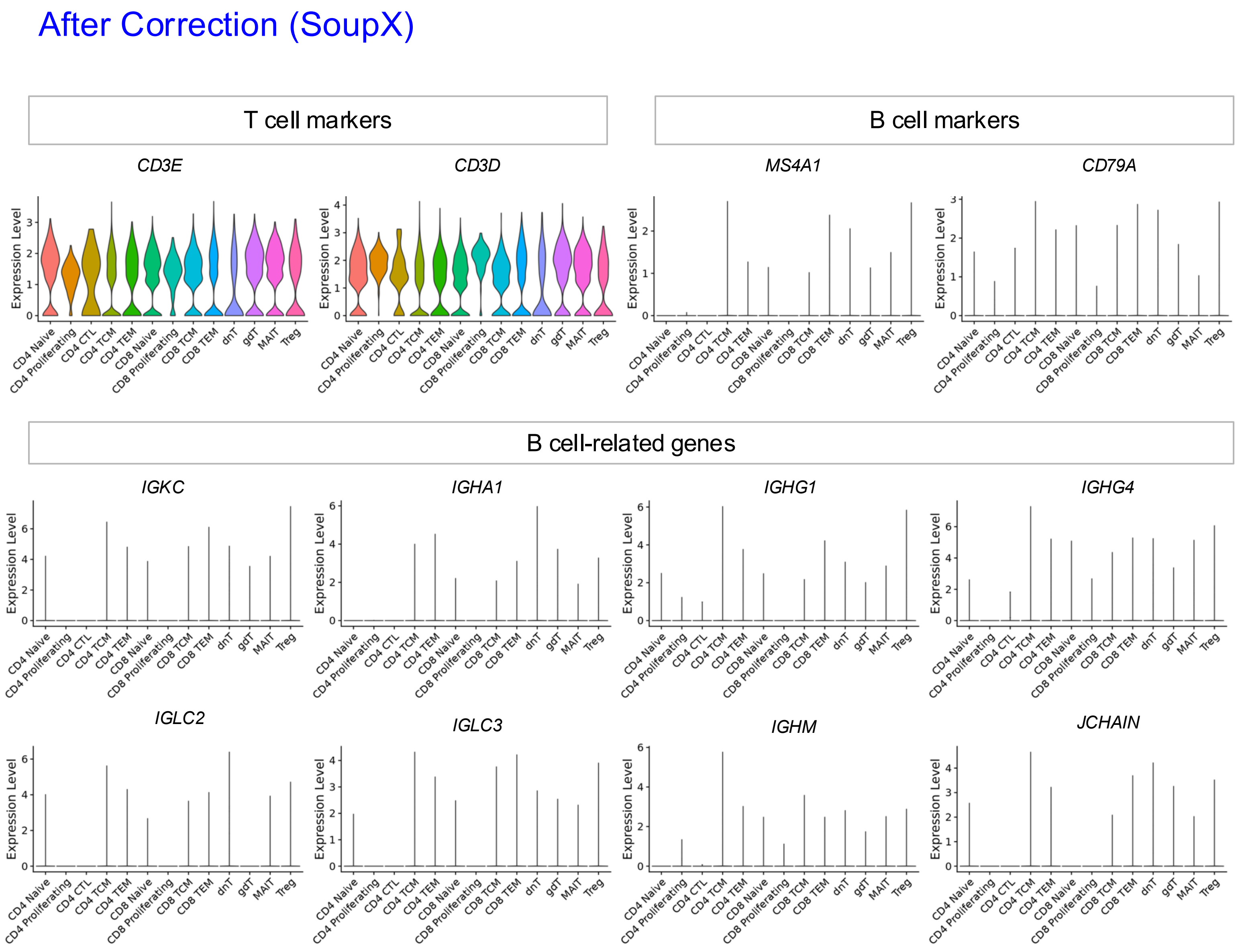

S12 Fig. Violin plots showing the normalised expression levels of T cell, B cell, and B cell-related genes in integrated T cell subsets (annotated using Azimuth) from 10 samples after ambient mRNA contaminations with SoupX. Related to Fig 3.

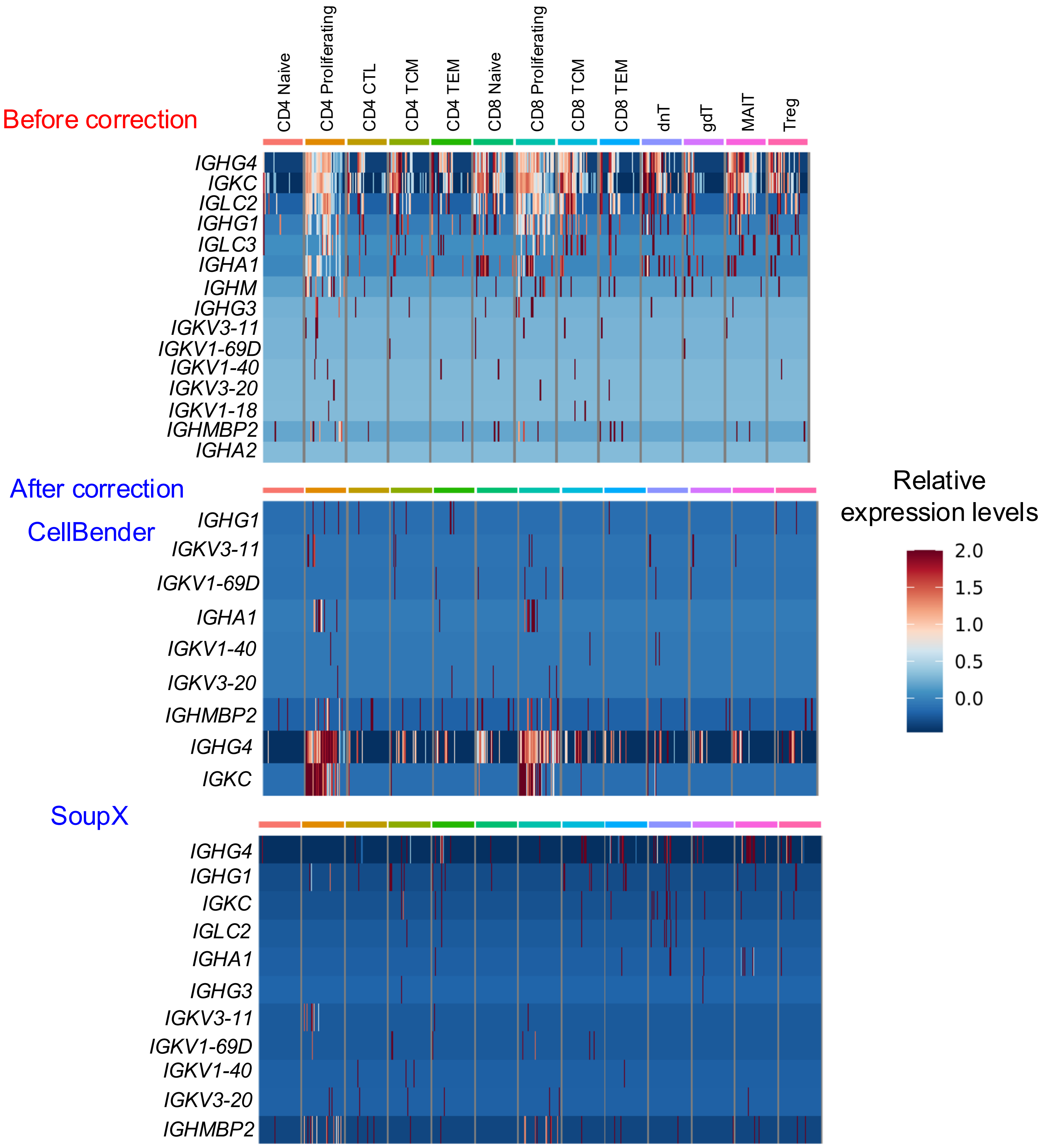

S13 Fig. Heatmap showing the relative expression levels of immunoglobulin (Ig) genes observed in the top 20 differentially expressed genes (DEGs) in at least one of the three biological conditions (acute, convalescent, or healthy control), when compared to the other two conditions, across T cell subsets annotated using Azimuth. DEGs were identified using the Wilcoxon rank sum test through the *FindAllMarker* function in Seurat V.5.2.1. Related to Fig. 3.

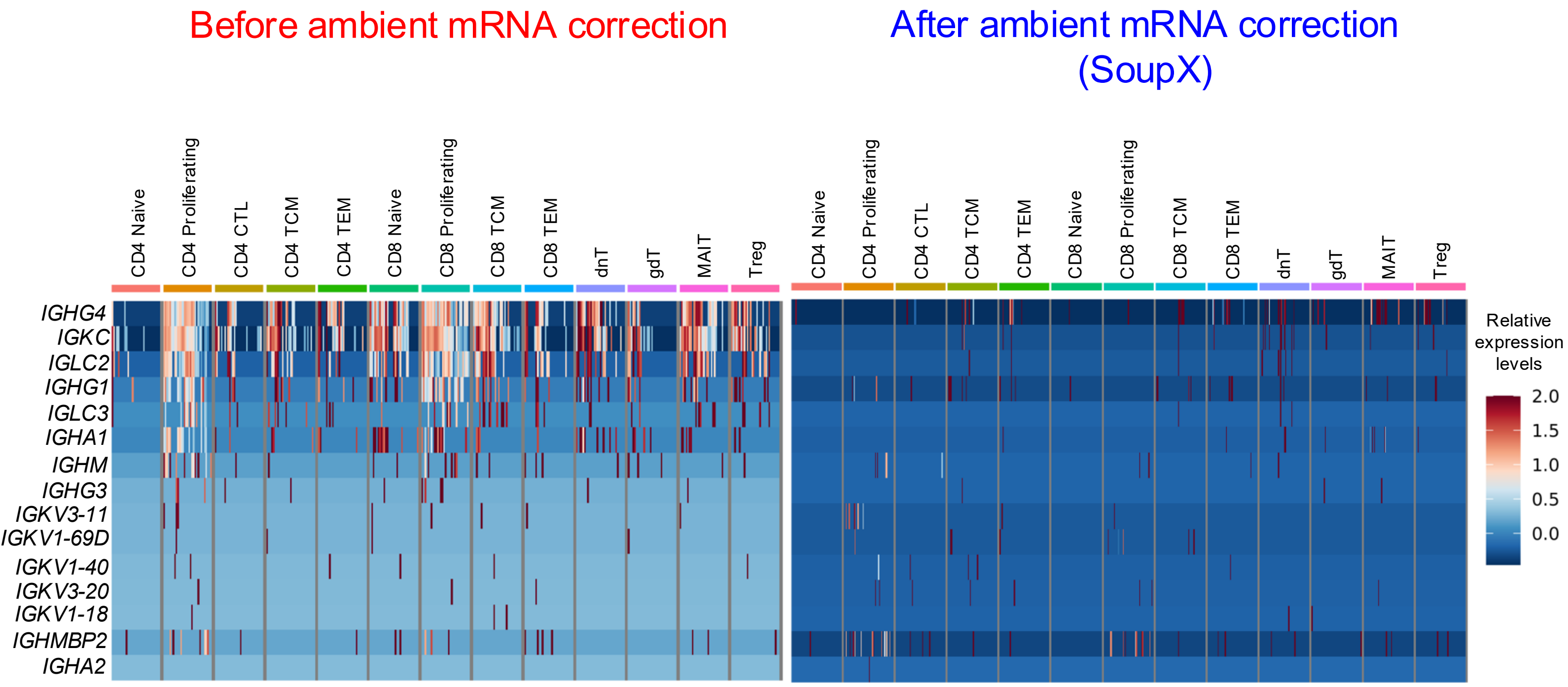

S14 Fig. Heatmap showing the relative expression levels of immunoglobulin (Ig) genes observed in the top 20 differentially expressed genes (DEGs) in at least one of the three biological conditions (acute, convalescent, or healthy control), when compared to the other two conditions, across T cell subsets annotated using Azimuth. DEGs were identified using the Wilcoxon rank sum test through the *FindAllMarker* function in Seurat V.5.2.1. Related to Fig. 3.

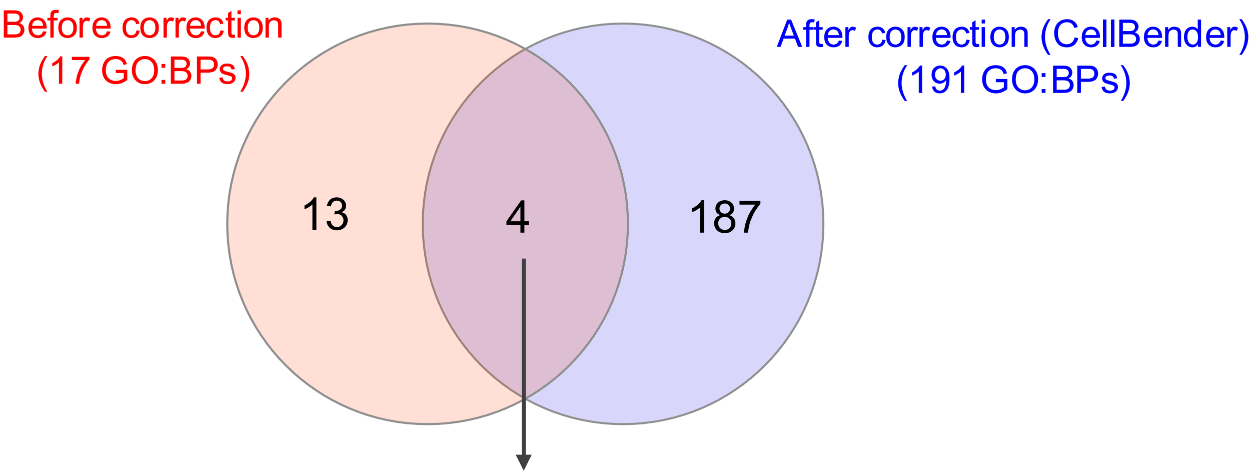

Term ID and name	Intersection genes	
	Before correction	After correction CellBender
GO:0050896 response to stimulus	*IGHG4, IGLC2, IGHG1, JCHAIN, IGLC3, MZB1, IFI27, IGKV3-11, IGHV1-69D, TNFRSF17, IGLV1-40, APOBEC3B, IGKV3-20, IL10, IGHV1-18, SGCD, MYO6, GIPR, DTHD1, MYOCD, EFNA5, NME8, ARHGEF28, LYZ, SCGB3A1, PPP1R2C, S100A12, RETN, SERPINA1*	*ISG15, IFI44L, XAF1, IFIT3, IFI27, CDT1, IFIT2, IGHV1-69D, RSAD2, FCER1G, E2F1, CDC45, IRF8, MCM10, CDC20, IGLV1-40, IGKV3-20, PTTG1, ZWINT, MYBL2, CDCA5, CDK1, CDC6, CLSPN, UBE2T, CCNB1, ITM2C, SPC25, SCGB3A1, TRHDE, PPP1R2C*
GO:0098542 defense response to other organism	*IGHG4, IGHG1, JCHAIN, IFI27, APOBEC3B, IGKV3-20, IL10, LYZ, S100A12*	*ISG15, IFI44L, IFIT3, IFI27, IFIT2, RSAD2, FCER1G, IRF8, IGKV3-20*
GO:0006955 immune response	*IGHG4, IGLC2, IGHG1, JCHAIN, IGLC3, IFI27, IGKV3-11, IGHV1-69D, TNFRSF17, IGLV1-40, APOBEC3B, IGKV3-20, IL10, IGHV1-18, LYZ, S100A12*	*ISG15, IFI44L, IFIT3, IFI27, IFIT2, IGHV1-69D, RSAD2, FCER1G, IRF8, IGLV1-40, IGKV3-20*
GO:0042742 defense response to bacterium	*IGHG4, IGHG1, JCHAIN, IGKV3-20, IL10, LYZ, S100A12*	*ISG15, FCER1G, IRF8, IGKV3-20*

S15 Fig. Venn diagram representing the number of unique and shared Gene Ontology Biological Process (GO:BP) pathways before and after ambient mRNA correction with CellBender. Related to Fig. 3B.

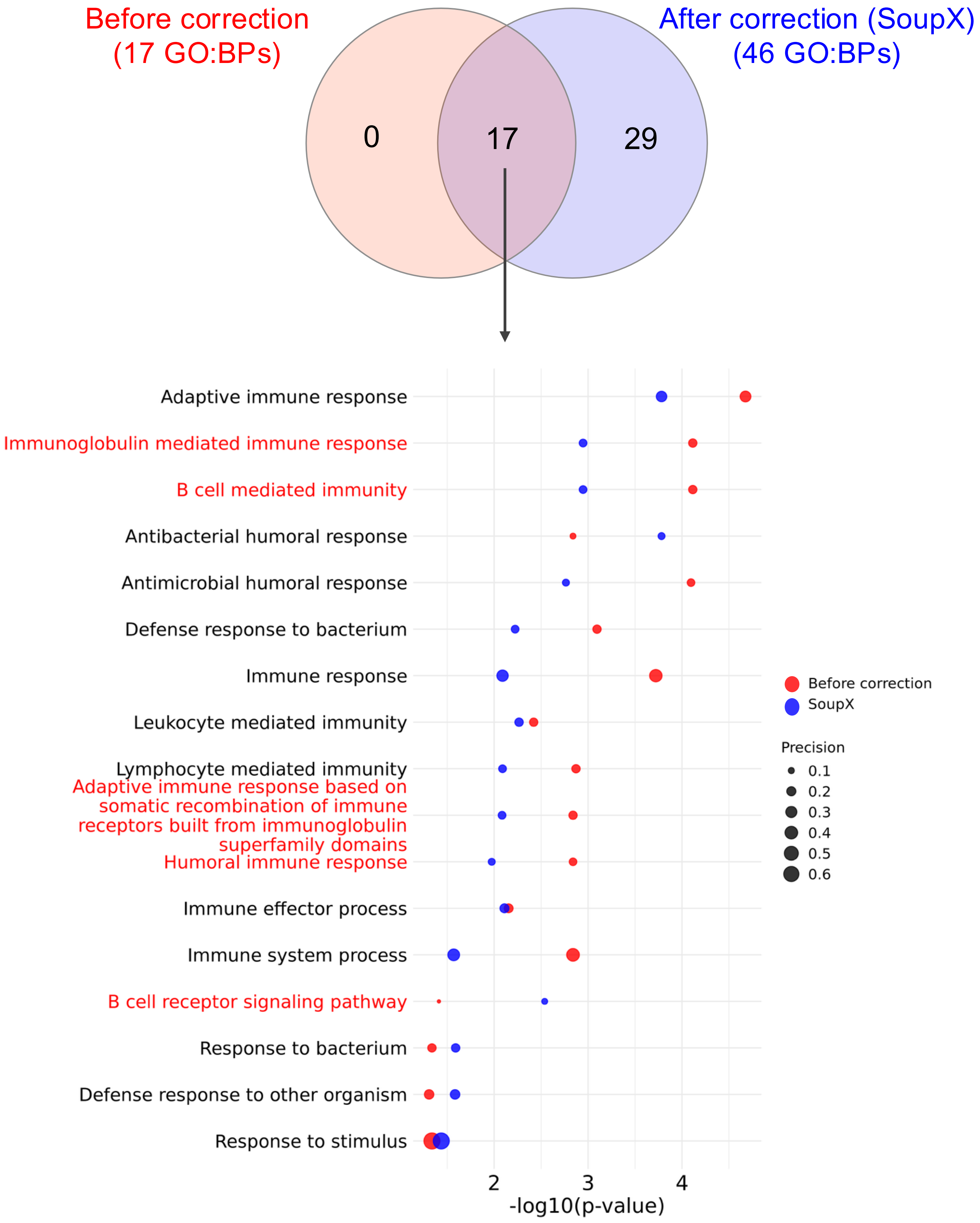

S16 Fig. Venn diagram (top panel) representing the unique and shared Gene Ontology Biological Process (GO:BP) pathways before and after ambient mRNA correction using SoupX. The bottom panel shows the 17 significant GO:BP pathways shared between before and after correction. Red-labeled pathways are B cell-specific pathways that have been previously reported in dengue infection study [13]. Related to Fig. 3B.

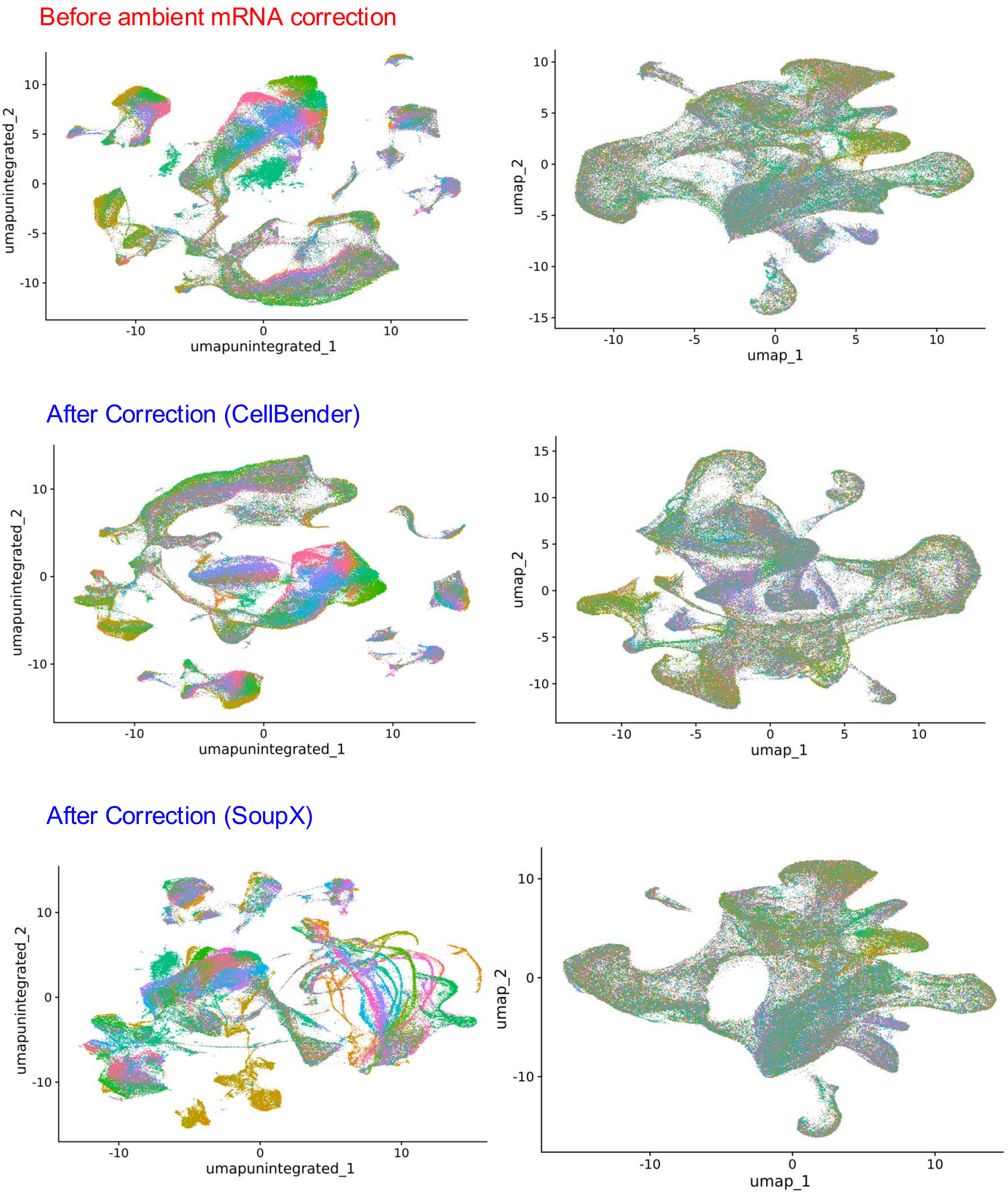

S17 Fig. UMAP plots of forty-two integrated samples before (left panel) and after (right panel) batch effect correction using *SCTransform v2* from Seurat V.5.2.1. Colors represent individual single-cell RNA-seq libraries. Related to Fig. 4.

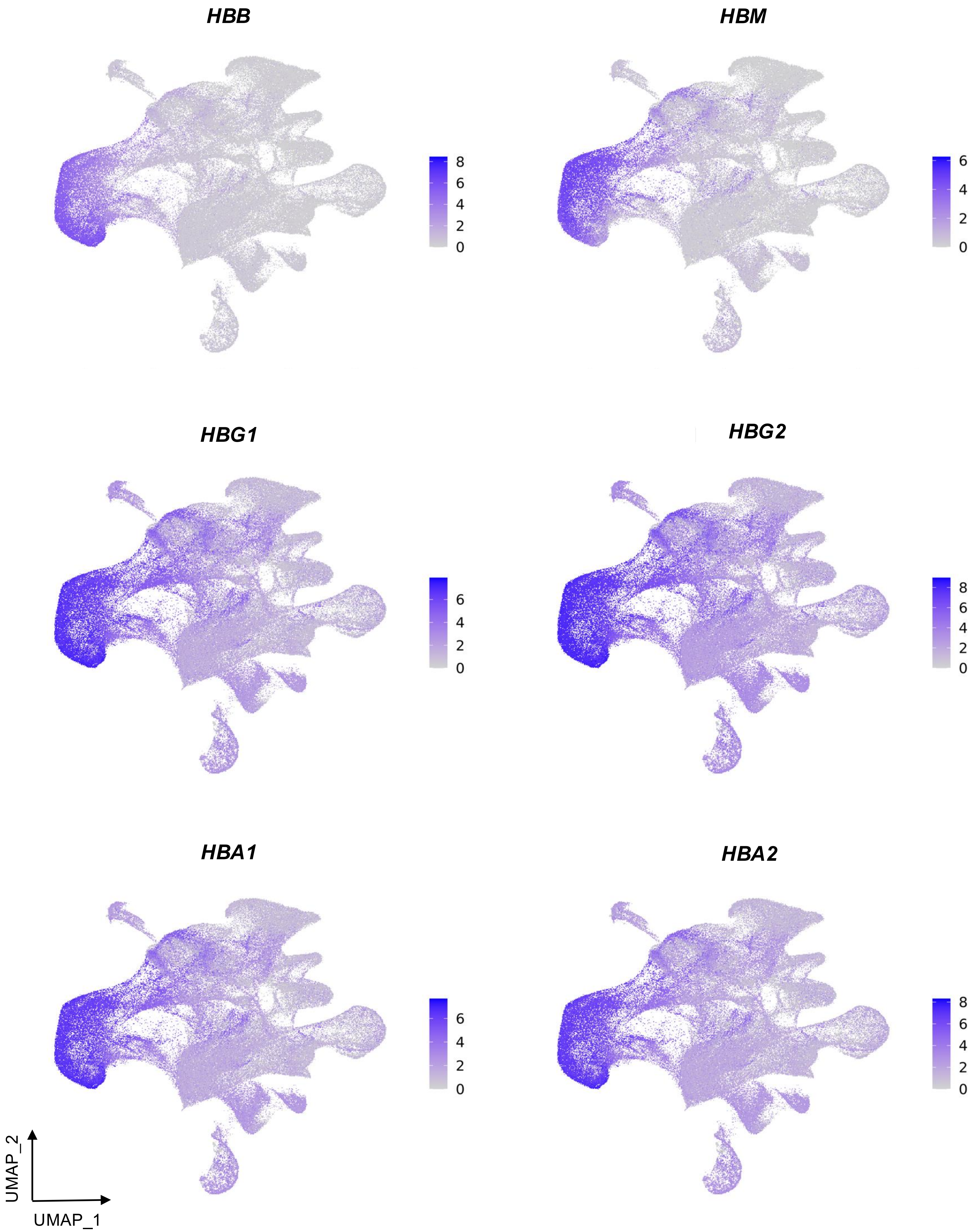

S18 Fig. UMAP feature plots showing the expression levels of hemoglobin genes in integrated forty-two human fetal liver tissues. Related to Fig 4.

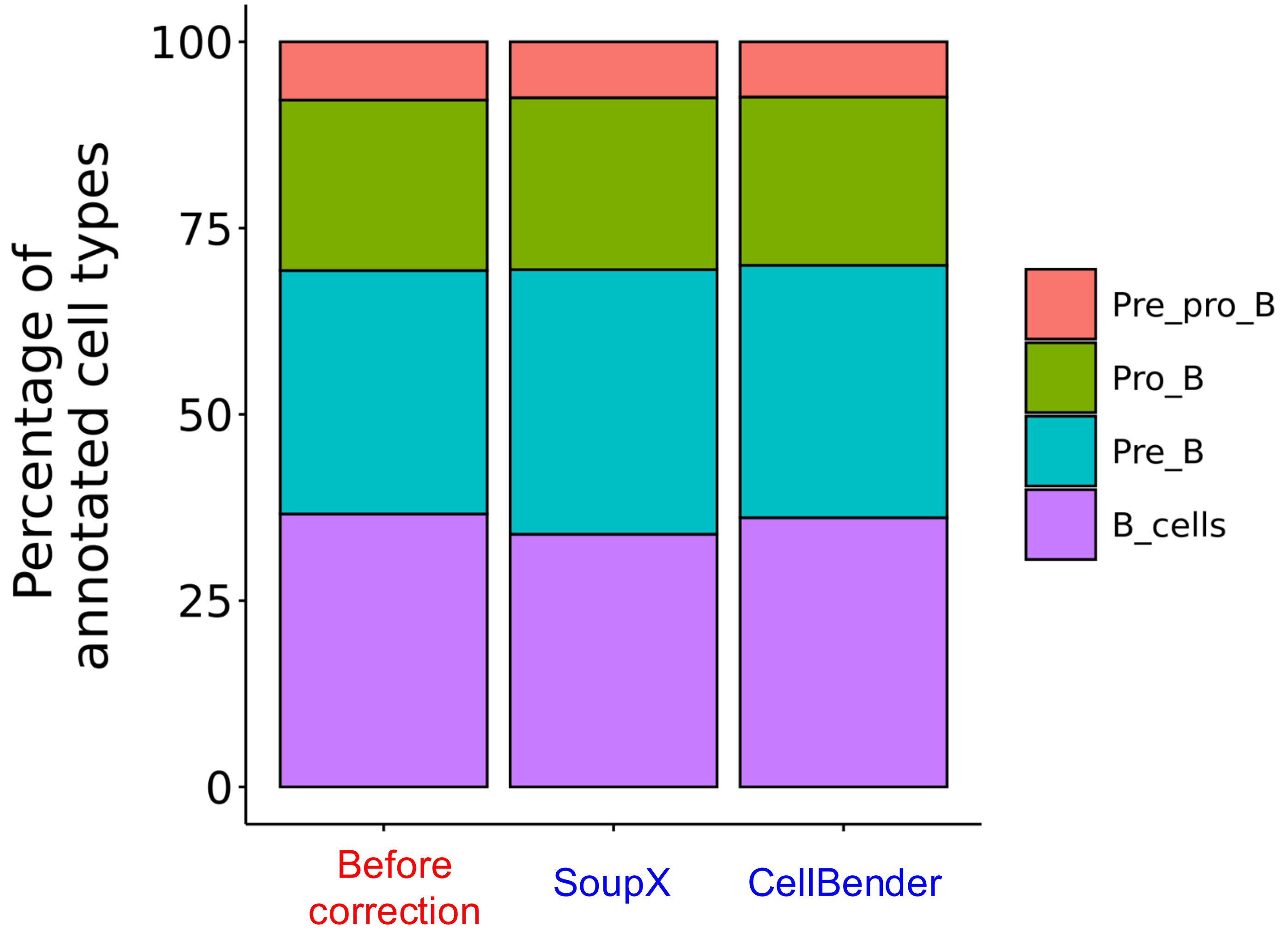

S19 Fig. Bar plot showing the percentages of annotated B cell subpopulations using Azimuth, and the original article's annotation [21]. Colors represent different cell types. Related to Fig 4.

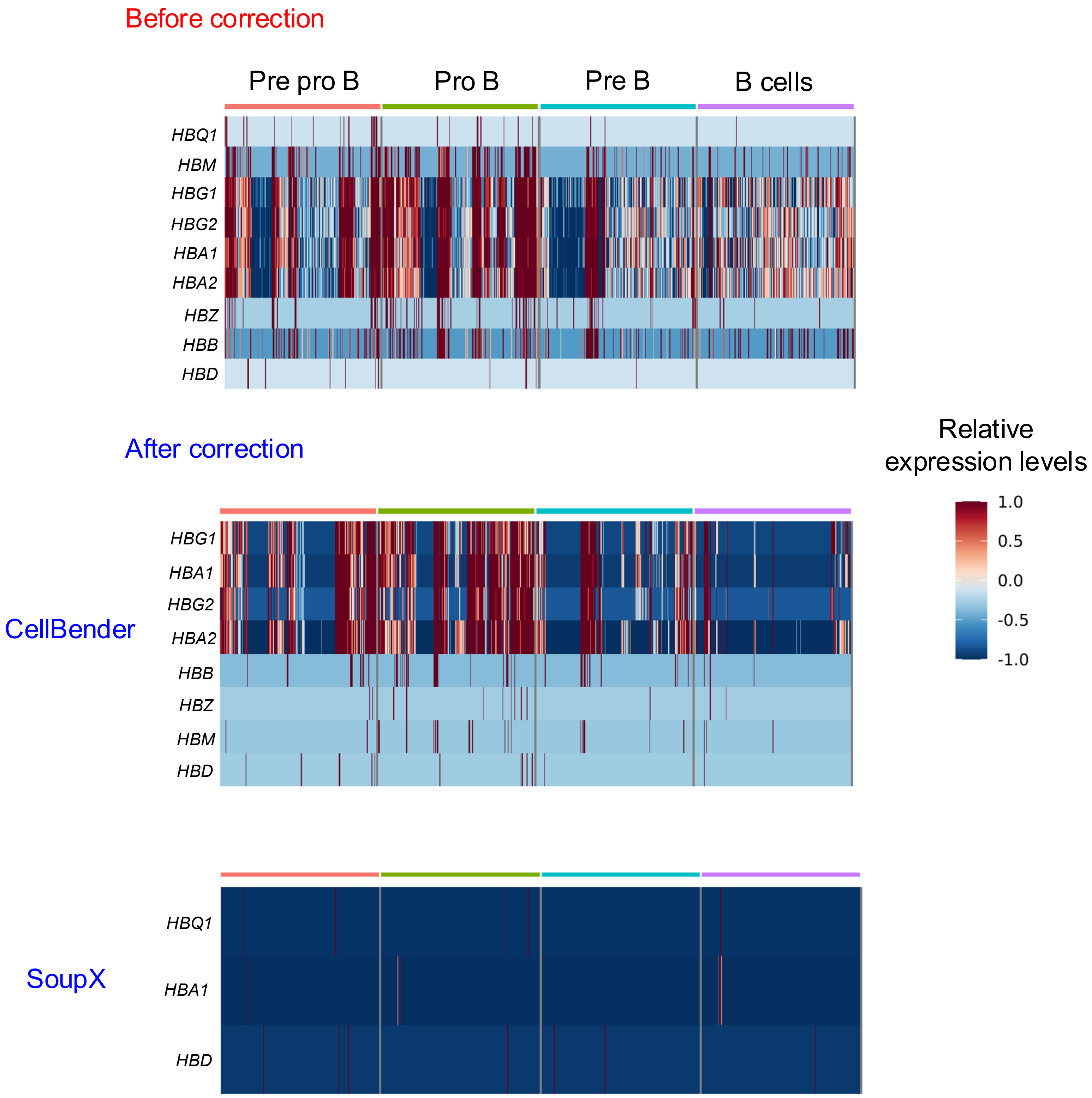

S20 Fig. Heatmap demonstrating the relative expression levels of hemoglobin (Hb) genes observed in the differentially expressed gene (DEGs) between each B cell subsets, compared to the rest. DEGs were identified using the Wilcoxon rank sum test through the *FindAllMarker* function in Seurat V.5.2.1. Related to Fig 4.

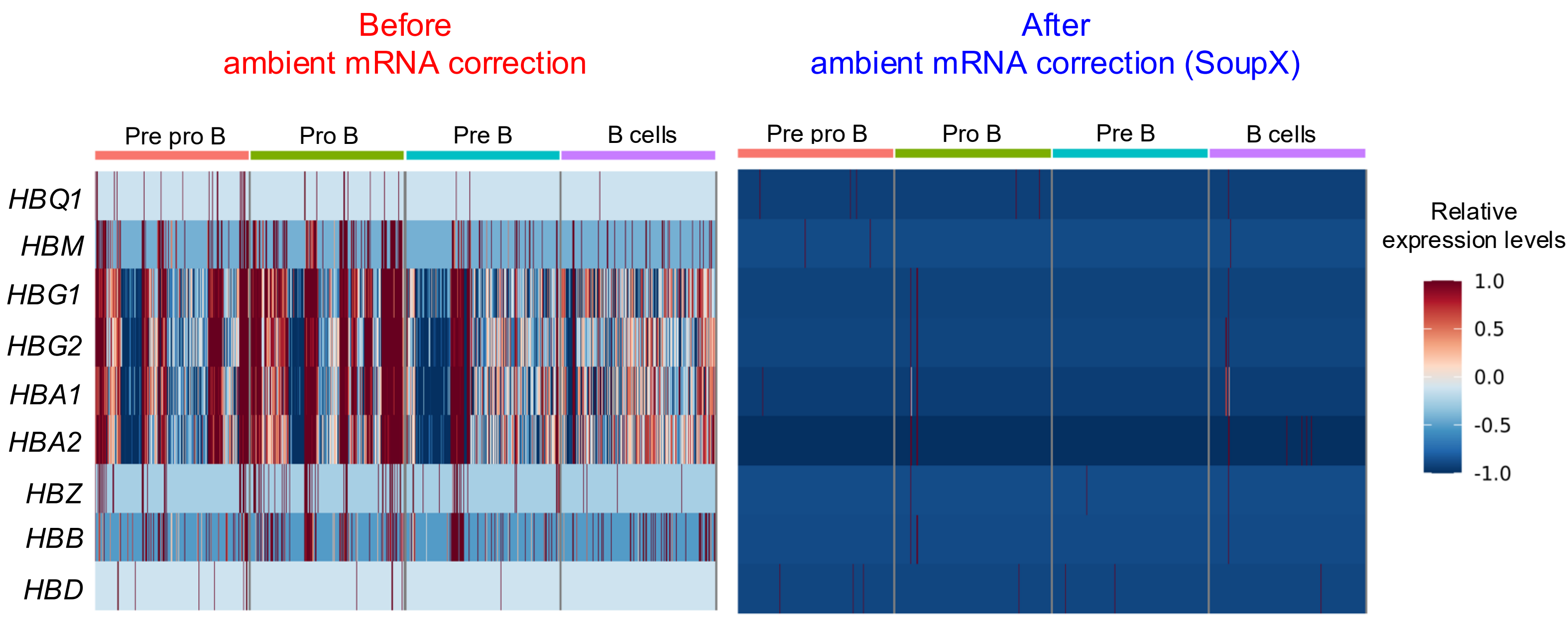

S21 Fig. Heatmap showing the relative expression levels of hemoglobin (Hb) genes among differentially expressed genes (DEGs) across B cell subsets, as annotated in the original paper (Popescu et al., 2019), before (left panel) and after (right panel) ambient mRNA correction using SoupX. DEGs were identified using the Wilcoxon rank sum test through the *FindAllMarker* function in Seurat V.5.2.1. Related to Fig 4.

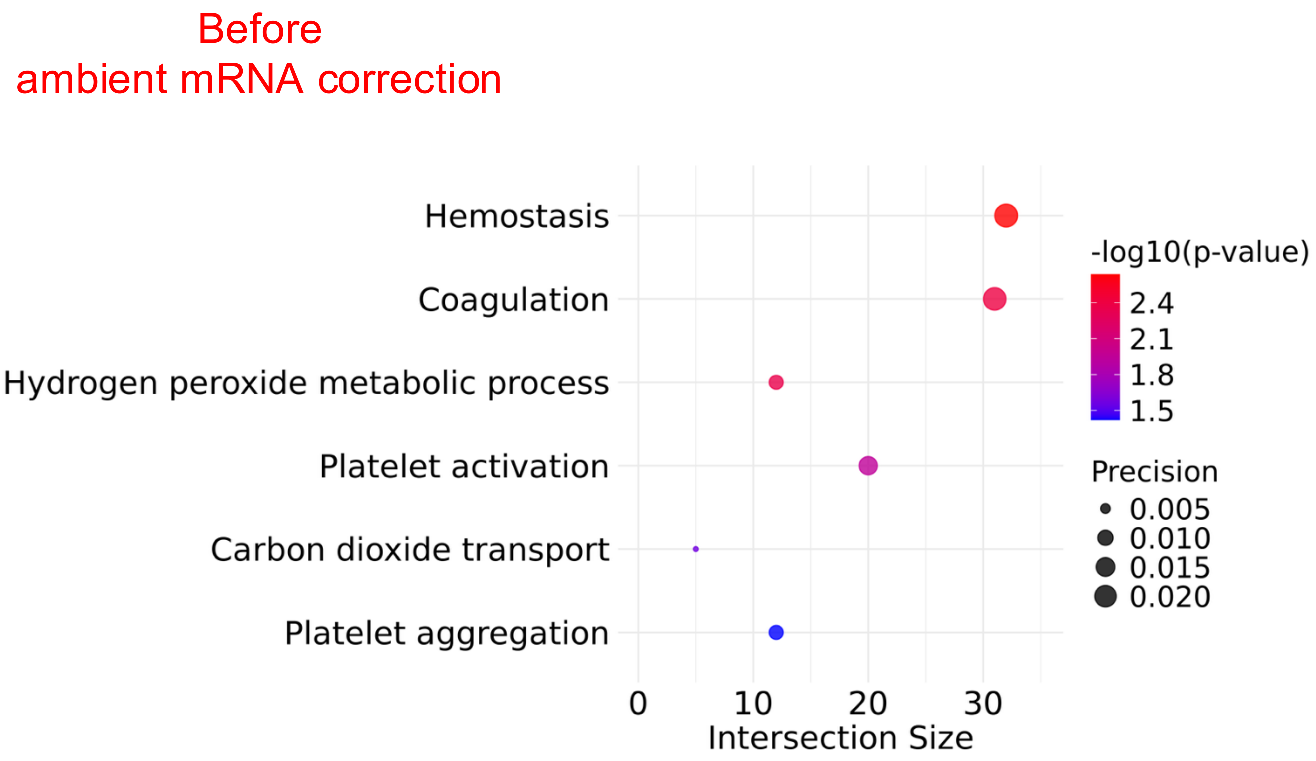

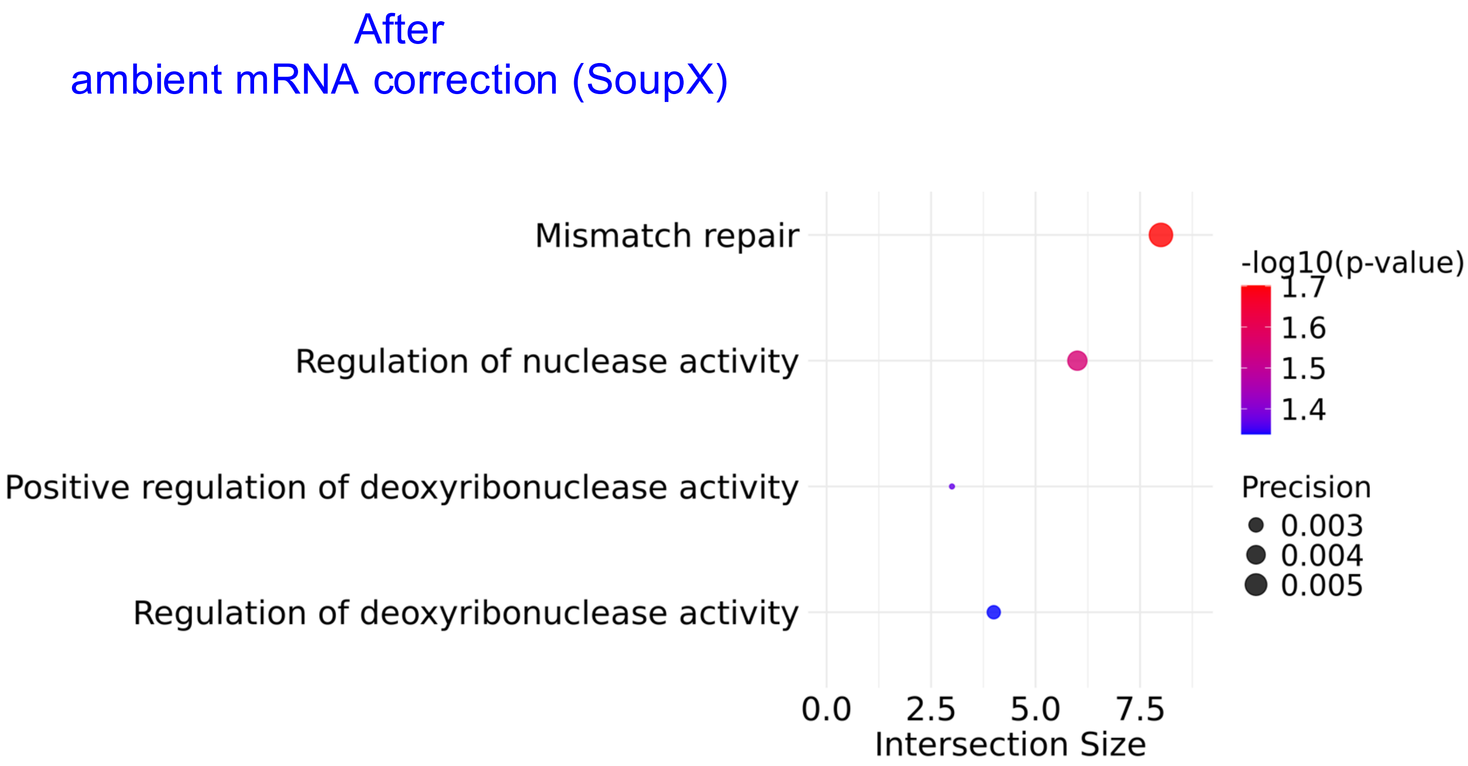

S22 Fig. The unique significant GO:BP pathways that were observed only before correction and are associated with Hb genes (upper panel). The bottom panel represents the significant GO:BP pathways that were found only after correction. Related to Fig. 4

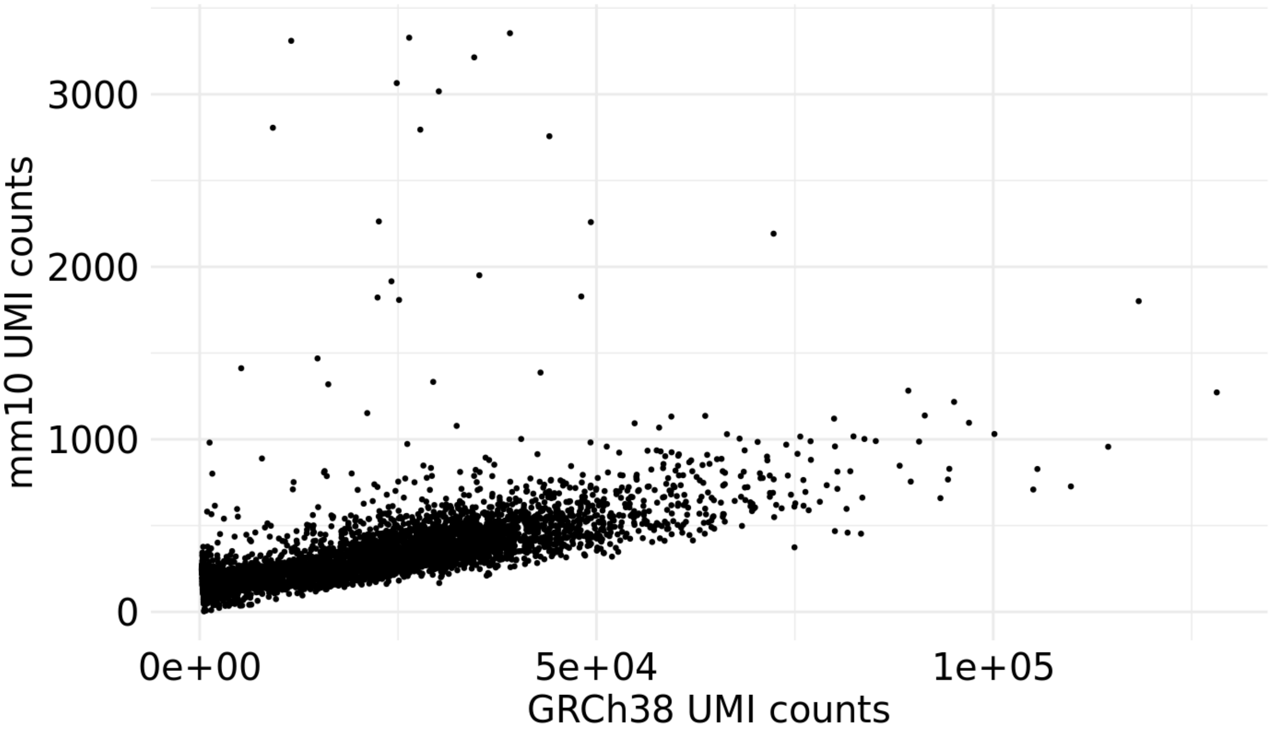

S23 Fig. Unique UMI counts per droplet that align to human (GRCh38) and mouse (mm10) gene references. Each droplet represents a human cell from a species-mixing dataset (10x Genomics; https://www.10xgenomics.com/datasets/10-k-1-1-mixture-of-human-hek-293-t-and-mouse-nih-3-t-3-cells-3-v-3-1-3-1-standard-6-0-0, retrieved on 20 June 2025), showing a low level of cross-species contamination.

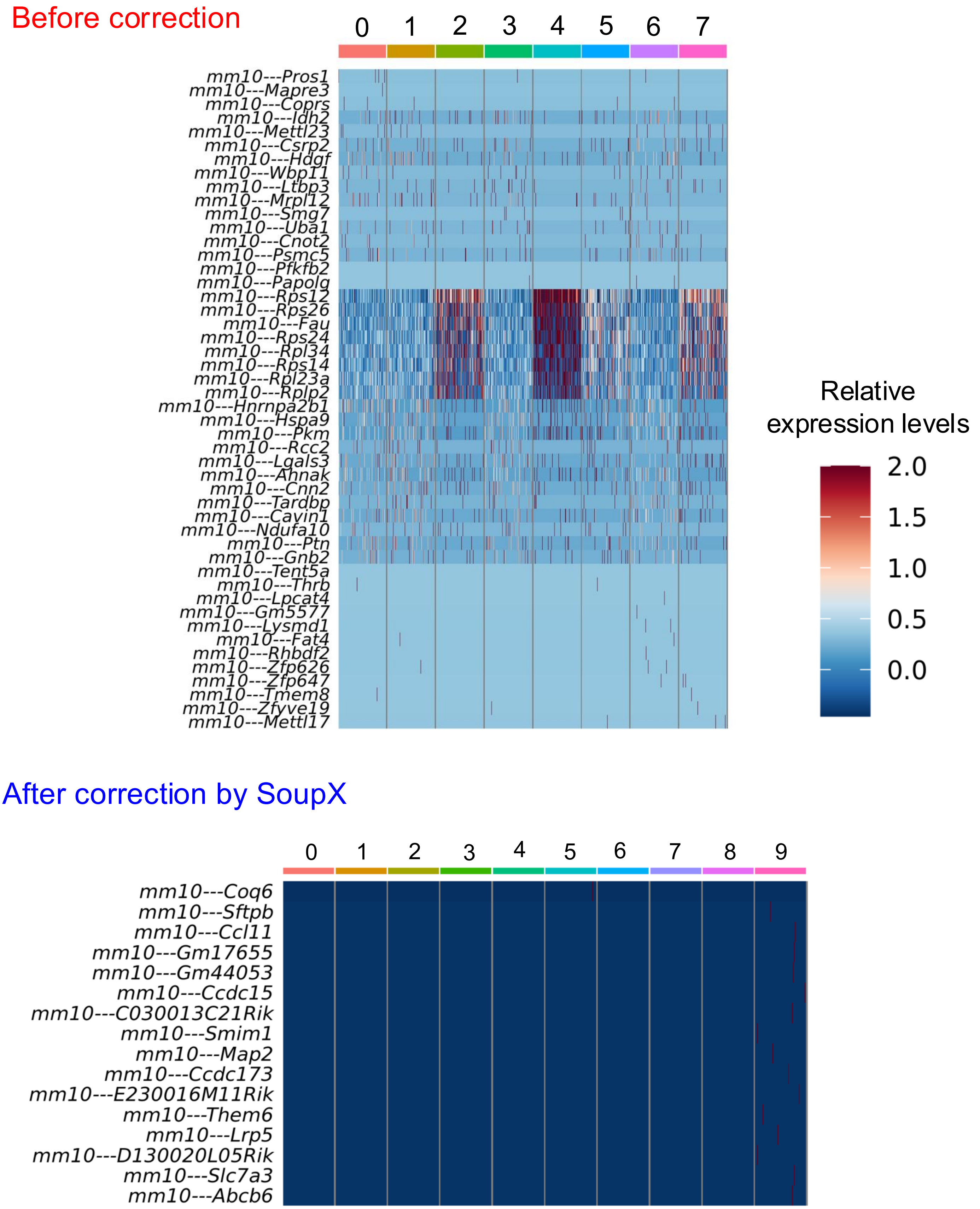

S24 Fig. Heatmap showing the relative expression levels of mouse (mm10--) genes among the top 20 differentially expressed genes (DEGs) across clusters in the human dataset from a species-mixing experiment (10x Genomics; https://www.10xgenomics.com/datasets/10-k-1-1-mixture-of-human-hek-293-t-and-mouse-nih-3-t-3-cells-3-v-3-1-3-1-standard-6-0-0, retrieved on 20 June 2025). DEGs were identified using the Wilcoxon rank sum test via the *FindAllMarker* function in Seurat V.5.2.1. Related to S21 Fig.

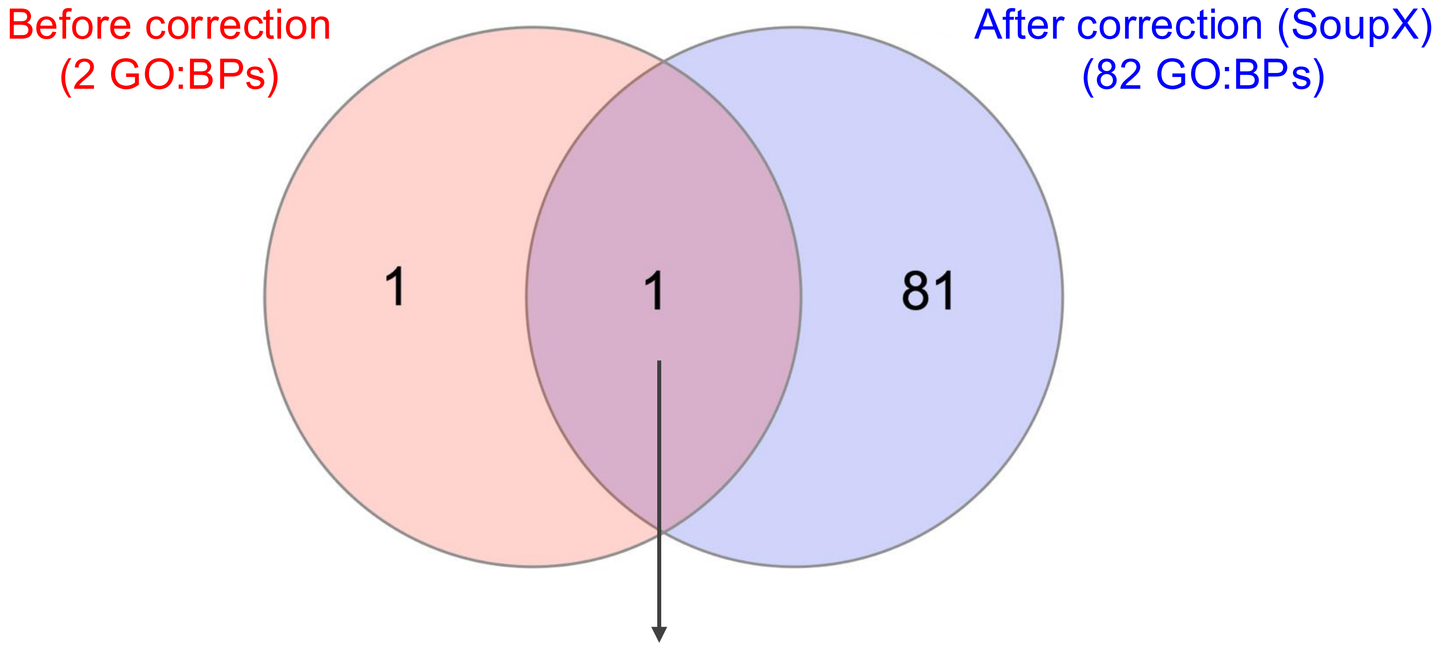

Term ID and name	Intersection genes	
	Before correction	After correction SoupX
GO:0002181 cytoplasmic translation	*mm10---Rps12, mm10---Rps26, mm10---Fau, mm10---Rps24, mm10---Rpl34, mm10---Rps14, mm10---Rpl23a, mm10---Rplp2, mm10---Pkm*	*GRCh38-RPS28, GRCh38-RPS12, GRCh38-RPL15, GRCh38-RPL36, GRCh38-RPL13, GRCh38-RPS10*

S25 Fig. Venn diagram showing the number of unique and shared Gene Ontology Biological Process (GO:BP) pathways identified before and after ambient mRNA correction with SoupX in the human dataset from a species-mixing experiment (10x Genomics; https://www.10xgenomics.com/datasets/10-k-1-1-mixture-of-human-hek-293-t-and-mouse-nih-3-t-3-cells-3-v-3-1-3-1-standard-6-0-0, retrieved on 20 June 2025). Related to S21-S22 Figs.
