# Supplementary material for: Understanding and mitigating the impact of ambient mRNA contamination in single-cell RNA-sequencing analysis
Source: PLoS One. 2025 Sep 24;20(9):e0332440. doi: 10.1371/journal.pone.0332440 (PMC12459771; doi:10.1371/journal.pone.0332440)
Supplement: Attachment — Submitted filename: Response to Reviewers.pdf [file pone.0332440.s023.pdf]

Dear Academic Editor and Reviewers,

Thank you for your comments and for the opportunity to revise our manuscript. We have addressed the following.

1. Please ensure that your manuscript meets PLOS ONE's style requirements, including those for file naming.

We have now rechecked and formatted our revised manuscript to comply with PLOS ONE's style requirements.

2. Thank you for stating the following financial disclosure:

"This project is funded by the mid-career researcher grant from National Research Council of Thailand (NRCT) and Mahidol University (N42A670557) through VC."

Please state what role the funders took in the study. If the funders had no role, please state: "The funders had no role in study design, data collection and analysis, decision to publish, or preparation of the manuscript." If this statement is not correct you must amend it as needed. Please include this amended Role of Funder statement in your cover letter; we will change the online submission form on your behalf.

We confirm that the grant funder had no role in study design or any influence on manuscript preparation. We have added the sentence as suggested; please refer to page 15, line 447.

Page 15, Line 447:

"This project is funded by the mid-career researcher grant from National Research Council of Thailand (NRCT) and Mahidol University (N42A670557) through VC. The funders had no role in study design, data collection and analysis, decision to publish, or preparation of the manuscript."

3. We noted in your submission details that a portion of your manuscript may have been presented or published elsewhere.

[Single-cell RNA-seq datasets of dengue patients and one healthy donor were available through the ArrayExpress repository under accession number E-MTAB-9467. Additionally, one healthy sample was obtained from the 10x Genomics website (4k PBMCs from a healthy donor, Single Cell Gene Expression Dataset by Cell Ranger 2.1.0, 10x Genomics, 2017, November 8, https://support.10xgenomics.com/single-cell-gene-expression/datasets/2.1.0/pbmc4k). The raw and filtered gene-barcode matrices of the ten samples were obtained from: https://data.mendeley.com/datasets/6ry3x7r8hf/3. The raw and filtered gene-barcode matrices of forty single-cell experiments from human fetal liver tissues were deposited in the GigaDB repository, available at: http://gigadb.org/dataset/100836#.]

Please clarify whether this [conference proceeding or publication] was peer-reviewed and formally published. If this work was previously peer-reviewed and published, in the cover letter please provide the reason that this work does not constitute dual publication and should be included in the current manuscript.

We would like to clarify that our manuscript is an original work and does not constitute dual publication. In this study, we have specifically addressed the influences of ambient RNAs on downstream analyses of scRNA-seq datasets – a topic that was not the focus of the original publicly available publications we utilised. These datasets, including those published by us as well as others, served as sources for raw data used to demonstrate the impact of ambient RNAs in the scRNA-seq results. As described more explicitly now in the manuscript, we re-processed the raw data using different analytical methods and pipelines from the original studies to investigate the effects of ambient RNAs contamination. This approach allowed us to generate new insights that were not part of the original publications.

Summary of new analyses added during this revision

We appreciate both reviewers and the editor for the insightful comments and suggestions. Here, we would like to give a brief summary of new analyses added to the revised work, in response to your insightful comments.

In the original manuscript, we mentioned an improvement in clustering efficiency, particularly in the separation of B cell subpopulations after ambient RNA correction. In response to the reviewers' comments regarding the qualitative nature of UMAP representations and the lack of quantitative measures to assess clustering quality in an unbiased manner in the previous version, we agree that visual interpretation of UMAP plots is insufficient for drawing robust conclusions, and hence now replaced it with new analyses described below.

In this revised manuscript, we have introduced several methodological updates, including:

- Upgrading from Seurat V4 to V5 – which can impact clustering results in several ways, as Seurat V5 introduces updates and improvements in data processing, normalisation, and clustering algorithms. Specifically, *SCTransform* has been updated to *SCTransform v2* which improves variance stabilisation and reduces biases (Satija Lab, Seurat V5 https://satijalab.org/seurat/articles/announcements.html; Saket Choudhary & Rahul Satija, 2022 https://genomebiology.biomedcentral.com/articles/10.1186/s13059-021-02584-9). This update may affect the identification of highly variable genes, which potentially influenced principal component analysis (PCA) and downstream clustering results.
- Using Azimuth for automated cell type annotation instead of manual marker-based identification - automated annotation may have resulted in different cell type assignments, potentially affecting B cell subpopulation identification.

We also acknowledge that the direct impact of ambient RNA contamination on clustering is complex and may not always be evident in UMAP-based visualizations. Since clustering efficiency itself is difficult to quantify without robust statistical metrics, as suggested by both reviewers, and given the high sensitivity of clustering results to methodological choices, we have decided to exclude the clustering analysis from the revised manuscript.

Summary of major modifications made to the revised manuscript.

1. As suggested by both reviewers, we have incorporated CellBender (Stephen J. Fleming et al., 2023), another widely used computational tool for ambient mRNA removal, to complement our original analyses using SoupX. But please note that our primary objective is to compare gene expression profiles before and after ambient mRNA correction, rather than directly comparing the two correction methods. The two methods differ in their technical approaches: for SoupX, we provided a predefined set of potential ambient mRNA genes; whereas CellBender performs an automated prediction and correction of ambient mRNA contamination. Importantly, by applying both tools, we ensure a more comprehensive assessment of the impact of ambient mRNA contamination on downstream analyses, regardless of the technicality of the correction methods
2. We re-processed the raw FASTQ files for all datasets using the new version of the Cellranger mapping suite (v.8.0.1). This step was essential not only for generating the required input for CellBender, but also for minimising potential technical biases introduced by different mapping versions used in the original publications – v.3.0.0 for the dengue datasets (Arora et al., 2022) and v.2.0.2 for the fetal tissue data (Dorin-Mirel Popescu et al., 2019). By standardising the mapping pipeline across all datasets, we ensure greater consistency and comparability in downstream analyses.
3. We have added pathway enrichment analyses to systematically assess the biological impact of ambient mRNA contamination, before and after correction. We believe these analyses are more robust than clustering analyses alone, as they allow us to determine whether ambient mRNA removal affects key biological interpretations, providing deeper insights into its influence beyond differential gene expression.

All in all, we thank the reviewers again for their insightful comments. Please find point-by-point responses to all the comments from the reviewers below.

Best regards,

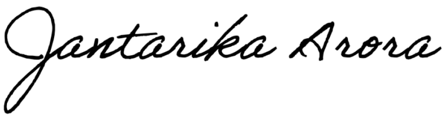

Jantarika Arora (on behalf of the authors)

Reviewers' comments:

Review Comments to the Author

Reviewer #1: The authors present a study of the effects of removal of cell-free or "ambient" RNA on downstream interpretations of scRNA-seq data. The authors used publicly-available datasets for this study. This topic is an interesting one for the field, because removal of cell-free RNA has not yet become a ubiquitous part of scRNA-seq data analysis, and it is important for studies like this to demonstrate rigorously whether or not removal of cell-free RNA impacts biological findings in an important and quantitative way.

We thank the reviewer for his/her thorough review and very constructive comments. Even more so, we are very happy to learn that he/she appreciated this important issue of ambient/cell-free RNAs that could significantly affect the downstream bioinformatic results, which is frequently overlooked.

While the topic and stated aims of the study are of great interest, the analyses as presented may not go as far as I would personally like to see. I have several recommendations to strengthen the study.

We appreciate your comprehensive comments and have tried our best to address them as follows.

Major concerns:

1. The major claim about the usefulness of removing ambient RNA is that it enhances "the accuracy of computational analyses and the robustness of data interpretation in scRNA-seq datasets." While I do agree with this statement, the results in the text focus on (1) increased log2-fold-changes of known marker genes, and (2) "notable improvement in clustering efficiency". (1) has been demonstrated by previous authors as cited in this manuscript. See the SoupX paper [15] Figure 3 for an analysis of PBMCs, which is quite similar to Figures 2 and 3 in this manuscript. And see the SoupX paper [15] Figure 4 for an analysis of kidney data, specifically showing the removal of the hemoglobin gene HBB, which is quite similar to Figure 4 in this manuscript. That is not to say that there is nothing novel here, but I am not sure that log-fold-changes of marker genes goes far enough to demonstrate the utility of ambient RNA removal.

We appreciate your valuable comments. In this revised manuscript, we have expanded our study beyond differential gene expression analysis by incorporating pathway enrichment analysis, a common goal in many single-cell studies. We have demonstrated that unexpected significant biological pathways appeared in ambient mRNA-contaminated datasets at the subpopulation levels, with a particular focus on T cell subsets (Figure 3) and B cell subsets (Figure 4), which were not shown before. Additionally, we have applied two different ambient mRNA removal approaches – CellBender (an automated correction method) and SoupX (using a predefined set of potential ambient mRNA genes) – to comprehensively assess the impact of ambient mRNA contamination, independent of the ambient removal tool/method used.

2. Major claim (2) above – "notable improvement in clustering efficiency" – may be a bit problematic. How do the authors quantify improvements in clustering efficiency? What does clustering "efficiency" mean? In Figure 4 for example, I see UMAPs before and after correction. And I see a plot of PCs before and after correction. The problem with the UMAP is that it is qualitative. The problem with the PC plot is that I am not sure whether the movement of dots on this plot is meaningful. How meaningful? How can this be quantified?

We acknowledge and agree with the reviewers' comment regarding the qualitative nature of UMAP representations and the lack of quantitative measures to assess clustering quality in an unbiased manner. We agree that relying solely on visual interpretation of UMAP plots is insufficient for drawing robust conclusions. Consequently, we have excluded the clustering analysis from the revised manuscript and instead add more quantifiable evaluations of the impact from ambient RNA contamination such as differential expressed gene and gene ontology (GO)/biological pathway assessments instead. Please see also **Summary of major modifications** at the beginning of this letter.

3. The authors state in the introduction that "the effects of ambient mRNA correction on certain downstream analyses … remains largely unclear." While I agree, the authors should probably note that other studies have been carried out which focus largely on this question, including PMID: 36240767, but there may be others.

We thank the reviewer for the suggestion. We have revised the Introduction section as follows:

Page 3, Line 68-73:

"Previous studies have also demonstrated the impact of ambient mRNA contamination on downstream analyses [16, 18]. However, there are still several aspects of ambient mRNA contaminations that remain to be characterised. Despite these advancements, the effects of ambient mRNA correction on certain downstream analyses, including differential gene expression and pathway enrichment analyses, particularly at the subpopulation levels, remains largely unclear."

16. Caglayan E, Liu Y, Konopka G. Neuronal ambient RNA contamination causes misinterpreted and masked cell types in brain single-nuclei datasets. Neuron. 2022;110(24):4043-56 e5. Epub 20221013. doi: 10.1016/j.neuron.2022.09.010. PubMed PMID: 36240767; PubMed Central PMCID: PMCPMC9789184.
18. Floriddia E. The impact of ambient RNA. Nat Neurosci. 2022;25(12):1583. doi: 10.1038/s41593-022-01232-0. PubMed PMID: 36446930.

4. The authors focused the study solely on the SoupX method. While it makes sense to limit the scope of work, it is unclear whether the conclusions would be the same if DecontX or CellBender were used. (See suggestions below.)

We acknowledge and completely agree with the reviewer. In the revised manuscript, we have added CellBender to the pipeline and have thoroughly re-run all the analyses. Please see our **Summary of major modifications** described at the beginning of this letter. Importantly, despite different technical approaches used to identify and correct ambient RNAs between SoupX and CellBender, here we have demonstrated that both correction methods yield consistent results and improve downstream analyses as described in the revised manuscript.

Please see below examples of new results obtained using CellBender in the correction pipeline.

Page 13, Line 397-408:

"Similar to PBMC-derived T cell subpopulations, we observed that biological pathways driven by Hb genes were uniquely identified before ambient mRNA correction but were absent after correction with both CellBender (Fig 4B and S8 Table) and SoupX (S22 Fig and S9 Table). These pathways include GO:0007599 (hemostasis), GO:0050817 (coagulation), GO:0042743 (hydrogen peroxide metabolic process), GO:0030168 (platelet activation), GO:0015670 (carbon dioxide transport), and GO:0070527 (platelet aggregation) (Fig 4B, S22 Fig, and S8-S9 Tables). The presence of these pathways suggests that ambient Hb gene contamination can lead to misleading enrichment of erythroid- and platelet-associated processes, which are biologically unrelated to pro-B cells. Notably, after ambient mRNA removal, these pathways were no longer detected (Fig 4B, S22 Fig, and S8-S11 Tables), supporting the effectiveness of ambient contamination correction in eliminating improper signals and refining biological pathway interpretation."

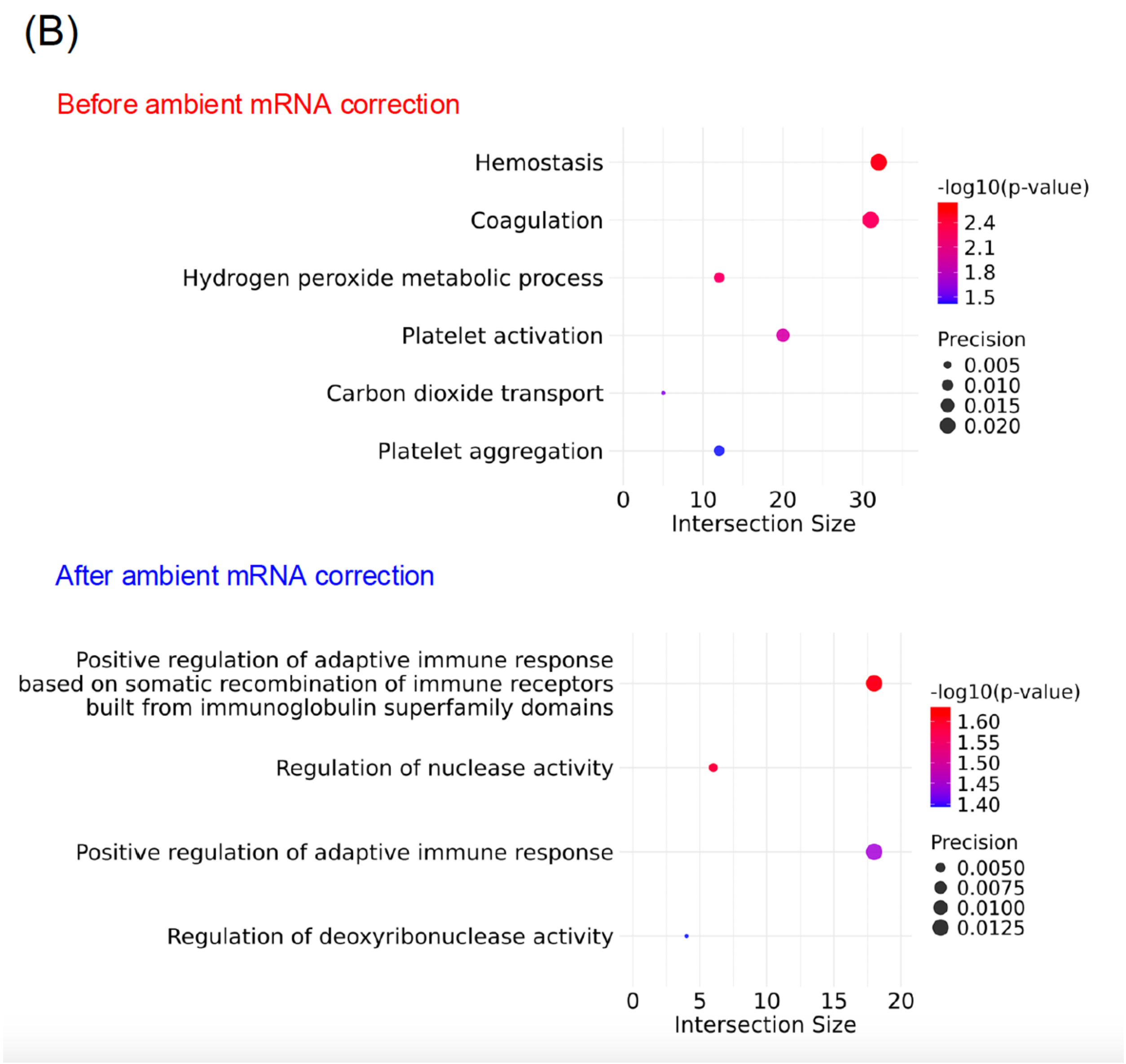

"Fig 4. Enhancement of B cell-specific DEGs and biological pathways after ambient mRNA contamination correction.

(B). The Gene Ontology Biological Processes (GO:BPs) of the DEGs from top 2000 DEGs in pro-B cells before (upper panel) and after (lower panel) ambient mRNA correction with CellBender (see S21 and S22 Figs for SoupX)."

Minor concerns:

1. The dataset integration in the UMAP in Figure S9 looks potentially problematic. Was the data integrated by the authors? How? Is that the statement in the Methods about "processed as described in the original article"? I am not sure this would give me enough information to reproduce the analysis here. What does that UMAP look like if cells are colored by "batch"?

We appreciate the reviewer's comments on reproducibility. In the revised manuscript, we have applied the same processing pipeline to all datasets, which is now clearly mentioned in this article in the "Materials and methods" sections. Please see below examples of updated "Materials and methods". Additionally, we have included UMAP plots colored by "batches" in the Supplementary materials (S8 and S17 Figs), please see below.

Page 4-5, Line 107-121:

"All datasets used in this study were processed using the same consistent pipeline described as follows. Raw FASTQ files were aligned and quantified using the CellRanger Single-Cell Software Suite (version 8.0.1) and the reference human genome GRCh38-2024-A (10x Genomics, USA). Standard preprocessing steps, including normalization of gene expression levels, scaling, clustering, and dimensionality reduction, of individual single-cell data was carried out using Seurat V.5.2.1 [22]. Expression levels of genes in each cell were normalized using the LogNormalize approach available from the *NormalizaData* function, where the unique molecular identifier (UMI) counts of each gene were divided by the total number of UMIs per cell, then multiplied by a scale factor of 10^4, and subsequently log-transformed. Cell clusters were identified using the *FindClusters* function from the Seurat package (V.5.2.1) [22] with the default settings. Dimensionality reduction was performed using the Uniform Manifold Approximation and Projection (UMAP) method via the *RunUMAP* function, with the first 10 principal components (PCs). Cells with the total mitochondrial gene expression exceeding 10% were excluded. Doublets were detected and discarded using DoubletFinder [23] with the default settings."

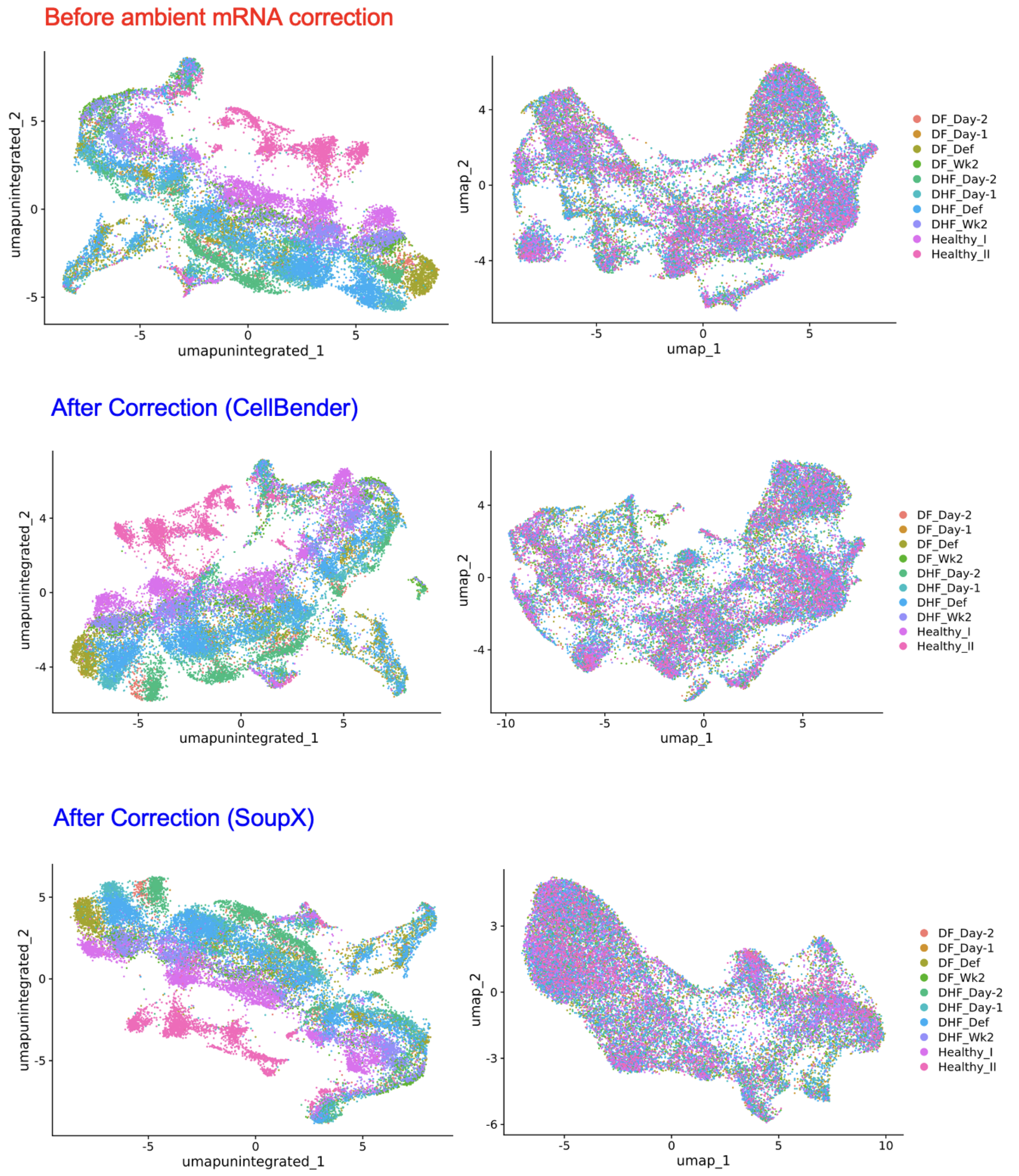

"**S8 Fig.** UMAP plots showing T cell subsets before (left panel) and after (right panel) batch effect correction using *SCTransform v2* from Seurat V.5.2.1. Colors represent individual single-cell RNA-seq libraries. Related to Fig. 3."

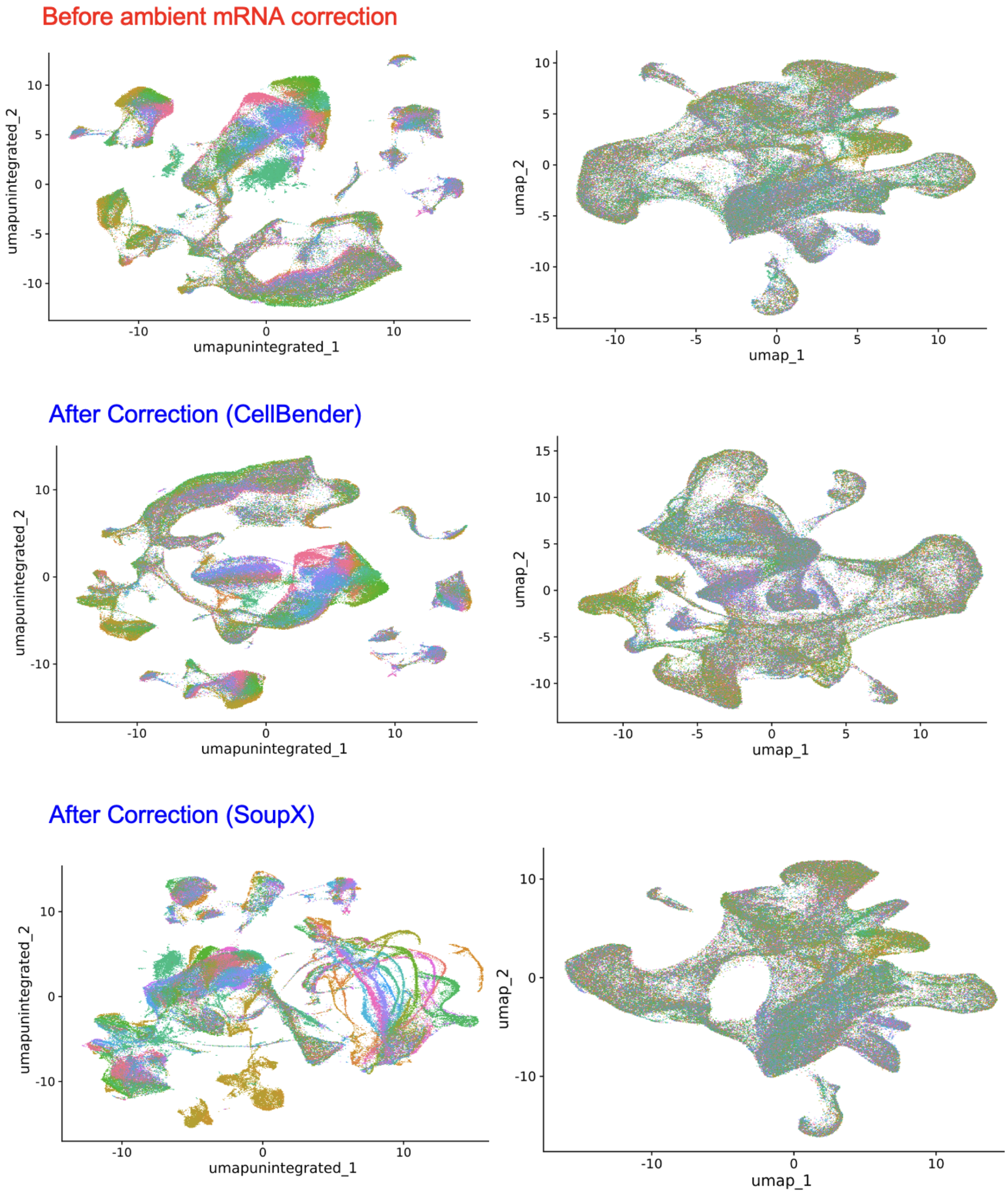

“**S17 Fig.** UMAP plots of forty-two integrated samples before (left panel) and after (right panel) batch effect correction using *SCTransform v2* from Seurat V.5.2.1. Colors represent individual single-cell RNA-seq libraries. Related to Fig. 4.”

2. CD79A is listed in the text as a B cell marker, but shows up in Table S1 as a plasma cell marker.

We thank the reviewer for pointing this out. *CD79A* is typically associated with B cells; however, its expression also persists in circulating plasma cells. We have mentioned this in the original manuscript and have now clarified it in the revised manuscript, both in the figure legend and in **Figures 1B and 2A.**

Page 7, Line 182-185:

"Similarly, B cell lineage genes *MS4A1* (B-lymphocyte-specific membrane protein) and *CD79A* (B-cell antigen receptor complex-associated protein) exhibited higher expression levels in the cells corresponding to B cells and PCs, respectively, compared to other cell types (Fig 1B and S2 Fig)."

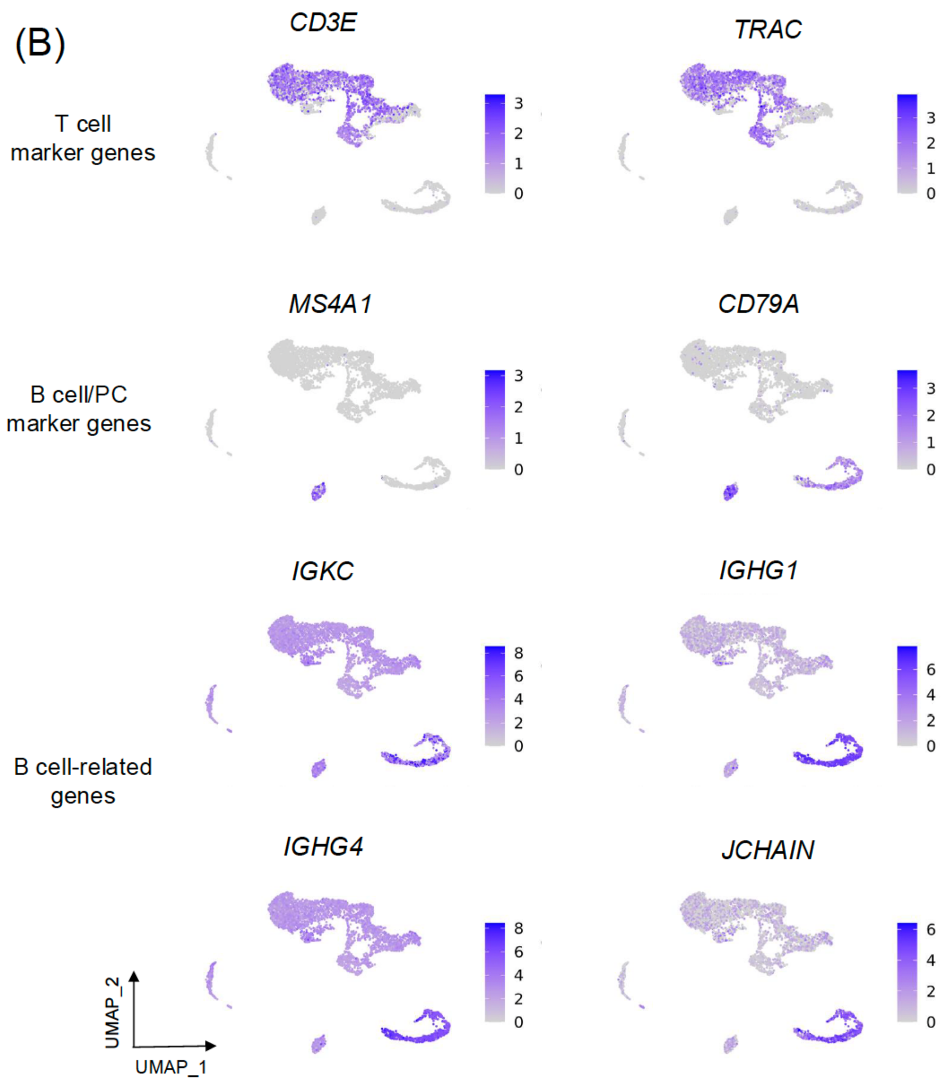

"Fig 1. Expression of B cell-related genes in non-B cell populations.

(B). UMAP feature plots demonstrating the relative transcription levels of T cell marker genes (first panel), B cell and plasma cell (PC) marker genes (second panel), and B cell-related genes (third and fourth panels). Cell types are annotated using Azimuth, supplemented by canonical cell-type marker genes (S1 Table) where relevant. pDCs = plasmacytoid dendritic cells."

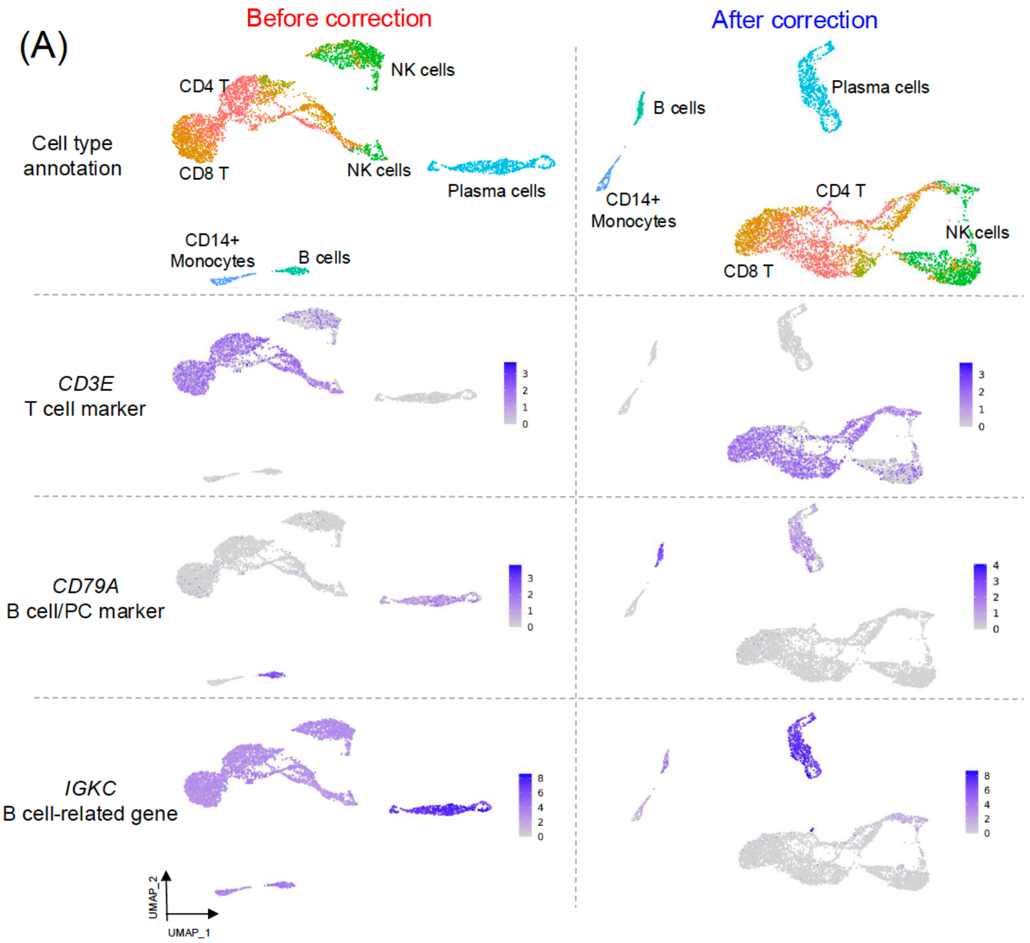

"Fig 2. Expression profiles of ambient mRNAs before and after correction.

(A). UMAP plot representing the single-cell transcriptome profiles of PBMCs before (left panel) and after (right panel) ambient mRNA correction using CellBender [17] (refer to S3 Fig for results with SoupX[20]). Cell types are annotated using Azimuth [25] and supplemented with established canonical marker genes where relevant (S1 Table and S2 Fig)."

3. It is unclear to me whether it is sound to run SoupX with a manually-provided set of hemoglobin genes, and then look at the output performance on hemoglobin genes to show that SoupX worked well. Namely "virtually no Hb genes were detected after the ambient mRNA correction." Is this a good thing? How do we know that SoupX is not overcorrecting? How do we know if SoupX worked for other genes which were not provided on the input "potential contamination gene list"?

We appreciate the reviewer's insightful question. We observed hemoglobin (Hb) gene expression across various cell types, including B cell subsets, which is biologically unexpected due to Hb genes are known markers of the red blood cell lineage (S18 Fig). However, in this revision with updated pipelines, we observed lower numbers of Hb genes and reduced expression levels after ambient mRNA correction in both CellBender and SoupX (Fig 4A and S20 Fig). To address concerns about potential overcorrection, we assessed the expression levels of other known marker genes after correction (S4 and S5 Figs). Additionally, we included an independent ambient mRNA removal tool, CellBender, which does not rely on predefined contamination genes, to cross-validate the impact of ambient mRNA removal. Our results showed consistency across both methods, suggesting that SoupX is unlikely to be overcorrecting.

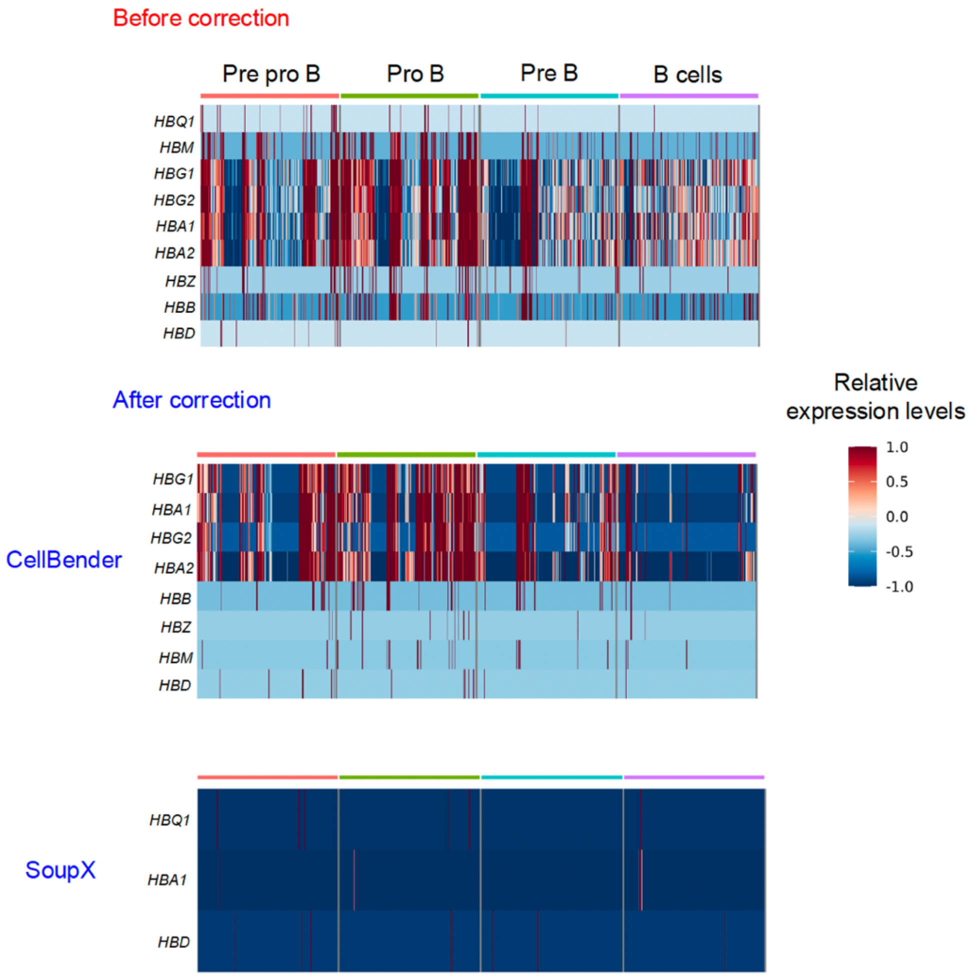

"**S20 Fig.** Heatmap demonstrating the relative expression levels of hemoglobin (Hb) genes observed in the differentially expressed gene (DEGs) between each B cell subsets, compared to the rest. DEGs were identified using the Wilcoxon rank sum test through the *FindAllMarker* function in Seurat V.5.2.1. Related to Fig 4."

4. TUBB and TUBA1B are referred to as "adhesion molecules", which I am not sure is accurate.

We thank the reviewer for pointing this out. We have replaced Fig 3B with pathway analysis instead of only focusing on the average log2 fold change of individual or known marker genes as described in the **Summary of major modifications**. Therefore, this section has been excluded in the revised manuscript.

5. Section 3.4 begins "We then asked if the improvement on cell type identification and DEG analyses as a result of ambient mRNA correction can be seen in other independent dataset…" Did the authors demonstrate improvement in cell type identification? I would be very interested to see this, but I am not sure this was clearly demonstrated here. Something like scPred (PMID: 31829268) or another automatic-cell-type-annotation tool could potentially be used to annotate the datasets before and after ambient RNA removal. Then the performance enhancement could be quantified.

We thank the reviewer for this insightful comment. In the revised manuscript, we annotated cell types using Azimuth - a reference-based tool. We did not observe any differences in annotated cell types before and after ambient mRNA correction (S9 and S19 Figs). This may be due to the robustness of the Azimuth annotation method, which relies on a well-curated reference and is less sensitive to low-levels ambient mRNA contamination. Additionally, our revised analysis focuses on the improvement of differentially expressed gene (DEG) identification and pathway analysis. These improvements were consistently observed across both independent datasets (PBMCs and human fetal liver tissues) and ambient removal tools (CellBender and SoupX).

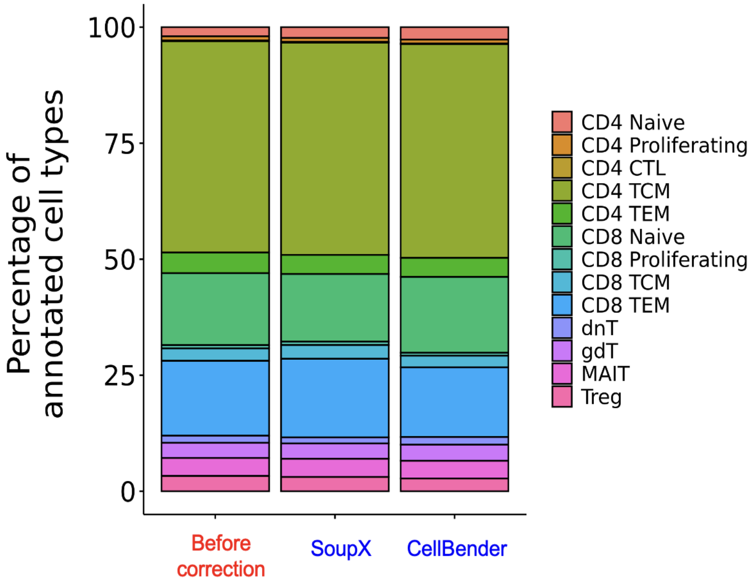

"S9 Fig. Bar plot showing the percentages of annotated T cell subpopulations using Azimuth, supplemented by known canonical marker genes before and after correction. Colors represent different cell types. Related to Fig 3."

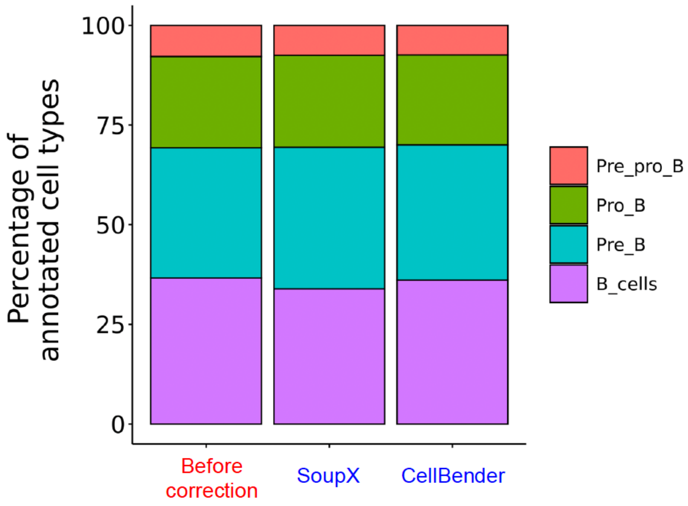

"S19 Fig. Bar plot showing the percentages of annotated B cell subpopulations using Azimuth, and the original article's annotation [21]. Colors represent different cell types. Related to Fig 4."

6. How are there so many B cell populations in Fig. 4 and Fig. S11? There appear to be 12 B cell clusters after correction. This seems like a large number of clusters of B cells. Are these biological meaningful clusters, and how can a reader be convinced of that? Or are we over-clustering here? What does this UMAP look like if cells are colored by “batch”?

Thank you for your thoughtful question. Initially, our focus was on comparing before and after ambient mRNA correction using the same clustering resolution, without specifically adjusting for cell type or stage annotations. Indeed, the observed B cell clusters could be the same subpopulation distinguished by differences in immunoglobulin light/heavy subfamily genes or they could result from over-clustering, as you mentioned. Determining whether these clusters are biologically meaningful would be interesting and could be assessed by looking at the marker genes in each cluster. However, in this revision, we have excluded this clustering analysis and focused on identifying differentially expressed genes (DEGs) and performing pathway analyses before and after ambient mRNA correction.

Regarding batch effects, we could confirm that no batch effects were observed, and we have included UMAP plots showing the data before and after batch correction in this revised manuscript (S8 and S17 Figs), ss shown above.

7. I was confused about the point being made in Fig. 4. What is the relationship between the cells in “before correction” cluster 13 and “after correction” clusters 10 and 11? Are they the same cells? In general, how do the authors identify which “before correction” cluster corresponds to which “after correction” cluster (especially as there are different numbers of clusters)?

We appreciate the reviewer’s question. The cells in cluster 13 before correction share the same cell IDs as those in clusters 10 and 11 after correction. However, in the revised manuscript, we have excluded this specific separation of clusters per the reviewer’s suggestions, several factors influence clustering, making it challenging to systematically evaluate cluster quality. Instead, we have expanded our study incorporating pathway analyses beyond differentially expressed genes (DEGs) to provide more biologically meaningful insights (as described in **Summary of major modifications**).

8. What does Fig. 4C look like “before correction”, if you use the same “after correction” cluster labels for cluster 10 and 11? Are the histone-related gene markers something that is only seen after ambient RNA correction?

We appreciate the reviewer’s question. Since we have excluded the cluster separation in the revised manuscript, we no longer compare before and after correction clusters in this manner. Additionally, due to updates in the analysis pipelines and package versions, we no longer retain

the original datasets used from the initial submission. As a result, we are unable to determine whether histone-related gene markers were exclusively observed after ambient mRNA correction.

Suggestions:

This study would be much more impactful if it either (1) went further with analyses of downstream effects, and/or (2) compared multiple methods of ambient RNA removal, rather than SoupX alone.

Thank you very much for your valuable feedback. We have added new aspect of downstream analysis, pathway enrichment analysis, which is one of the common goals of single-cell RNA-seq studies, to provide deeper insight into the effect of ambient mRNA contamination. Additionally, we have expanded our analysis by applying CellBender alongside SoupX in the revised manuscript.

Recommendations:

I would really like to see a study like this published, and I think the authors have followed an interesting line of work. However, I have some concerns about whether the current analyses as presented go farther than what is shown in the literature. In particular, there is work that seems comparable in Figures 3 and 4 of [15], or Figure 2 and 3 of [14], or Figure 4 and 5 of [16]. I recommend this article should be published with some subset of the above-suggested revisions.

Thank you for your insightful comments and the opportunity to improve our study. We acknowledge and agree with all your suggestions and have done all best to incorporate the suggestions into the revised manuscript when possible. As mentioned in detail, in this revision we have expanded our analyses beyond differential gene expression by implementing pathway enrichment analysis, which provides a broader functional perspective on the effects of ambient mRNA contamination. Additionally, we have incorporated CellBender alongside SoupX to comprehensively evaluate the impact of contamination, independent of the ambient RNA removal tool used.

Reviewer #2

I appreciate the opportunity to review this manuscript titled ' Understanding and mitigating the impact of ambient mRNA contamination in single-cell RNA-sequencing analysis'. The study addresses an important topic in single cell RNA-sequencing, which is relevant to advancing our understanding of how ambient noise interferes with downstream analyses. The authors have made commendable efforts in investigating the impacts of ambient mRNA noise in scRNA-seq analysis. Below, I provide my detailed comments and suggestions to help enhance the clarity, rigor, and overall quality of the manuscript.

We thank the reviewer's thoughtful comments and valuable suggestions. Most of all, we appreciate that the reviewer kindly recognised the importance of the interferences from ambient RNA contamination, as this can be frequently overlooked despite its being vital to a number of downstream bioinformatic analyses and biological interpretation.

In response to the valuable comments from both reviewers and editor, we have added a number of new analyses and modifications to the revised manuscript. Please see also Summary of major modifications at the beginning of this letter. We hope that we have addressed the majority of your key suggestions and have done all best to incorporate the suggestions into the revised manuscript when possible.

Major comments:

1. The authors claim that they "investigate the impact of ambient mRNA contamination on downstream bioinformatic analyses" using two datasets. For this, they should provide a comprehensive description of noise level removed by SoupX in the datasets analyzed. No objective criteria were introduced to measure the level of noise in the datasets. Instead, the authors claimed that the two dataset they have studied had "high" level of noise.

We thank the reviewer for their insightful comments. We agree that it is important to add more quantitative evaluation of the impact of ambient mRNA contamination. To this end, we have performed additional analyses as described in **Summary of major modifications** at the beginning of this letter to improve this aspect. More specifically on the level of contamination (presumably that is what the reviewer referred to as noise, if we are not mistaken), as shown on Page 6, line 165-167 and in new S1 Fig, we now show the relative expression levels of Ig genes (known B cells and Plasma cell marker genes) across annotated cell types using Azimuth, which demonstrated the presence of background noise in single-cell datasets [17, 19, 20].

Page 6, Line 165-167:

"The dataset exhibited contamination from ambient mRNA s or background noise, as evidenced by the presence of nonzero counts of known marker genes in unexpected cell types [17, 19, 20] (S1 Fig)."

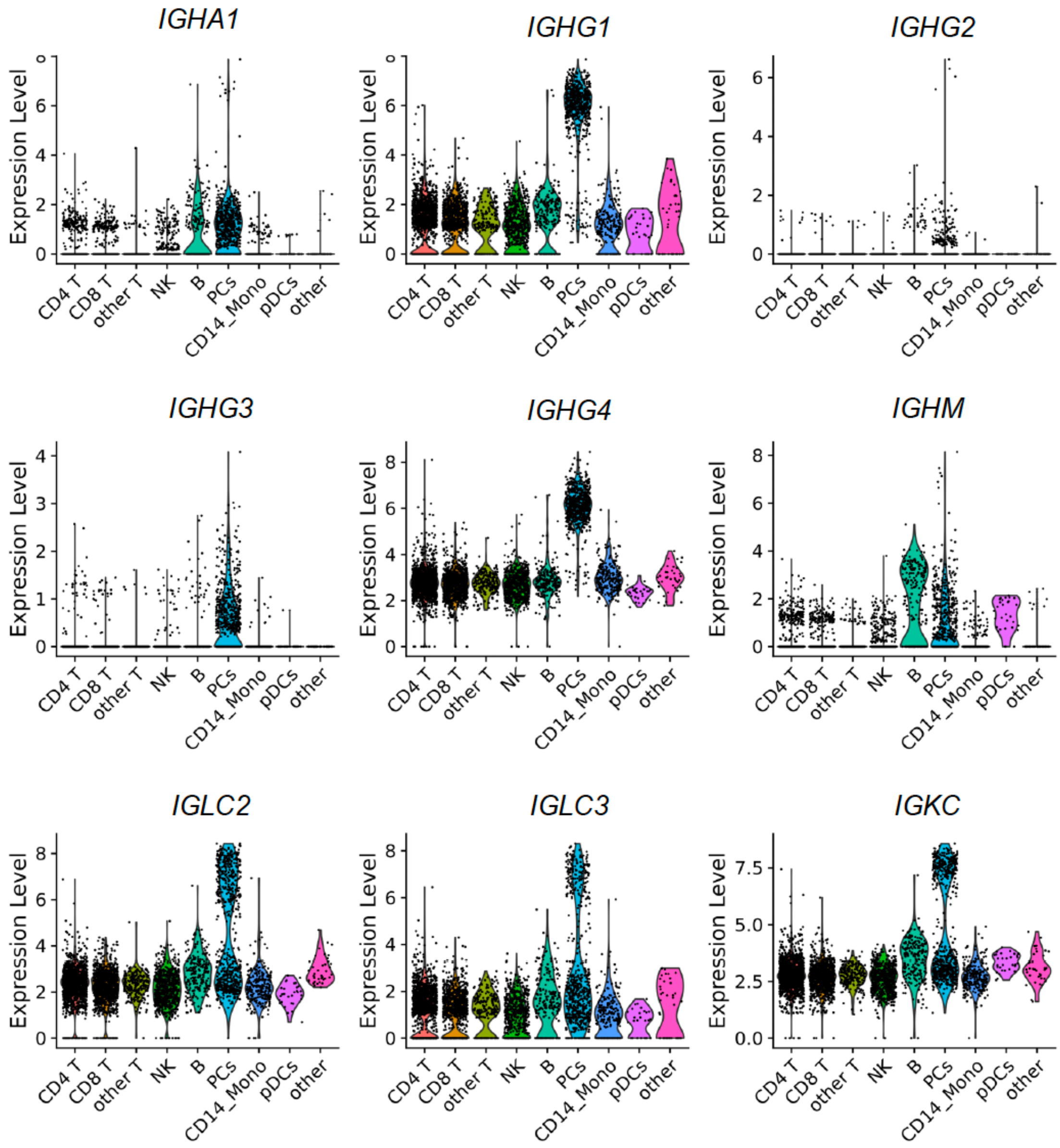

"**S1 Fig.** Violin plots demonstrating the normalised expression levels of immunoglobulin (Ig) genes, canonical marker genes of B and plasma cells, across cell types annotated using Azimuth and known canonical marker genes (S1 Table) where relevant. Related to Fig 1."

Addition, we have applied the automated approach provided by SoupX to estimate a set of ambient mRNA contamination genes in an unbiased manner for both datasets in our study. Please see below.

Page 8, Line 216-218:

"Evidently, Ig genes were indeed identified as potential ambient mRNA contamination among the top 10 genes predicted by SoupX (S2 Table)."

Page 12, Line 362-364:

"After data integration with no batch effects (S17 Fig), hemoglobin (Hb) genes were identified as potential ambient mRNA contaminants, ranking among the top five genes predicted by SoupX (S7 Table)."

2. I suggest that the authors simulate different unbiased levels of ambient noise and add these noise levels to a cleaned dataset and then compare how different levels of noise affects the downstream analyses.

We appreciate the reviewer's thoughtful suggestion. We acknowledge the importance of simulating different unbiased levels of ambient mRNAs and assessing their impact on downstream analyses, which would be an interesting direction for future research. However, we believe the main objective of the work, which is on the presence of ambient RNA contamination and its subsequent impact on the downstream analyses, could mostly be covered using "real-world" datasets. Importantly, we observed consistent results across two independent datasets and demonstrated that our findings remain valid when using two different ambient mRNA removal tools, CellBender and SoupX. Saying that, we acknowledge that the simulated dataset will be extremely useful for in-depth analyses of different levels of contamination on downstream analyses, and hence we have now mentioned this in the discussion.

Page 14, Line 418-423:

"Taken together, our study highlights the practical applications of mitigating ambient mRNA contamination, including improved DEG identification and enhanced specificity of cell type-specific gene expression profiles and biological pathways in actual biological scRNA-seq datasets. We note that, systematically simulating varying unbiased levels of ambient mRNA contamination and evaluating their impact on downstream analyses represents a promising direction for further research."

Section 3.1:

3. The authors claim that the dataset used for this section exhibits high levels of mRNA contamination. This is very subjective. If there is a measure of contamination which they have used to screen datasets for the high level of noise, it should be mentioned.

We appreciate the reviewer's thoughtful comment. We acknowledged that descripting the dataset as having "high levels of mRNA contamination" can be subjective. To clarify this point, we have revised the manuscript (please see below) and added new S1 Fig and S2 and S7 tables as follows:

Additionally, we have removed the previous manuscript.

", which exhibited high levels of ambient mRNA contaminations across a wide range of well-characterized cell types [15, 18]."

Page 6, Line 165-167:

"The dataset exhibited contamination from ambient mRNA s or background noise, as evidenced by the presence of nonzero counts of known marker genes in unexpected cell types [17, 19, 20] (S1 Fig)."

Page 8, Line 216-218:

"Evidently, Ig genes were indeed identified as potential ambient mRNA contamination among the top 10 genes predicted by SoupX (S2 Table)."

Page 12, Line 362-364:

"After data integration with no batch effects (S17 Fig), hemoglobin (Hb) genes were identified as potential ambient mRNA contaminants, ranking among the top five genes predicted by SoupX (S7 Table)."

S2 Table. Top 50 potential ambient mRNA genes predicted by SoupX (dengue infection-dataset)

	est	counts
IGKC	0.0895851981	258621
IGHG4	0.02770022363	79967
MALAT1	0.01807042363	52167
B2M	0.01639005817	47316
IGHA1	0.01298983814	37500
IGLC2	0.01174766321	33914
IGHG1	0.01143348233	33007
TMSB4X	0.0106894244	30859
JCHAIN	0.007366450608	21266
RPL10	0.006515010018	18808

S7 Table. Top 50 potential ambient mRNA genes predicted by SoupX (fetal liver tissue-dataset)

	est	counts
HBG2	0.08837919456	130818
HBG1	0.03060823273	45306
HBA2	0.02070207203	30643
HBA1	0.01734574615	25675
MALAT1	0.01140191462	16877

Section 3.2:

4. The use of SoupX because of its convenience is not relevant. Using SoupX is highly subjective and depends on domain knowledge and user input. The authors could use CellBender to do ambient noise removal for it provides an unbiased processing.

Thank you for your valuable comment. In this revision, we have implemented CellBender alongside SoupX to comprehensively investigate the impact of ambient mRNA contamination, regarding the ambient removal tool used.

5. Instead of exploring pre-selected set of genes like Ig or Hb genes, the authors should provide the top genes with high contamination fraction and select form those.

We thank the reviewer for the valuable comment. The pre-selected genes we applied were indeed predicted by SoupX. We agree that identifying high-contamination genes in an unbiased manner is important to mention. We have included this information in the revised manuscript. Please see below.

Page 8, Line 216-218:

"Evidently, Ig genes were indeed identified as potential ambient mRNA contamination among the top 10 genes predicted by SoupX (S2 Table)."

Page 12, Line 362-364:

"After data integration with no batch effects (S17 Fig), hemoglobin (Hb) genes were identified as potential ambient mRNA contaminants, ranking among the top five genes predicted by SoupX (S7 Table)."

S2 Table. Top 50 potential ambient mRNA genes predicted by SoupX (dengue infection-dataset)

	est	counts
IGKC	0.0895851981	258621
IGHG4	0.02770022363	79967
MALAT1	0.01807042363	52167
B2M	0.01639005817	47316
IGHA1	0.01298983814	37500
IGLC2	0.01174766321	33914
IGHG1	0.01143348233	33007
TMSB4X	0.0106894244	30859
JCHAIN	0.007366450608	21266
RPL10	0.006515010018	18808

S7 Table. Top 50 potential ambient mRNA genes predicted by SoupX (fetal liver tissue-dataset)

	est	counts
HBG2	0.08837919456	130818
HBG1	0.03060823273	45306
HBA2	0.02070207203	30643
HBA1	0.01734574615	25675
MALAT1	0.01140191462	16877

Technical comments:

1. The overall bioinformatic analyses performed for this manuscript are weak.

We appreciate the reviewer's feedback. In this revision, we have implemented carefully designed computational approaches, including pathway enrichment analysis, and two independent ambient mRNA removal tools with different approaches – CellBender, which perform automated correction, and SoupX, which corrects ambient mRNA expression levels using a potentially predefined ambient gene set – to comprehensively reveal the impact ambient mRNA contamination in single-cell datasets. Additionally, we have carefully addressed potential confounders such as batch effects and double identification.

2. There are better doublet identification methods like scDblFinder.

Thank you for your suggestion. We acknowledge that scDblFinder may be a better option for doublet identification. Regarding whether doublet detection should be performed before or after ambient mRNA removal, we selected DoubletFinder because it is designed to run on clean data, meaning that ambient mRNA decontamination would be performed before doublet doublet identification. Additionally, as mentioned in the scDblFinder (section "What about ambient RNA decontamination" https://plger.github.io/scDblFinder/articles/scDblFinder.html#frequently-asked-questions), a contaminated cell could look like a doublet and vice versa, meaning that decontamination could potentially interfere with the doublet identification approach, making this choice important to consider.

3. No batch information was provided for any of the datasets. The UMAPs with batch information before and after batch effect correction could be supplemented.

We appreciate the reviewer's comment regarding batch effect information. We have included UMAP plots showing the before and after batch effect correction of all datasets presented in this study.

Please refer to Page 9, Line 257-258:

"Following the integration of ten scRNA-seq profiles from the same representative study [13], we observed no apparent batch effects in T cells (S8 Fig)."

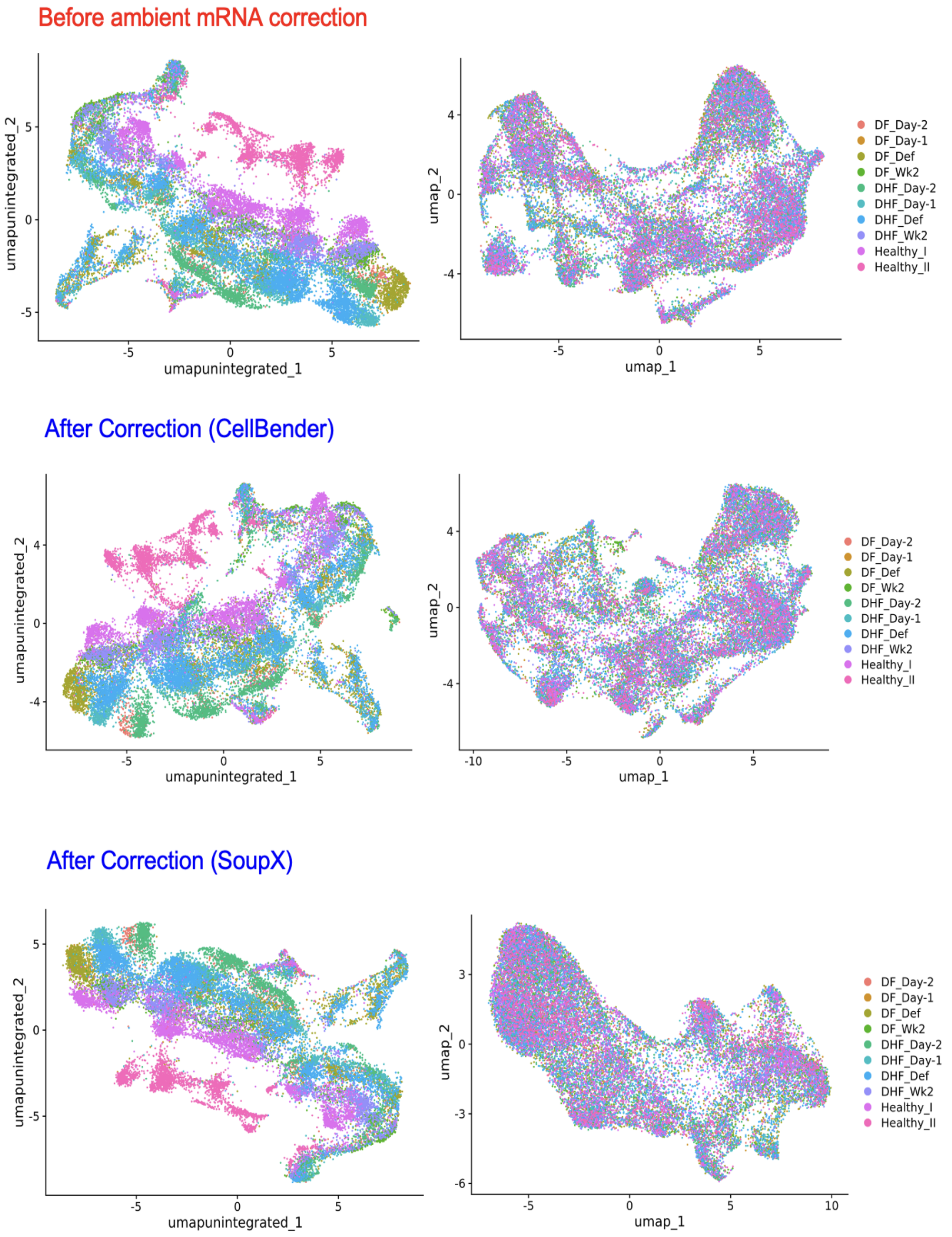

"**S8 Fig.** UMAP plots showing T cell subsets before (left panel) and after (right panel) batch effect correction using *SCTransform v2* from Seurat V.5.2.1. Colors represent individual single-cell RNA-seq libraries. Related to Fig. 3."

Please refer to Page 12, Line 362-364:

"After data integration with no batch effects (S17 Fig), hemoglobin (Hb) genes were identified as potential ambient mRNA contaminants, ranking among the top five genes predicted by SoupX (S7 Table)."

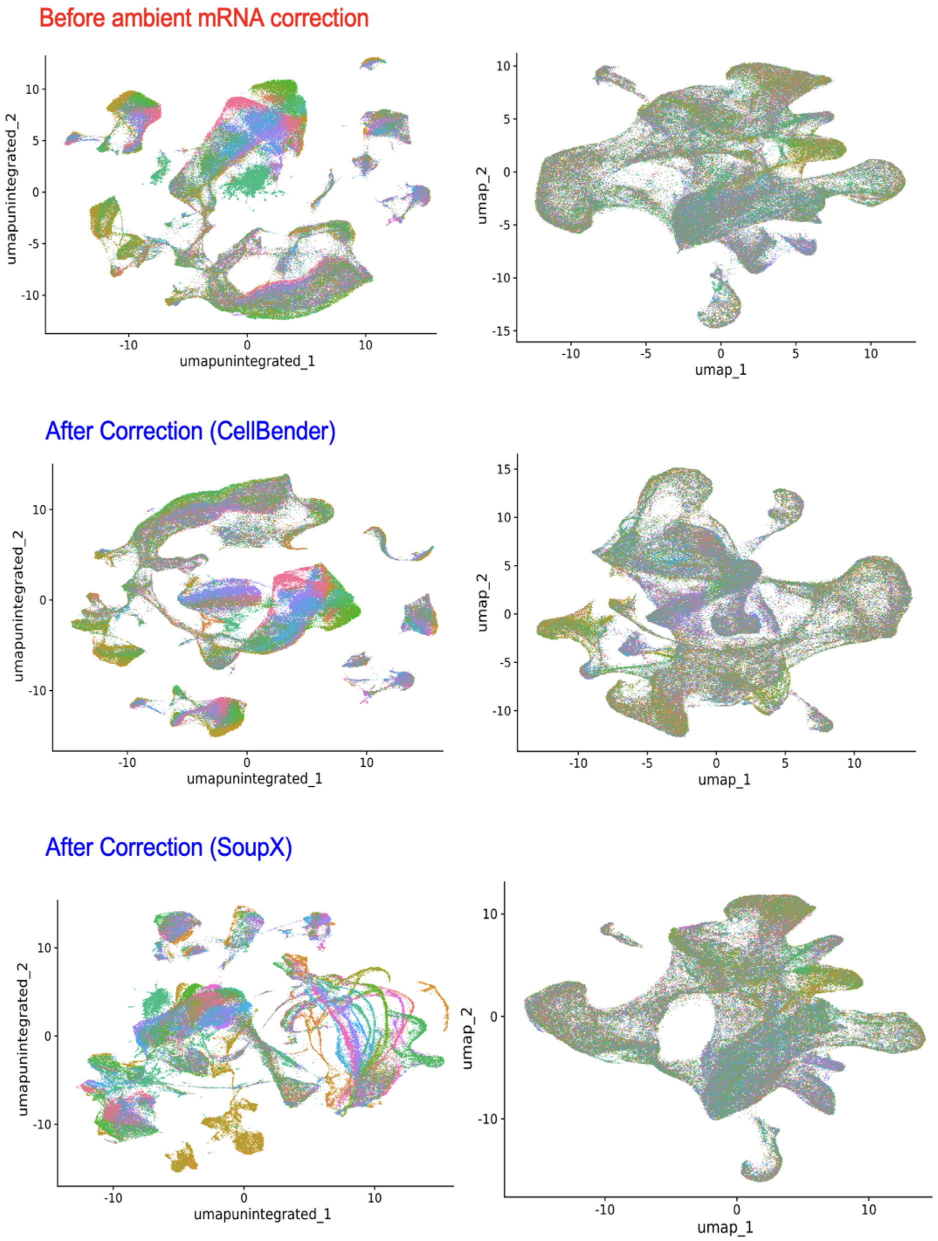

"**S17 Fig.** UMAP plots of forty-two integrated samples before (left panel) and after (right panel) batch effect correction using *SCTransform v2* from Seurat V.5.2.1. Colors represent individual single-cell RNA-seq libraries. Related to Fig. 4."

4. The choice of Wilcox rank sum test for DE is not appropriate. This test is associated with high rate of false positive results. Use of pseudobulk is recommended. Refer to PMID: 34584091.

We thank the reviewer for raising this important point. We have performed differential gene expression (DEG) analysis using both the Poisson generalized linear model and the Wilcoxon rank-sum test. Our results suggest that while differences exist between statistical approaches, there is a high degree of overlap in DEGs and pathway enrichment analyses in both datasets, particularly in the first dataset - T cell subpopulations from dengue infection patients. Given the agreement observed between the Poisson generalized linear model and the Wilcoxon rank-sum test in our study, we believe our results remain valid and biologically meaningful.

Importantly, we acknowledged that the optimal DEG approach may depend on dataset characteristics, as variation between datasets can influence statistical outcomes.

Please find the details below:

T cell subpopulations (Fig 3)
Our results indicate that the majority of DEGs identified using these two statistical methods overlapped significantly (> 90%) in all three conditions (before correction, after CellBender, and after SoupX) (Fig R1), leading to comparable pathway enrichment results.

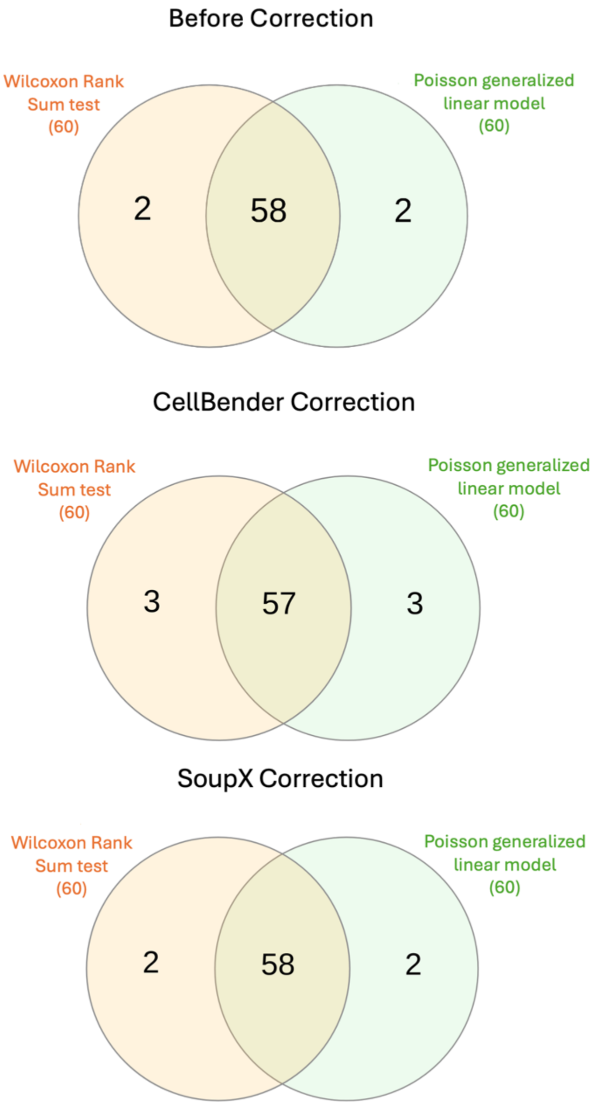

Fig R1. Venn diagram showing the overlap of the top 20 DEGs in each biological condition (acute, convalescent, or healthy control) compared to the other two, across T cell subpopulations. DEGs were identified using the Wilcoxon Rank Sum Test (left) and the Poisson generalized linear model (right).

Pro-B cells (Fig 4)

For the second dataset, we observed a higher number of overlapping top 2000 DEGs between the two statistical methods after ambient mRNA correction, compared to before correction (Fig R2). Specifically,

- Before correction: 691 overlapping DEGs, with 1309 unique to each statistical method.
- After CellBender: 1872 overlapping DEGs, with 128 unique to statistical method.
- After SoupX: 1869 overlapping DEGs, with 104 unique to statistical method.

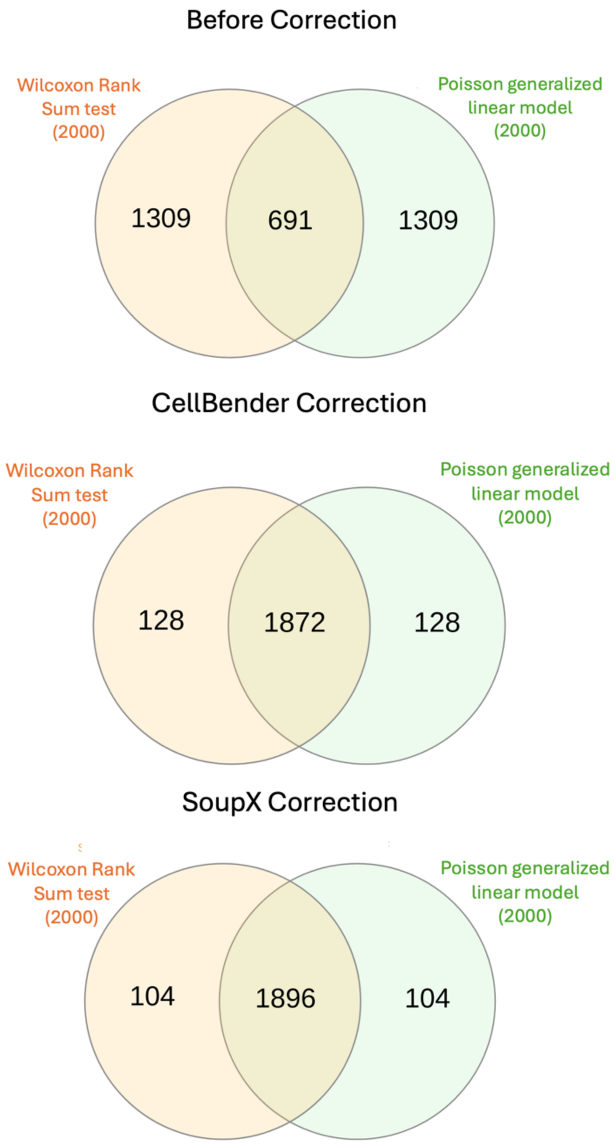

Fig R2. Venn diagram showing the overlap of the top 2000 DEGs in Pro-B cells, compared to the rest. DEGs were identified using the Wilcoxon Rank Sum Test (left) and the Poisson generalized linear model (right).

Furthermore, pathway enrichment analysis (Fig R3) revealed that

- The GO:BPs identified from the top 2000 DEGs in Pro-B cells using both statistical methods largely overlapped in all three conditions (before correction, after CellBender, and after SoupX correction).
- Before correction (Fig R3; top panel): Hemoglobin gene-related pathways were identified in both statistical methods. However, one GO:BP was uniquely found in the Wilcoxon rank-sum test (Fig R3; top left panel).
- After CellBender (Fig R3; bottom left panel): Two Pro-B cell gene-related pathways were observed in both statistical methods and two GO:BPs uniquely identified in the Wilcoxon rank-sum test.
- After SoupX (Fig R3; bottom right panel): All Pro-B cell gene-related pathways were consistently observed in both statistical methods.

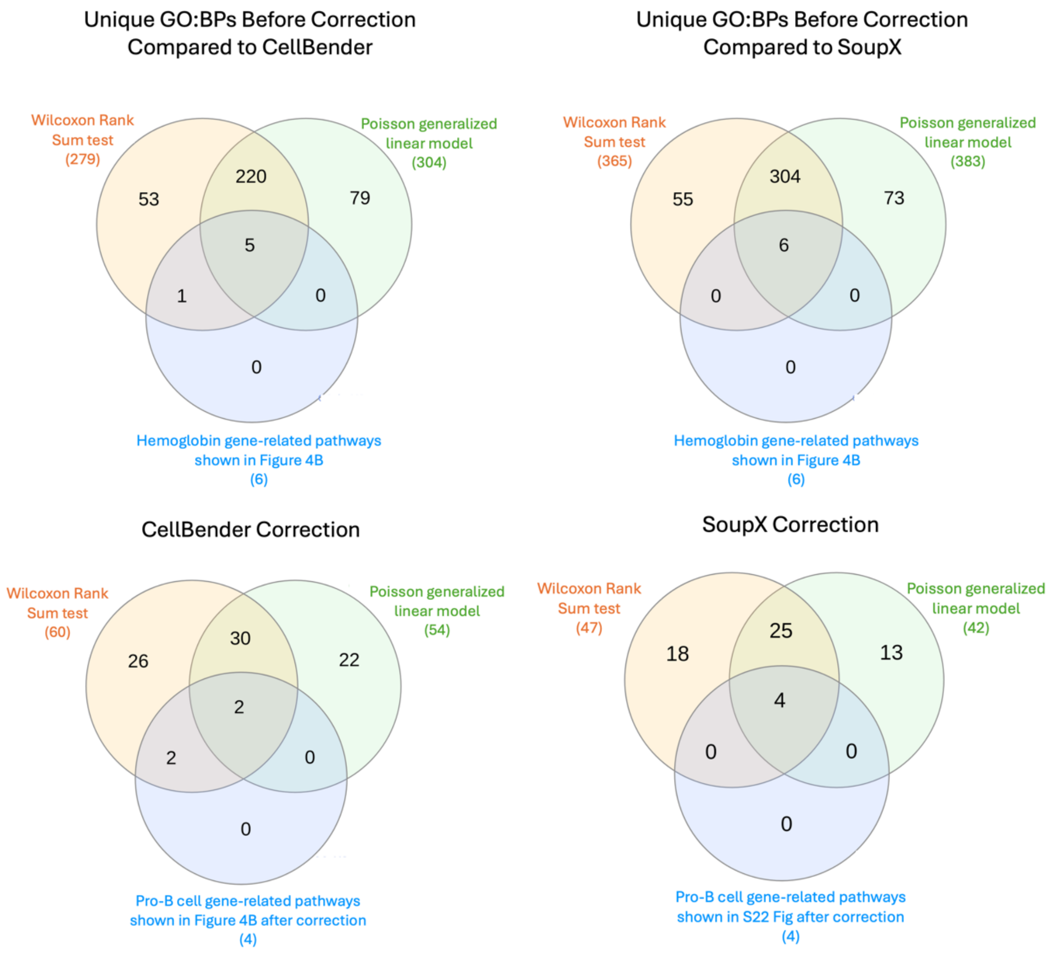

Fig R3. Venn diagram showing the overlap of GO: BPs identified from the top 2000 DEGs in Pro-B cells, compared to the rest. The top panel represents results before correction, while the bottom shows results after ambient mRNA contamination using CellBender (left) and SoupX (right).

5. No objective criteria were used to assess and compare clustering quality before and after noise removal. Silhouette score or Davies-Bouldin Index could be used.

We thank the reviewers for this suggestion. In this revision, we have excluded the clustering analysis, as several factors influence clustering, making it challenging to objectively evaluate cluster quality. We have instead demonstrated the impact of ambient mRNA contamination on biological pathway enrichment, which is a common goal of many single-cell studies. Please see also **Summary of major modifications** at the beginning of this letter.

Minor comments:

Section 3.1:

1. the authors have called cell types based on the module scores of some canonical markers for each cell types and cells with scores > 0 were called. The authors should demonstrate that how robust is this cell typing to the presence of the ambient mRNA.

We appreciate the reviewer's valuable comment. In this revision, we have applied Azimuth, a reference-based cell type annotation tool, to enhance the robustness of cell type identification and minimise the influence of ambient mRNA contamination. The numbers of annotated cell types remain relatively comparable before and after correction, suggesting that the annotation is not impacted by contamination. Additionally, we also excluded non-T cells using well-characterised marker genes to ensure precise and accurate cell type identification. We have included this information in our revised manuscript.

Please refer to Page 9, Line 263-264:

"The number of annotated T cells remained relatively comparable before and after ambient mRNA correction (S9 Fig), suggesting that contamination did not impact the annotation."

2. Line 200. Presence of CD3 and TRAC among markers makes the topic for figure 1 inappropriate.

We thank the reviewer for this comment. *CD3E* (a core component of T cell receptor) and *TRAC* (a part of the T cell receptor alpha chain) are well-characterised T cell markers. We included these two markers to confirm the identity of T cells (non-B cells) in the context of observing B cell-related genes in non-B cells.

3. Lines 205 to 211 are not adding any information to what was mentioned in lines 60-65 in introduction.

We appreciate the reviewer for pointing this out. We have excluded this redundant information from the introduction (previously in Lines 60-65), please refer to Page 3, Line 60-64.

Page 3, Line 60:64:

"Despite the several advantages of droplet-based single-cell technologies, one of the important challenges is the contamination of cell-free mRNAs, frequently referred to as "ambient mRNA", in scRNA-seq datasets. This ambient mRNA contamination can significantly confound biological interpretation of scRNA-seq data, as demonstrated in previous studies [15-20]."

Section 3.2:

4. In Figure 2, the authors must use the same UMAP coordinates for cells before and after correction so that it would help reader to compare the expression levels before and after correction in feature plots.

We appreciate the reviewer's valuable feedback. Using the same UMAP coordinates before and after ambient mRNA corrections could help with comparison, as suggested. However, this approach may not be appropriate because the two datasets differ in cell numbers and overall gene expression profiles, which significantly influence the different UMAP structure and may introduce biases in interpretation, potentially misrepresenting the true data structure. Moreover, maintaining identical UMAP coordinates may cause confusion, as it might incorrectly suggest that the datasets remain unchanged after correction, whereas in reality, differences are expected.

5. Lines 238-246: the authors should discuss the remaining 2 modal expression of IGKC and IGHG4 which is still present after correction in B cells. The authors should provide the total number of cells and number of cells expressing these two markers before and after correction in both non-B and B cells with the violin plots.

We appreciate this thoughtful comment. The remaining of *IGKC* and *IGHG4* expression after correction was consistently observed in both CellBender (Fig 2B) and SoupX (S3 Fig), suggesting that complete removal of ambient mRNA contamination is challenging. As suggested, we have now provided the total number of cells and the number of cells expressing these markers before and after correction (S3 Table).

Please refer to Page 9, Line 239-240:

"Additionally, we observed a reduction in the percentage of cells expressing these genes after correction (S3 Table)."

S3 Table. Number and percentage of cells expressing Ig genes.

Before correction vs CellBender								
Genes	Non B cells				B cells			
	Before correction		After CellBender		Before correction		After CellBender	
	nunber_cells	percent_cell	nunber_cells	percent_cell	nunber_cells	percent_cell	nunber_cells	percent_cell
IGKC	4182	99.88	2372	56.49	886	99.89	873	98.87
IGHG1	3073	73.39	2100	50.01	846	95.38	778	88.11
IGHG4	4026	96.15	3377	80.42	880	99.21	839	95.02
JCHAIN	2999	71.63	2106	50.15	864	97.41	831	94.11
CD3E	3503	83.66	3512	83.64	475	53.55	450	50.96
CD3D	3414	81.54	3433	81.76	484	54.57	447	50.62
MS4A1	2072	49.49	2101	50.04	510	57.50	511	57.87
CD79A	2132	50.92	2132	50.77	810	91.32	814	92.19
Total_cells	4187		4199		887		883	

Before correction vs SoupX								
	Non B cells				B cells			
	Before correction		After SoupX		Before correction		After SoupX	
	nunber_cells	percent_cell	nunber_cells	percent_cell	nunber_cells	percent_cell	nunber_cells	percent_cell
IGKC	4182	99.88	2399	57.32	886	99.89	861	97.18
IGHG1	3073	73.39	2110	50.42	846	95.38	765	86.34
IGHG4	4026	96.15	2484	59.35	880	99.21	810	91.42
JCHAIN	2999	71.63	2119	50.63	864	97.41	855	96.50
CD3E	3503	83.66	3516	84.01	475	53.55	468	52.82
CD3D	3414	81.54	3426	81.86	484	54.57	476	53.72
MS4A1	2072	49.49	2114	50.51	510	57.50	493	55.64
CD79A	2132	50.92	2174	51.95	810	91.32	819	92.44
Total_cells	4187		4185		887		886	

Section 3.4:

6. In the second dataset, the cell compositions should be explained.

We appreciate the reviewer's valuable comment. In the second dataset, the overall cell compositions remain relatively comparable before and after correction, suggesting that the annotation is not impacted by contamination – similar to the T cell subsets mentioned above. We have included this information in our revised manuscript.

Please refer to Page 13, Line 370-372:

"Similar to T cell subsets, the number of annotated B cell subpopulations remained relatively comparable before and after ambient mRNA correction (S19 Fig)."

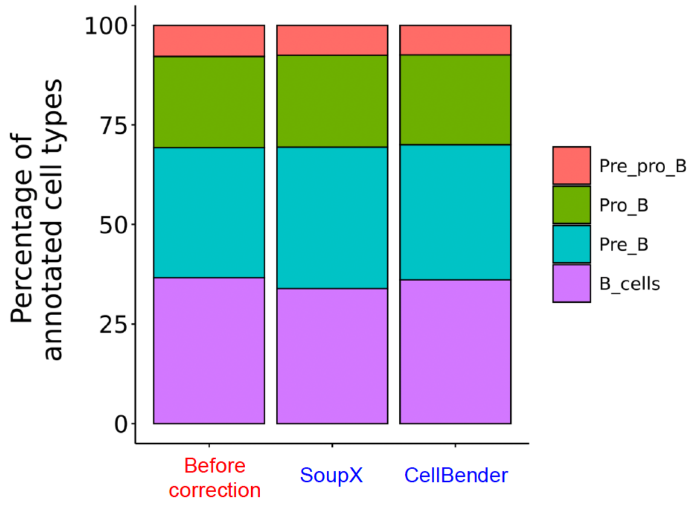

"**S19 Fig.** Bar plot showing the percentages of annotated B cell subpopulations using Azimuth, and the original article's annotation [21]. Colors represent different cell types. Related to Fig 4."
