# Supplementary material for: Understanding and mitigating the impact of ambient mRNA contamination in single-cell RNA-sequencing analysis
Source: PLoS One. 2025 Sep 24;20(9):e0332440. doi: 10.1371/journal.pone.0332440 (PMC12459771; doi:10.1371/journal.pone.0332440)
Supplement: Attachment — Submitted filename: Response_to_Reviewers_auresp_2.pdf [file pone.0332440.s024.pdf]

Dear Academic Editor and Reviewers,

Thank you for your comments and for the opportunity to revise our manuscript. We have addressed the following.

Please review your reference list to ensure that it is complete and correct. If you have cited papers that have been retracted, please include the rationale for doing so in the manuscript text, or remove these references and replace them with relevant current references. Any changes to the reference list should be mentioned in the rebuttal letter that accompanies your revised manuscript. If you need to cite a retracted article, indicate the article's retracted status in the References list and also include a citation and full reference for the retraction notice.

We have reviewed all cited references to ensure they are complete and accurate. None of the references cited in our manuscript have been retracted to our knowledge.

Reviewers' comments:

Review Comments to the Author

Reviewer #3:

1. For introduction part: regrading impact of ambient mRNA contamination in single-cell RNA-sequencing analysis on differential gene expression and biological pathway enrichment analyses, can authors explain the differences between real biological findings and artifacts induced by ambient mRNA contamination?

We appreciate the reviewer's thoughtful comment. We have incorporated an example of artifacts induced by ambient mRNA contamination into our revised manuscript.

Please refer to Page 3, Line 58-67

Despite the several advantages of droplet-based single-cell technologies, one of the important challenges is the contamination of cell-free mRNAs, frequently referred to as "ambient mRNA". This contamination can significantly confound the biological interpretation of single-cell datasets, as demonstrated in previous studies [15-20]. In brain single-nuclei RNA sequencing, for example, previously annotated neuronal cell types were separated by ambient mRNA contamination and immature oligodendrocytes were found to be contaminated with ambient mRNAs [16]. However, after computational removal of this ambient contamination, committed oligodendrocyte progenitor cells (a rare population) were detected, which had not been annotated in most previous adult human brain datasets [16]. This underscores how ambient mRNA contamination can impact cell type annotation.

2. Methods/results: authors may use simulated dataset to address impact of ambient mRNA contamination in single-cell RNA-sequencing analysis on differential gene expression and biological pathway enrichment analyses in following different simulated matrix if ambient mRNA contamination are due to following reasons:

1) Cell death or damage: background gene expression signals; Marker genes appearing unexpectedly in non-target cells (false positives); Abnormally elevated housekeeping, ribosomal, and mitochondrial genes

2) Cross-contamination in library preparation: Low-level expression of highly cell-type-specific genes in unrelated cell populations

3) Improper cell concentration: Overloaded cells result in multiple cells per droplet (doublets); Mixed expression patterns from multiple cell types (e.g., T cell and monocyte markers simultaneously detected).

We thank the reviewer for this insightful comment. We truly appreciate that simulated datasets would give valuable insights into the effect of ambient mRNA contamination on downstream analyses. Moreover, it would be interesting to investigate the downstream analyses across these three specific scenarios that can occur in a real single-cell experiment.

In this revision, we have tried our best given the timeframe of revision, to assess and focus on the impact of low-level ambient mRNA contamination of unrelated cell population (and hence observed gene expression), on the downstream analyses. We made use of an available ground-truth cross-species dataset, where contamination can be precisely detected by identifying cross-species transcripts (https://www.10xgenomics.com/datasets/10-k-1-1-mixture-of-human-hek-293-t-and-mouse-nih-3-t-3-cells-3-v-3-1-3-1-standard-6-0-0, retrieved on 20 June 2025). More specifically, we utilised a dataset of human cells contaminated with mouse transcripts, and demonstrated that, without the correction, the contamination from mouse transcripts leads to false-positive differential expressed genes and pathway enrichments. After the correction, the cross-species ambient transcript signals were substantially reduced, leading to improved specificity in DEGs and biological pathways. **Please refer to Page 15; Line 454-467 and the new S23 - S25 Figs.**

In addition, we have also expanded the discussion to include the interesting scenarios suggested by the reviewer. **Please refer to Page 16; Line 469-476.**

Page 15; Line 454-467 and the new S23 - S25 Figs.

To further validate our findings, we leveraged a publicly available cross-species dataset from 10x Genomics (10-k-1-1-mixture-of-human-hek-293-t-and-mouse-nih-3-t-3-cells-3-v-3-1-3-1-standard-6-0-0, retrieved on 20 June 2025), in which human cells were intentionally contaminated with mouse transcripts, providing a well-defined "ground-truth" for cross-species

ambient mRNA contamination (S23 Fig). Similarly to observations in our T and B cell subpopulations, the contamination of mouse transcripts led to numerous false-positive DEGs and contributed to the presence of mouse transcripts in pathway enrichment analyses when no correction was applied (S24 - S25 Figs). After ambient mRNA correction, the cross-species contamination signals were remarkably reduced, as evidenced by using the same scales before and after correction (S24 Fig), and no mouse genes were present in the significant biological pathways (S19 Table). Additionally, one GO:BP term (GO:0002181, cytoplasmic translation) was shared between before and after contamination correction. Prior to correction, genes in this GO:BP term were exclusively (100%) mouse genes, whereas after correction, this pathway was composed of human genes (S25 Fig).

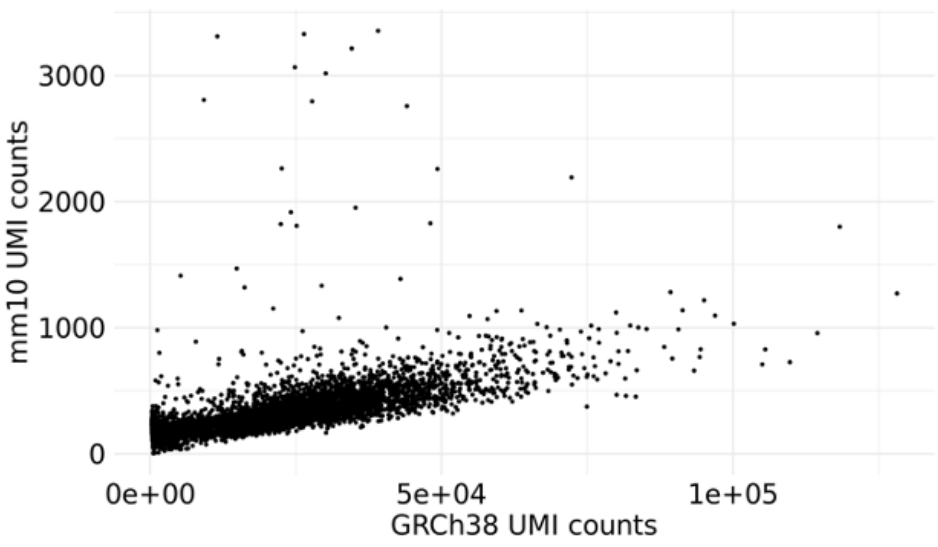

S23 Fig. Unique UMI counts per droplet that align to human (GRCh38) and mouse (mm10) gene references. Each droplet represents a human cell from a species-mixing dataset (10x Genomics; https://www.10xgenomics.com/datasets/10-k-1-1-mixture-of-human-hek-293-t-and-mouse-nih-3-t-3-cells-3-v-3-1-3-1-standard-6-0-0, retrieved on 20 June 2025), showing a low level of cross-species contamination.

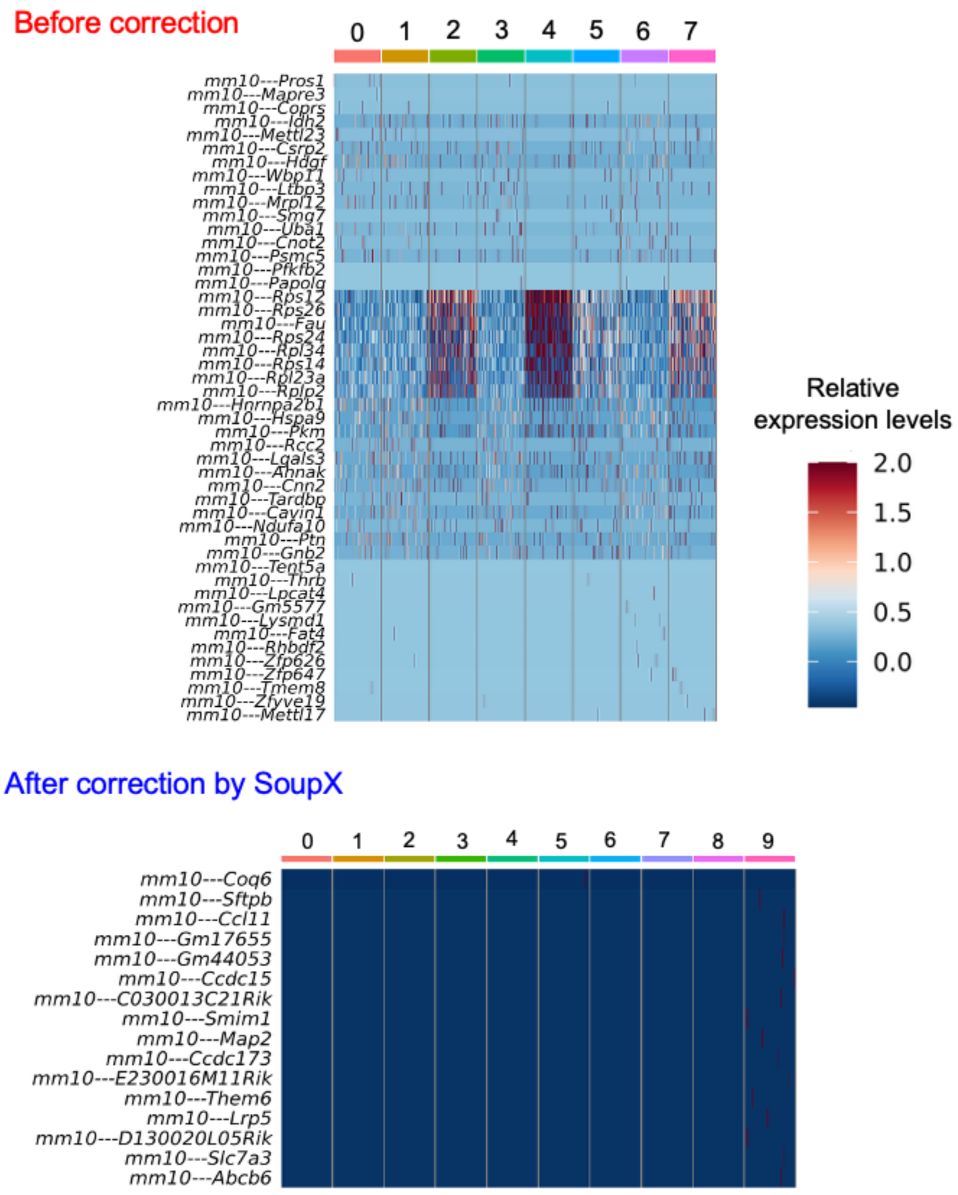

S24 Fig. Heatmap showing the relative expression levels of mouse (mm10--) genes among the top 20 differentially expressed genes (DEGs) across clusters in the human dataset from a species-mixing experiment (10x Genomics; https://www.10xgenomics.com/datasets/10-k-1-1-mixture-of-human-hek-293-t-and-mouse-nih-3-t-3-cells-3-v-3-1-3-1-standard-6-0-0, retrieved on 20 June 2025). DEGs were identified using the Wilcoxon rank sum test via the *FindAllMarker* function in Seurat V.5.2.1. Related to S21 Fig.

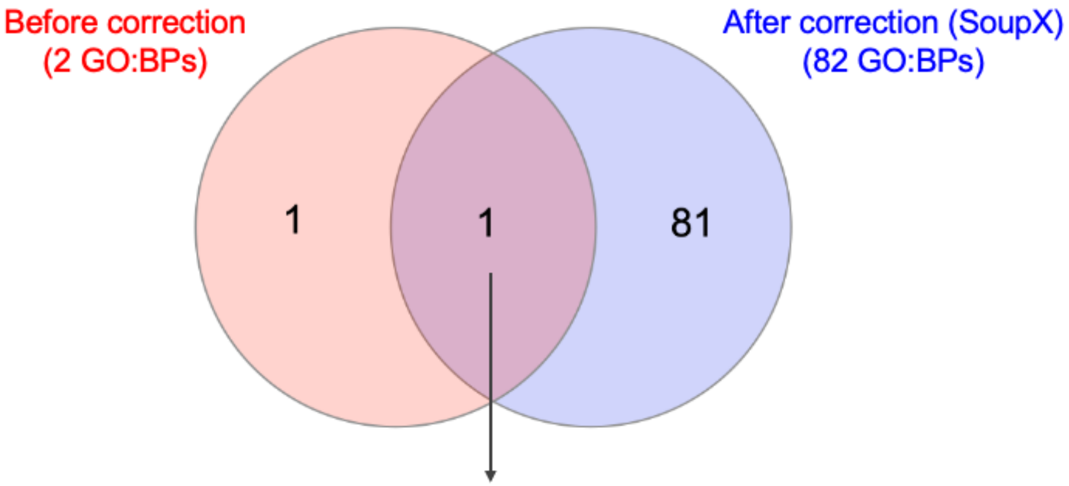

Term ID and name	Intersection genes	
	Before correction	After correction SoupX
GO:0002181 cytoplasmic translation	*mm10---Rps12, mm10---Rps26, mm10---Fau, mm10---Rps24, mm10---Rpl34, mm10---Rps14, mm10---Rpl23a, mm10---Rplp2, mm10---Pkm*	*GRCh38-RPS28, GRCh38-RPS12, GRCh38-RPL15, GRCh38-RPL36, GRCh38-RPL13, GRCh38-RPS10*

S25 Fig. Venn diagram showing the number of unique and shared Gene Ontology Biological Process (GO:BP) pathways identified before and after ambient mRNA correction with SoupX in the human dataset from a species-mixing experiment (10x Genomics; https://www.10xgenomics.com/datasets/10-k-1-1-mixture-of-human-hek-293-t-and-mouse-nih-3-t-3-cells-3-v-3-1-3-1-standard-6-0-0, retrieved on 20 June 2025). Related to S21-S22 Figs.

In addition to this, we acknowledge that a fully simulated dataset would be greatly useful for further assessing the extents of ambient RNA on analyses and have not added this to the discussion.

Page 16; Line 469-476.

Taken together, our study highlights the practical applications of mitigating ambient mRNA contamination, including improved DEG identification and enhanced specificity of cell type-specific gene expression profiles and biological pathways in biological scRNA-seq datasets. Systematic studies using simulated or controlled experimental settings - for instance, those mimicking ambient contamination arising from cell death (e.g., abnormally elevated housekeeping, ribosomal, or mitochondrial genes), or overloaded droplets - represent a promising direction for further research to investigate the impact of ambient mRNA on the downstream analyses.

3. Methods/results: for determine impact of ambient mRNA contamination in single-cell RNA-sequencing analysis on differential gene expression and biological pathway enrichment analyses, authors may need explain quantitative standards, not just using qualitative methods such as "increase/decrease"

We thank the reviewer for this important suggestion. We have included detailed quantitative comparisons of gene expression levels before and after ambient mRNA correction, reporting average expression values and percentages of expressing cells (S3-S4, S6-S7 and S10 Tables). Moreover, we have provided quantitative data on the number and significance of enriched GO:BP terms before and after correction to support our conclusions about changes in pathway analysis results. Additionally, we have included the statistical details of differential gene expression and pathway enrichment analyses in the Methods section. We believe that this quantitative information strengthens the rigor of our findings and analyses.

Materials and Methods

Please see Page 6, Line 153-173;

Differential gene expression analysis

A total of 38,606 genes were tested for differential gene expression (DEG) analysis using the Wilcoxon rank sum test implemented in the *FindAllMarkers* function of Seurat V.5.2.1 [22]. Genes were considered differentially expressed if they were expressed in at least 10% of cells in either group and met a minimum log2 fold-change threshold of 0.1 (default settings). DEGs were defined as those with an adjusted p-value < 0.01 after Bonferroni correction for multiple testing (default settings) (Fig 3A and 4A). The total numbers of genes passing these cutoffs across datasets are listed in S2 Table.

Pathway analyses

g:Profiler2 (v.0.2.3) [31] was applied to systematically assess biological processes using human reference genes from GRCh38.p14 and focusing on Gene Ontology Biological Process (GO:BP). To reduce the false positives, the false discovery rate (FDR) approach was applied for multiple testing correction, and adjusted p-values < 0.05 were considered significant. The top 20 DEGs, ranked by average log2 fold-change (avg_log2FC), were selected for each stage of T cell populations in the dengue datasets and for each seurat cluster in the 10x Genomics species-mixing dataset (10x Genomics; https://www.10xgenomics.com/datasets/10-k-1-1-mixture-of-human-hek-293-t-and-mouse-nih-3-t-3-cells-3-v-3-1-3-1-standard-6-0-0, retrieved on 20 June 2025). In the human fetal tissues, the top 2000 DEGs (ranked by average log2 fold-change) of "pro B cells" were selected. To visualise unique and overlapping GO:BP terms before and after ambient mRNA correction, Venn diagrams were constructed using InteractiVenn [32].

Results and discussion

Please see Page 7, Line 195-209;

Based on the expression of known canonical marker genes, we observed relatively high expression levels of the T cell markers, *CD3E* (encoding the CD3-epsilon polypeptide to form the T cell receptor complex) and *TRAC* (T cell Receptor Alpha Constant) in annotated T cells, with average expression ranging from 1.29 - 1.43 and up to 75% of T cells expressing the genes, compared to other clusters (average expression 0.03 - 0.82, < 54% expressing cells) (Fig 1B, S3 Table, and S2 Fig). Similarly, B cell lineage genes *MS4A1* (B-lymphocyte-specific membrane protein) and *CD79A* (B-cell antigen receptor complex-associated protein) exhibited higher expression levels (1.26 - 2.10) and were expressed in > 76.23 % of B cells and PCs, compared to other cell types (average expression < 0.14 and < 16% expressing cells) (Fig 1B, S4 Table, and S2 Fig). In contrast, certain immunoglobulin (Ig) genes (referred to as "B cell-related genes", herein), comprising *IGKC*, *IGHG1*, *IGHG4*, and *JCHAIN*, were expressed across multiple populations at relatively low expression levels (0.45 - 3.33) and in up to 99% of these cells expressing the genes, when compared to B cells and PCs expected to express these genes (average expression 1.06 - 6.08 and 62 - 100% expressing cells) (Fig 1B, S4 Table, and S2 Fig).

Please see Page 9, Line 242-249;

The transcription levels of canonical marker genes in corresponding cell types, including selected T and B cell markers, remained consistently high and relatively unchanged after correction (S6 Table and S4 Fig) with both CellBender (S6 Table and S5 Fig) and SoupX (S6 Table and S6 Fig). Specifically, the average expression levels of these genes across all cells were 0.36 before correction, 0.34 after CellBender and 0.35 after SoupX corrections (S6 Table). This suggests the robustness of the ambient mRNA correction step in accurately preserving the expression patterns of well-established cell type markers.

Please see Page 9, Line 257-263;

Conversely, after the correction, we observed significant decreases in the transcription levels of B cell-related genes in "non-B cell" populations following correction with both CellBender and SoupX (Figs 2A-2B, see S3 Fig for SoupX, and S7 Fig). The average expression levels of B cell-related genes in "non-B cells" dropped from 0.47 before correction to 0.07 after CellBender and 0.05 after SoupX correction (S7 Table). These results suggest that the correction process effectively removed the expression signals originating from ambient mRNAs.

Please see Page 10, Line 292-296;

After applying ambient mRNA correction, the expression of these Ig genes was noticeably reduced across T cells (S11 Fig for CellBender and S12 Fig for SoupX), with average expression levels decreasing from 0.54 before correction, to 0.10 after CellBender and 0.06 after SoupX (S10 Table), suggesting effective removal of background contamination.

4. Discussion: authors may need explain potential benchmakt effects for many related softwares such as DecontX, scAR

We thank the reviewer for this valuable comment. We have expanded the discussion to include other ambient mRNA removal tools and their potential impact in our revised manuscript.

Please refer to page 13, Line 377-383.

Among the 187 uniquely identified GO:BPs after CellBender correction, we observed biological pathways associated with cell cycle processes, cell division, and DNA repair (Fig 3B), which have been identified in HLA-DR^{+} $CD38^{+}$ CD8 T cells during dengue fever [35]. Additionally, we found type I interferon-related pathways (Fig 3B), which have been shown to be expressed in T cells [13, 34, 36]. These results suggest that ambient mRNA correction effectively reduced the confounding influence of Ig genes, thereby revealing a more relevant immune signature in T cell subpopulations during acute dengue infection. We noted that the choice of ambient mRNA correction tools, such as DecontX [19], FastCAR [15], and scAR [37], may yield variable results in terms of adjusted expression valves. This variability may influence the outcomes of downstream analyses, including differential gene expression (e.g., log2 fold-change values) and pathway enrichment analyses (e.g., p-values or significant pathways), as different tools make different assumptions and use different approaches for evaluating and removing ambient mRNA contamination.

Reviewer #4:

The authors applied two computational methods (SoupX and CellBender) to estimate and remove ambient mRNAs in droplet-based scRNA-seq datasets and observed reduced false positive differential expressed genes (DEGs) on 2 cell types of interests and subsequently improved biological pathways. The results in this manuscript show dataset dependent performance improvements as well as some level of persisting artifacts after using decontamination methods. This would serve as a useful reference for the field to draw analysis conclusions with caution and scrutiny on scRNAseq analysis.

We greatly appreciate the reviewer's acknowledgment of the significance of ambient mRNA contamination in single-cell data.

I appreciate the comments from Reviewer 1 and Reviewer 2 and authors' additional analysis and improved clarity. I have a few suggestions that I believe would be necessary to further improve the manuscript:

We thank the reviewer for these valuable comments, and we have tried to address them as thoroughly as possible, as detailed below.

1. Authors should report cell-level as well as dataset level contamination percentage estimated by both methods (SoupX and CellBender).
As pointed out by reviewer 2, a simulation study could further illustrate how contamination level would affect downstream analysis. Although simulation itself could potentially require the work that's beyond the scope of this manuscript, a deeper insight could be provided by reporting the estimated contamination levels by both methods and commenting on their associations with dataset dependent improvements.

We thank the reviewer for this insightful suggestion. We agree that linking estimated contamination levels to improvements in downstream analyses could be highly informative. However, we would like to clarify that direct assessment is challenging due to the following reasons:

1). Different levels of measurements: SoupX provides an "estimated global contamination fraction", which reflects dataset-level contamination, while CellBender provides the contamination fraction of each droplet (cell-level contamination), making direct comparison between the two approaches difficult.

2). Workflow separation: Ambient mRNA removal, including the estimation of contamination fractions, was performed on individual samples prior to integration, whereas

differential gene expression and pathway enrichment analyses were performed on integrated datasets that combine all samples.

Nevertheless, we have added a species-mixing dataset, where known cross-species can be precisely detected, as also described in details above. This provides insight into the effectiveness of ambient mRNA correction, our analysis pipelines, and the resulting key findings.

Page 15; Line 454-467 and the new S23 - S25 Figs.

To further validate our findings, we leveraged a publicly available cross-species dataset from 10x Genomics (10-k-1-1-mixture-of-human-hek-293-t-and-mouse-nih-3-t-3-cells-3-v-3-1-3-1-standard-6-0-0, retrieved on 20 June 2025), in which human cells were intentionally contaminated with mouse transcripts, providing a well-defined "ground-truth" for cross-species ambient mRNA contamination (S23 Fig). Similarly to observations in our T and B cell subpopulations, the contamination of mouse transcripts led to numerous false-positive DEGs and contributed to the presence of mouse transcripts in pathway enrichment analyses when no correction was applied (S24 - S25 Figs). After ambient mRNA correction, the cross-species contamination signals were remarkably reduced, as evidenced by using the same scales before and after correction (S24 Fig), and no mouse genes were present in the significant biological pathways (S19 Table). Additionally, one GO:BP term (GO:0002181, cytoplasmic translation) was shared between before and after contamination correction. Prior to correction, genes in this GO:BP term were exclusively (100%) mouse genes, whereas after correction, this pathway was composed of human genes (S25 Fig).

Page 16; Line 469-476.

Taken together, our study highlights the practical applications of mitigating ambient mRNA contamination, including improved DEG identification and enhanced specificity of cell type-specific gene expression profiles and biological pathways in biological scRNA-seq datasets. Systematic studies using simulated or controlled experimental settings - for instance, those mimicking ambient contamination arising from cell death (e.g., abnormally elevated housekeeping,

ribosomal, or mitochondrial genes), or overloaded droplets - represent a promising direction for further research to investigate the impact of ambient mRNA on the downstream analyses.

2. 2000 DEGs are a lot for a subpopulation – i.e. a highly functionally specific cell type ("top 2000 DEGs in pro-B cells" Page 6 Line 156). And this result is even more concerning when they are only the positive set of the DEGs ("the positive markers were retained (Fig 3A and 4A)." Page 6 Line 148) – implying the whole DEG set including both the positively and negatively differentially expressed gene pool is even larger. Can the authors explain whether this is biologically pelausible? In addition, authors should report the number of genes used for performing DE analysis as well as the resulting number of DEGs.

We thank the reviewer for this important point. To clarify, the 2,000 genes were not the final marker genes or reported as biologically unique to this subpopulation, but rather were selected for pathway enrichment analysis based on the top average log2 fold-change values to ensure adequate pathway coverage and statistical power in enrichment analysis

To address the concern, we re-evaluated the differential gene expression (DEG) analysis without restricting it to only the positively regulated genes, thereby including both upregulated and downregulated genes. Indeed, the number of positive marker genes remained similar, regardless of this setting. Consequently, the downstream pathway analysis remains unchanged, as input genes were selected based on the top average log2 fold change values.

We have now added the total number of genes tested for DEGs, as well as the number of DEGs that met the statistical significance threshold (including both positively and negatively DEGs) (S2 Table), as suggested. Additionally, we have revised and improved the clarity of the "Differential gene expression analysis" section in the Methods to provide these statistical details.

Please see Page 6, Line 153-173;

Differential gene expression analysis

A total of 38,606 genes were tested for differential gene expression (DEG) analysis using the Wilcoxon rank sum test implemented in the *FindAllMarkers* function of Seurat V.5.2.1 [22]. Genes were considered differentially expressed if they were expressed in at least 10% of cells in either group and met a minimum log2 fold-change threshold of 0.1 (default settings). DEGs were defined as those with an adjusted p-value < 0.01 after Bonferroni correction for multiple testing (default

settings) (Fig 3A and 4A). The total numbers of genes passing these cutoffs across datasets are listed in S2 Table.

S2 Table. The total numbers of DEGs

T cell subpopulations			
	The number of DEGs		
	All DEGs	avg_log2FC > 0	avg_log2FC < 0
Before ambient mRNA correction	23,386	11,059	12,327
SoupX correction	23,206	10,955	12,251
CellBender correction	22,470	10,679	11,791
**B cell subpopulations**			
	The number of DEGs		
	All DEGs	avg_log2FC > 0	avg_log2FC < 0
Before ambient mRNA correction	27,686	16,133	11,553
SoupX correction	27,699	15,832	11,867
CellBender correction	27,805	16,257	11,548
**Pro B cells**			
	The number of DEGs		
	All DEGs	avg_log2FC > 0	avg_log2FC < 0
Before ambient mRNA correction	7,265	5,802	1,463
SoupX correction	7,129	5,672	1,457
CellBender correction	7,290	5,888	1,402

3. Fig S9 and S19 provided relative cell-type composition information but lacked absolute value information – the number of cells in each type before and after ambient mRNA correction. Cell-type assignment could change after correction, and it would be informative to show the magnitude of such changes for both methods given two very different methodological approaches between SoupX and CellBender.

Thanks for this valuable comment. The absolute numbers of annotated cell types remain largely consistent before and after ambient mRNA correction (R1 and R2). We have included supplementary tables (S9 Table) showing the absolute numbers of cells in each cell type in our revised manuscript.

Please see Page 10, Line 287-289;

The number of annotated T cells remained relatively comparable before and after ambient mRNA correction (S9 Table and S9 Fig), suggesting that contamination did not impact the annotation.

Please see Page 14, Line 405-407;

Similar to T cell subsets, the number of annotated B cell subpopulations remained relatively comparable before and after ambient mRNA correction (Sx8 Table and S19 Fig).

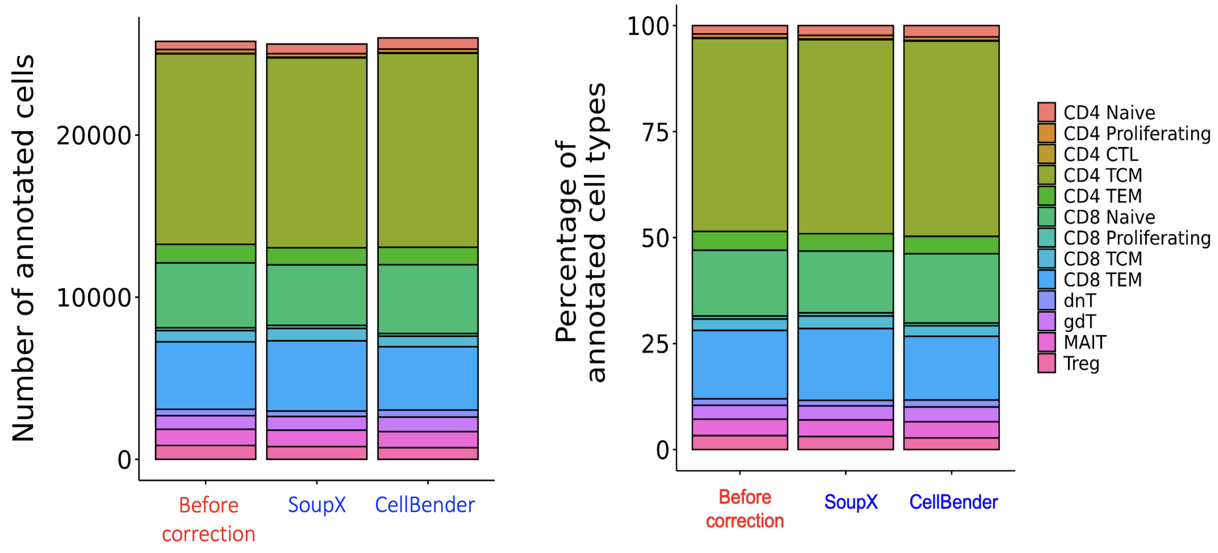

R1 Fig. Bar plots showing the absolute numbers (left panel) and percentages (right panel) of annotated T cell subpopulations, based on Azimuth and supplemented by known canonical marker genes, before and after correction.

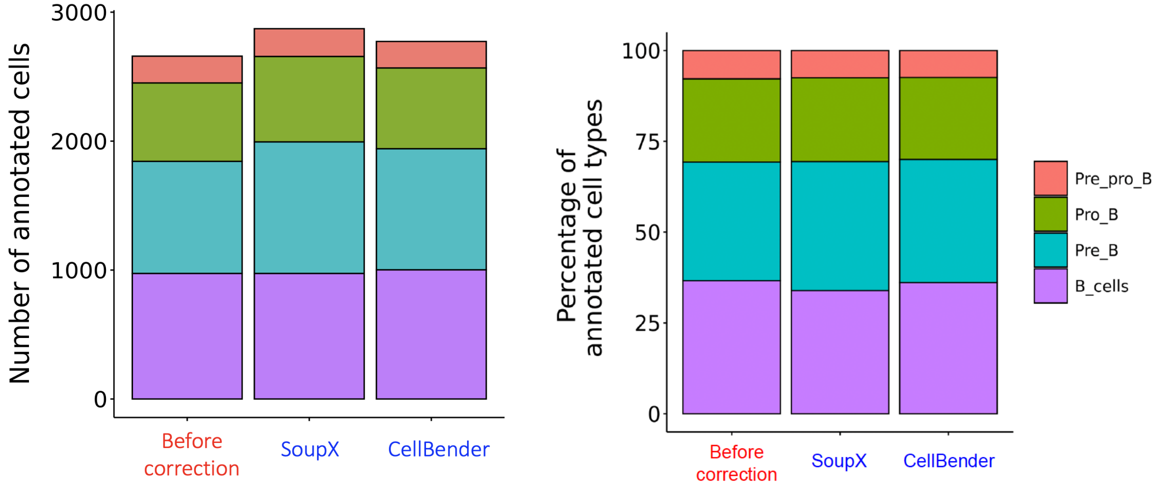

R2 Fig. Bar plots showing the absolute numbers (left panel) and percentages (right panel) of annotated B cell subpopulations, based on Azimuth and the original article's annotation [21]. Colors represent different cell types.
