# Supplementary material for: Understanding and mitigating the impact of ambient mRNA contamination in single-cell RNA-sequencing analysis
Source: PLoS One. 2025 Sep 24;20(9):e0332440. doi: 10.1371/journal.pone.0332440 (PMC12459771; doi:10.1371/journal.pone.0332440)
Supplement: Attachment — Submitted filename: Response_to_Reviewers_auresp_3.pdf [file pone.0332440.s025.pdf]

Dear Academic Editor and Reviewers,

Thank you for your comments and for the opportunity to revise our manuscript. We have addressed the following.

Journal Requirements:

1. If the reviewer comments include a recommendation to cite specific previously published works, please review and evaluate these publications to determine whether they are relevant and should be cited. There is no requirement to cite these works unless the editor has indicated otherwise.

The reviewers did not suggest any specific previously published works in this revision.

2. Please review your reference list to ensure that it is complete and correct. If you have cited papers that have been retracted, please include the rationale for doing so in the manuscript text, or remove these references and replace them with relevant current references. Any changes to the reference list should be mentioned in the rebuttal letter that accompanies your revised manuscript. If you need to cite a retracted article, indicate the article's retracted status in the References list and also include a citation and full reference for the retraction notice.

We have reviewed all cited references to ensure they are complete and accurate. None of the references cited in our manuscript have been retracted to our knowledge.

6. Review Comments to the Author

Reviewer #3:

Last question remarks for this manuscript were mostly addressed:

1. Introduction: Add a new example;
2. Methods: include a new ambient mRNA correction method;
3. Methods/Results: statistical methods were used in celltype annotation/DE analysis/pathway enrichment;
4. Add a new validation dataset.

We thank the reviewer for their thoughtful and insightful feedback. We appreciate the acknowledgment that the majority of the previous concerns have been addressed, and we believe that these revisions have strengthened the manuscript significantly.

Reviewer #4:

Thanks to the authors addressing clearly to my comments.

There is a minor clarification I would request authors to state in the manuscript about log-fold change threshold: It was stated DE analysis's criterium on log-fold change is 0.1, "a minimum log2 fold-change threshold of 0.1 " (Page 6 Line 157). for the purpose of examining false positive DE genes in the context of this manuscript is a okay. However this should not be used in general to draw biological conclusions upon as log2 ratio being 0.1 means the expression ratio of the 2 compared groups is 1.0717, i.e. one group has about 7% higher expression than the other which is extremely low. Hence authors should state in the manuscript that this threshold 0.1 was chosen to have a larger gene pool to show ambient RNAs' bias effect on DE analysis rather as the normal standard for DE analysis.

We thank the reviewer for this helpful suggestion. We would like to clarify that the log2 fold-change threshold of 0.1 was not manually selected, but is a default parameter in Seurat's *FindAllMarkers* function (please see also: https://satijalab.org/seurat/reference/findallmarkers). **This threshold was only applied during the initial differential expression testing to identify a broad pool of candidate genes and was not used to filter genes for biological pathway analyses**. The actual log2 fold-change of the top 20 DEGs identified were between 1.7 to 6.9 before correction, and 3.0 to 19.9 after SoupX correction for the species-mixing dataset, for instance. Log2 fold-changes of top DEGs in other datasets do vary but they are all higher than the initial cut-off of 0.1 used in the test.

To avoid any confusion and improve clarity for readers, we have revised the Methods section to clearly distinguish between DEG detection thresholds and gene selection criteria for biological pathway analyses. Specifically, DEGs used for pathway analyses were selected based on the top genes ranked by average log2 fold-change, without applying a fixed fold-change threshold. Please refer to **Page 6 Line 159-161 and Line 168-172 for the updated text.**

Page 6 Line 159-161:

Differential gene expression analysis

A total of 38,606 genes were tested for differential gene expression (DEG) analysis using the Wilcoxon rank sum test, implemented in the *FindAllMarkers* function of Seurat V.5.2.1 [22]. Genes were considered differentially expressed using Seurat's default parameters – specifically, they were expressed in at least 10% of cells in either group and had a minimum log2 fold-change threshold of 0.1. DEGs were defined as those with an adjusted p-value < 0.01 after Bonferroni correction for multiple testing (also using default settings). These default parameters were used for the initial identification of DEGs and were not intended to define biologically meaningful

expression changes. The total numbers of DEGs passing these cutoffs across datasets are listed in S2 Table.

Page 6 Line 168-172:

Pathway analyses

g:Profiler2 (v.0.2.3) [31] was applied to systematically assess biological processes using human reference genes from GRCh38.p14 and focusing on Gene Ontology Biological Process (GO:BP). To reduce the false positives, the false discovery rate (FDR) approach was applied for multiple testing correction, and adjusted p-values < 0.05 were considered statistically significant. For GO:BP analyses, DEGs were selected by ranking genes based on average log2 fold-change (avg_log2FC), without applying a fixed fold-change threshold. This rank-based approach priorities the most biologically distinct genes and avoids arbitrarily strict cutoffs, which may be too stringent — particularly when comparing results before and after ambient mRNA correction. The top 20 DEGs (ranked by avg_log2FC) were selected in the dengue datasets and for each seurat cluster in the 10x Genomics species-mixing dataset (10x Genomics; https://www.10xgenomics.com/datasets/10-k-1-1-mixture-of-human-hek-293-t-and-mouse-nih-3-t-3-cells-3-v-3-1-3-1-standard-6-0-0, retrieved on 20 June 2025). In the human fetal tissues, the top 2000 DEGs of "pro B cells" were selected. To visualise unique and overlapping GO:BP terms before and after ambient mRNA correction, Venn diagrams were constructed using InteractiVenn [32].
