# Supplementary material for: Understanding and mitigating the impact of ambient mRNA contamination in single-cell RNA-sequencing analysis
Source: PLoS One. 2025 Sep 24;20(9):e0332440. doi: 10.1371/journal.pone.0332440 (PMC12459771; doi:10.1371/journal.pone.0332440)
Supplement: Attachment — Submitted filename: comments.docx [file pone.0332440.s021.docx]

I appreciate the opportunity to review this manuscript titled ' Understanding and mitigating the impact of ambient mRNA contamination in single-cell RNA-sequencing analysis'. The study addresses an important topic in single cell RNA-sequencing, which is relevant to advancing our understanding of how ambient noise interferes with downstream analyses. The authors have made commendable efforts in investigating the impacts of ambient mRNA noise in scRNA-seq analysis. Below, I provide my detailed comments and suggestions to help enhance the clarity, rigor, and overall quality of the manuscript.

**Major comments:**

1. The authors claim that they “investigate the impact of ambient mRNA contamination on downstream bioinformatic analyses” using two datasets. For this, they should provide a comprehensive description of noise level removed by SoupX in the datasets analyzed. No objective criteria were introduced to measure the level of noise in the datasets. Instead, the authors claimed that the two dataset they have studied had “high” level of noise.
2. I suggest that the authors simulate different unbiased levels of ambient noise and add these noise levels to a cleaned dataset and then compare how different levels of noise affects the downstream analyses.

Section 3.1:

3. The authors claim that the dataset used for this section exhibits high levels of mRNA contamination. This is very subjective. If there is a measure of contamination which they have used to screen datasets for the high level of noise, it should be mentioned.

Section 3.2:

4. The use of SoupX because of its convenience is not relevant. Using SoupX is highly subjective and depends on domain knowledge and user input. The authors could use CellBender to do ambient noise removal for it provides an unbiased processing.
5. Instead of exploring pre-selected set of genes like Ig or Hb genes, the authors should provide the top genes with high contamination fraction and select form those.

**Technical comments:**

1. The overall bioinformatic analyses performed for this manuscript are weak.
2. There are better doublet identification methods like scDblFinder.
3. No batch information was provided for any of the datasets. The UMAPs with batch information before and after batch effect correction could be supplemented.
4. The choice of Wilcox rank sum test for DE is not appropriate. This test is associated with high rate of false positive results. Use of pseudobulk is recommended. Refer to PMID: 34584091.
5. No objective criteria were used to assess and compare clustering quality before and after noise removal. Silhouette score or Davies-Bouldin Index could be used.

**Minor comments**:

Section 3.1:

1. the authors have called cell types based on the module scores of some canonical markers for each cell types and cells with scores > 0 were called. The authors should demonstrate that how robust is this cell typing to the presence of the ambient mRNA.
2. Line 200. Presence of CD3 and TRAC among markers makes the topic for figure 1 inappropriate.
3. Lines 205 to 211 are not adding any information to what was mentioned in lines 60-65 in introduction.

Section 3.2:

4. In Figure 2, the authors must use the same UMAP coordinates for cells before and after correction so that it would help reader to compare the expression levels before and after correction in feature plots.
5. Lines 238-246: the authors should discuss the remaining 2 modal expression of IGKC and IGHG4 which is still present after correction in B cells. The authors should provide the total number of cells and number of cells expressing these two markers before and after correction in both non-B and B cells with the violin plots.

Section 3.4:

6. In the second dataset, the cell compositions should be explained.
